# Supplementary material for: Estimation of absolute states of human skeletal muscle via standard B-mode ultrasound imaging and deep convolutional neural networks
Source: J R Soc Interface. 2020 Jan 29;17(162):20190715. doi: 10.1098/rsif.2019.0715 (PMC7014797; doi:10.1098/rsif.2019.0715)
Supplement: Supplementary material [file rsif20190715supp1.docx]

Estimation of Absolute States of Human Skeletal Muscle *via* Standard B-Mode Ultrasound Imaging and Deep Convolutional Neural Networks

Ryan J. Cunningham, Member*, IEEE*, and Ian D. Loram, Member, IEEE

|  |
| --- |
| **Figure A Supplementary Material.**  Muscle tissue comprises muscle fibres embedded within a collagenous endomysia network. This dynamic 3-dimensional structure, observable by ultrasound (US) as shape and texture as shown in this figure. White (echogenic) material is primarily the collagen structure of the muscle. Black (hypo echogenic) is muscle fibres. On account of the water content, muscle *per se* appears dark. Muscle tissue generates force, which is transmitted through this collagen structure along the distributed curvilinear path between origin and insertion of each muscle. We hypothesize the dynamic state of skeletal muscle, is encoded by the 3D-collagenous structure, and is observable by 2D US images.  Two main independent inputs, neural excitation and length (origin-insertion distance caused by joint ankle), determine the dynamic state of muscle. Other factors determining the current state include local history of activation, distance moved by the muscle since it was last still, and pressure of external structure such as other muscles, but in general these factors are more minor. The general condition of the muscle (size, health, injury, inflammation, collagen content) etc. is also important. Variation between individuals will alter the translation of input to muscle force.  The muscle state is termed “neurobiomechanical” because the state vector comprises one neural (activity) and two biomechanical (length, tension) components, defined here as (activity, joint angle, joint moment). Neural drive causes metabolically active contraction in muscle fibres. This internally generated pattern of tension contracts the internal collagenous structure, which shortens the muscle tissue and stretches the tendon tissue connecting muscle to bone. Joint angle reflects external forces (gravitational, contact, and inertial) imposed on muscle. External force stretches the collagenous structure passively from outside and lengthens both the muscle and tendon. Because of the different active v passive force transmission patterns, we hypothesize the three components (activity, length, tension) are encoded instantaneously and independently within the collagenous structure and in a form generalizable between individuals. |
| 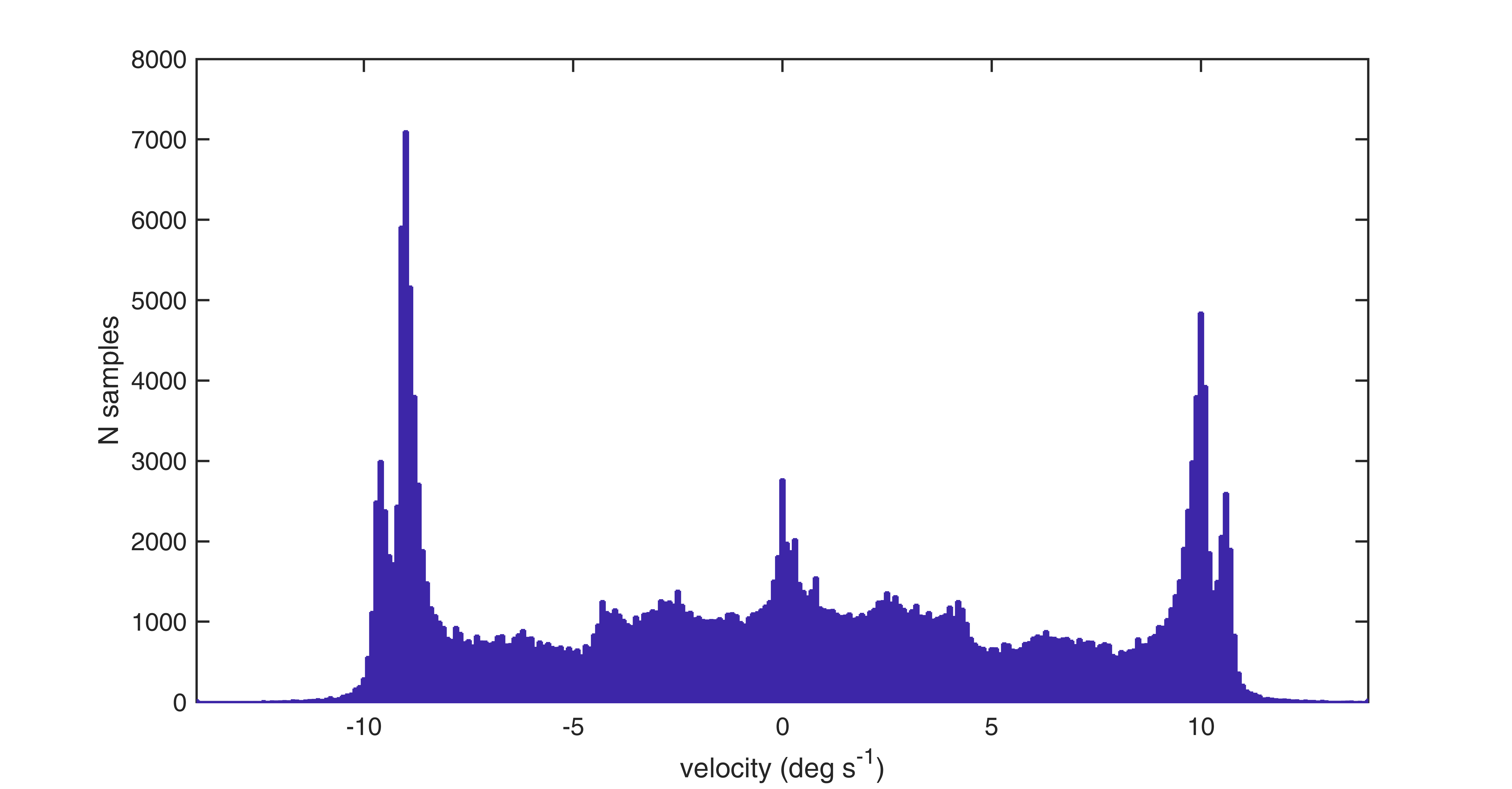 |
|  |

**Figure B Supplementary Material.**

This figure shows the distribution of ankle joint rotation velocities used during passive and combined trials.

| 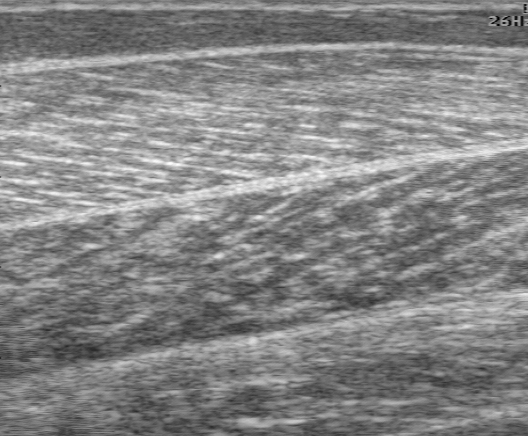 |
| --- |
| **Video 1. Supplementary video media.** This figure is an accompaniment to the supplementary video file we have supplied. It represents an ultrasound image taken from the video sequence (25 Hz). The video shows real data (triceps surae) from a single participant during one of the ‘combined’ ankle rotation and active contraction trials. This video is given to present and illustrate the challenge addressed in this study – “given a reference frame, from any single image in the sequence, predict the EMG (mV) of GM and SO muscles, the combined force (Nm) of both muscles acting on the ankle, and the joint angle (degrees) of the ankle”. It is implausible or perhaps impossible for a human to assess a single image and accurately estimate those variables under these complex combined conditions. That is the technical challenge addressed in this study, which has utilised deep learning methods and advanced segmentation methods to solve it. |

| **Table A. Neural network individual test results.** Here we present test results for each participant in the data set, where the results for any single participant were generated from a neural network which was trained only on the other participants’ data (i.e. the results here all represent genuine held-out, generalized, out-of-sample results).  We present mean, standard deviation, median, minimum and maximum values for Symmetric Mean Absolute Percentage Accuracy (100-SMAPE) and Mean Absolute Error (MAE), calculated for each participant, for each of the 4 labels: EMG (GM/SO), ankle joint moment, and ankle joint angle (JA). Samples indicates images tested per participant. | | | | | | | | | |
| --- | --- | --- | --- | --- | --- | --- | --- | --- | --- |
| **Participant** | **Samples** | **Symmetric Mean Absolute Percentage Accuracy**  **(100 - SMAPE)** | | | | **Mean Absolute Error (MAE)** | | | |
|  |  | **EMG** | |  |  | **EMG** | |  |  |
|  |  | **GM (%)** | **SO (%)** | **Moment (%)** | **JA (%)** | **GM (mV)** | **SO (mV)** | **Moment (Nm)** | **JA (°)** |
| 1 | 13,954 | 64.24 | 52.16 | 53.74 | 57.62 | 2.93 | 2.62 | 2.93 | 2.13 |
| 2 | 13,956 | 62.82 | 41.48 | 35.59 | 49.24 | 3.23 | 2.06 | 7.83 | 1.64 |
| 3 | 8,061 | 65.58 | 53.92 | 49.12 | 44.08 | 2.51 | 4.16 | 6.72 | 4.77 |
| 4 | 8,070 | 57.04 | 32.10 | 46.27 | 67.18 | 2.03 | 3.00 | 4.10 | 2.02 |
| 5 | 13,924 | 56.45 | 53.13 | 50.05 | 46.43 | 3.70 | 1.85 | 5.20 | 1.44 |
| 6 | 13,943 | 51.86 | 52.07 | 44.64 | 50.39 | 3.39 | 3.37 | 4.72 | 1.86 |
| 7 | 13,913 | 42.49 | 38.94 | 58.43 | 59.92 | 3.21 | 1.37 | 4.06 | 2.45 |
| 8** | 13,952 | 63.30 | 28.63 | 56.43 | 67.30 | 3.14 | 1.80 | 3.38 | 1.06 |
| 9 | 9,303 | 55.69 | 58.20 | 50.56 | 46.34 | 2.80 | 3.98 | 6.64 | 2.28 |
| 10 | 13,958 | 63.94 | 41.93 | 39.36 | 60.41 | 2.75 | 1.43 | 6.02 | 1.55 |
| 11 | 13,947 | 66.89 | 54.81 | 54.40 | 51.56 | 2.37 | 7.56 | 15.97 | 2.67 |
| 12 | 13,940 | 66.94 | 35.87 | 50.71 | 55.48 | 2.68 | 1.80 | 4.70 | 1.67 |
| 13 | 13,946 | 70.42 | 42.89 | 41.32 | 49.25 | 2.35 | 3.52 | 10.76 | 2.37 |
| 14 | 8,064 | 55.62 | 46.71 | 44.33 | 60.69 | 3.58 | 1.47 | 7.24 | 2.35 |
| 15 | 13,891 | 54.12 | 46.22 | 44.95 | 53.74 | 4.40 | 1.40 | 4.46 | 1.57 |
| 16 | 13,958 | 61.69 | 51.27 | 46.00 | 43.33 | 2.92 | 4.04 | 8.80 | 3.26 |
| 17 | 13,948 | 71.74 | 45.51 | 55.37 | 65.72 | 2.16 | 2.31 | 6.75 | 1.26 |
| 18 | 13,941 | 67.51 | 65.79 | 61.54 | 65.45 | 2.74 | 1.21 | 3.58 | 1.23 |
| 19 | 13,943 | 36.08 | 68.86 | 42.66 | 65.48 | 4.63 | 0.79 | 2.58 | 1.08 |
| 20 | 13,958 | 47.30 | 36.35 | 27.89 | 58.59 | 3.64 | 2.29 | 5.95 | 1.47 |
| 21 | 13,951 | 45.09 | 34.11 | 27.49 | 46.33 | 4.56 | 0.81 | 6.80 | 2.87 |
| 22 | 8,060 | 43.50 | 39.47 | 38.12 | 47.38 | 2.66 | 4.86 | 6.24 | 3.19 |
| 23 | 8,058 | 72.45 | 39.22 | 52.58 | 59.53 | 2.47 | 2.90 | 13.22 | 2.50 |
| 24* | 8,067 | 65.80 | 51.39 | 60.59 | 65.44 | 2.37 | 3.35 | 4.94 | 2.12 |
| 25 | 13,960 | 54.88 | 50.44 | 46.20 | 57.74 | 4.24 | 2.36 | 2.74 | 1.12 |
| 26 | 13,947 | 65.25 | 39.70 | 52.39 | 53.90 | 2.83 | 2.07 | 4.58 | 2.16 |
| 27 | 8,060 | 39.95 | 34.51 | 48.37 | 62.88 | 0.59 | 1.37 | 3.53 | 2.22 |
| 28 | 12,485 | 62.77 | 45.15 | 55.90 | 53.63 | 1.64 | 1.08 | 4.55 | 4.06 |
| 29 | 14,964 | 35.61 | 24.26 | 35.08 | 44.25 | 5.84 | 2.02 | 8.77 | 4.37 |
| 30 | 14,966 | 37.17 | 69.06 | 32.65 | 43.82 | 4.34 | 5.27 | 9.57 | 6.17 |
| 31 | 14,971 | 50.71 | 37.88 | 46.82 | 47.10 | 3.17 | 0.55 | 3.94 | 5.14 |
| 32 | 14,964 | 65.98 | 57.18 | 53.46 | 46.09 | 2.19 | 4.97 | 4.26 | 4.07 |
| *2^nd^ ranked result, **10^th^ ranked result: ranking was achieved by mean SMAPE over the 4 labels per participant | | | | | | | | | |

|  |
| --- |

| **Results from All Participants** |
| --- |

The file ‘AllParticipants.pdf’ shows the results for each participant.

Pages 1-64 shows times series and coherence for each participant.

Pages 1, 3, 5, …..63: Replicating Figure 4 from the ms. for each participant. Each panel compares neural network output (blue) with labels (green) for a single participant over all 3 trial conditions (combined: left, isometric: middle, and passive: right). Units are mV, Nm and degrees for EMG, Moment and Joint Angle (JA) relative to the reference frame for each section (combined, isometric, passive).

Pages 2, 4, 6, …64: Each panel shows the coherence between neural network output and labels for Gastrocnemius EMG, soleus EMG; Moment and Joint Angle. Coherence 1 means neural network output and labels maintain a constant magnitude and phase relationship. Coherence zeros means there is no linear relationship. The horizontal dotted line indicates significance at p<0.05, using a window size of 20s.

Page 65-96. Replicating Figure 5. Zoom portion of a representative participant from the ms for each participant. The zoom shows a shorter duration of contiguous data from a single participant during a trial constructing combined, independent modulations of ankle joint angle, and GM/SO EMG. Units are mV, Nm and degrees for EMG, Moment and Joint Angle (JA)
