# Supplementary material for: Estimation of absolute states of human skeletal muscle via standard B-mode ultrasound imaging and deep convolutional neural networks
Source: J R Soc Interface. 2020 Jan 29;17(162):20190715. doi: 10.1098/rsif.2019.0715 (PMC7014797; doi:10.1098/rsif.2019.0715)
Supplement: All Participant Data [file rsif20190715supp3.pdf]

Participant 1

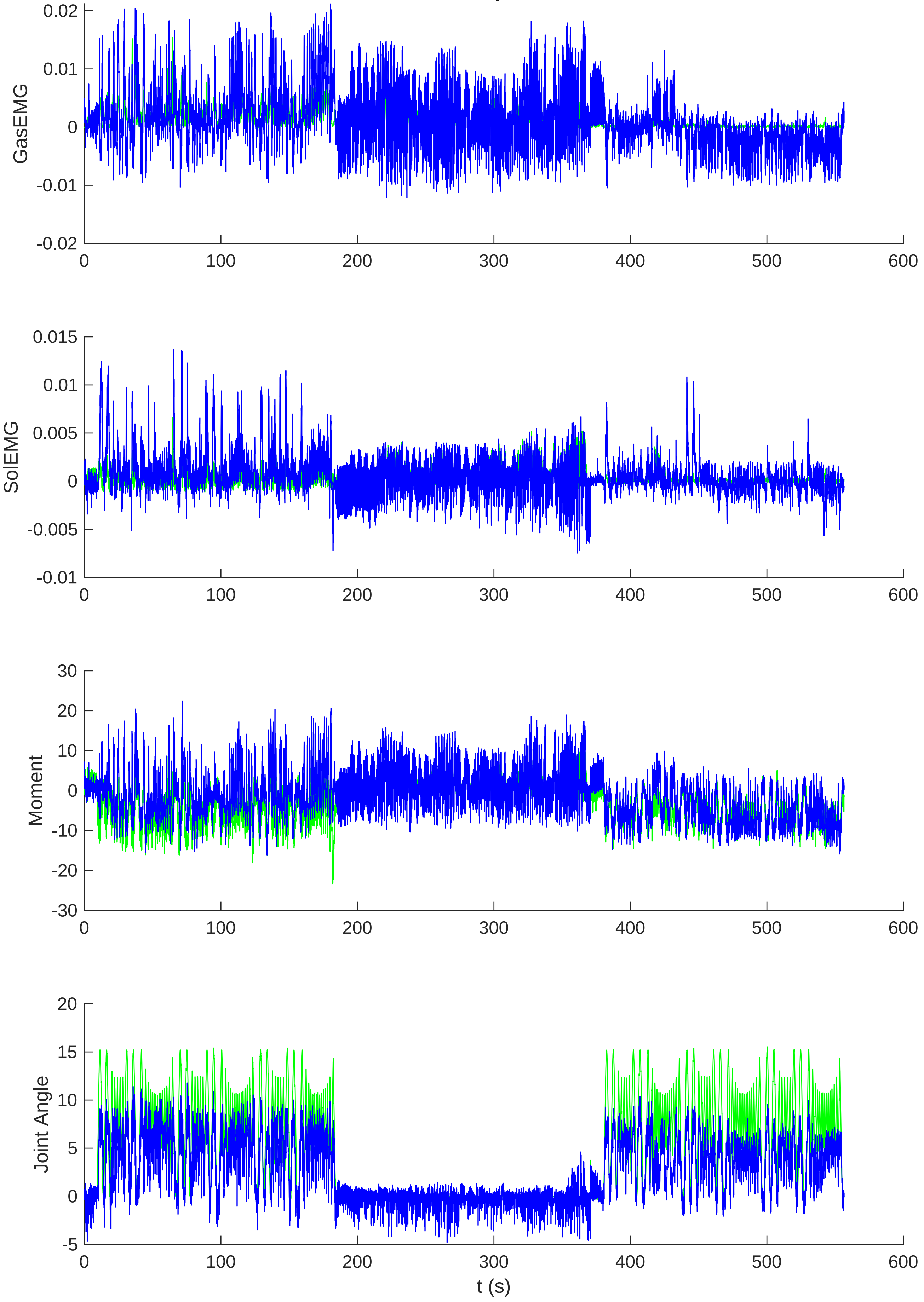

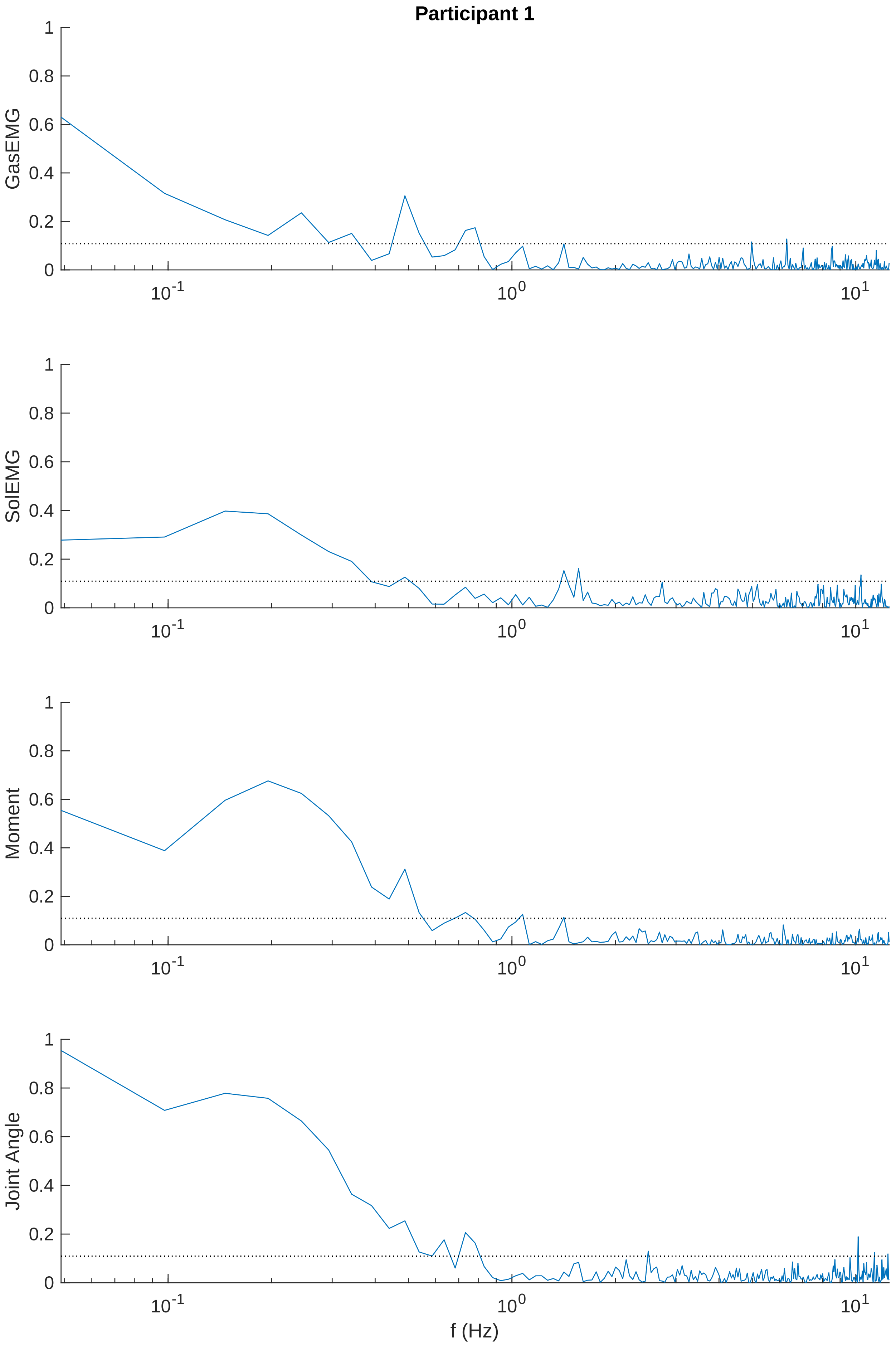

Participant 1
GasEMG
SolEMG
Moment
Joint Angle
1
0.8
0.6
0.4
0.2
0
10^{-1}
10^{0}
10^{1}
f (Hz)

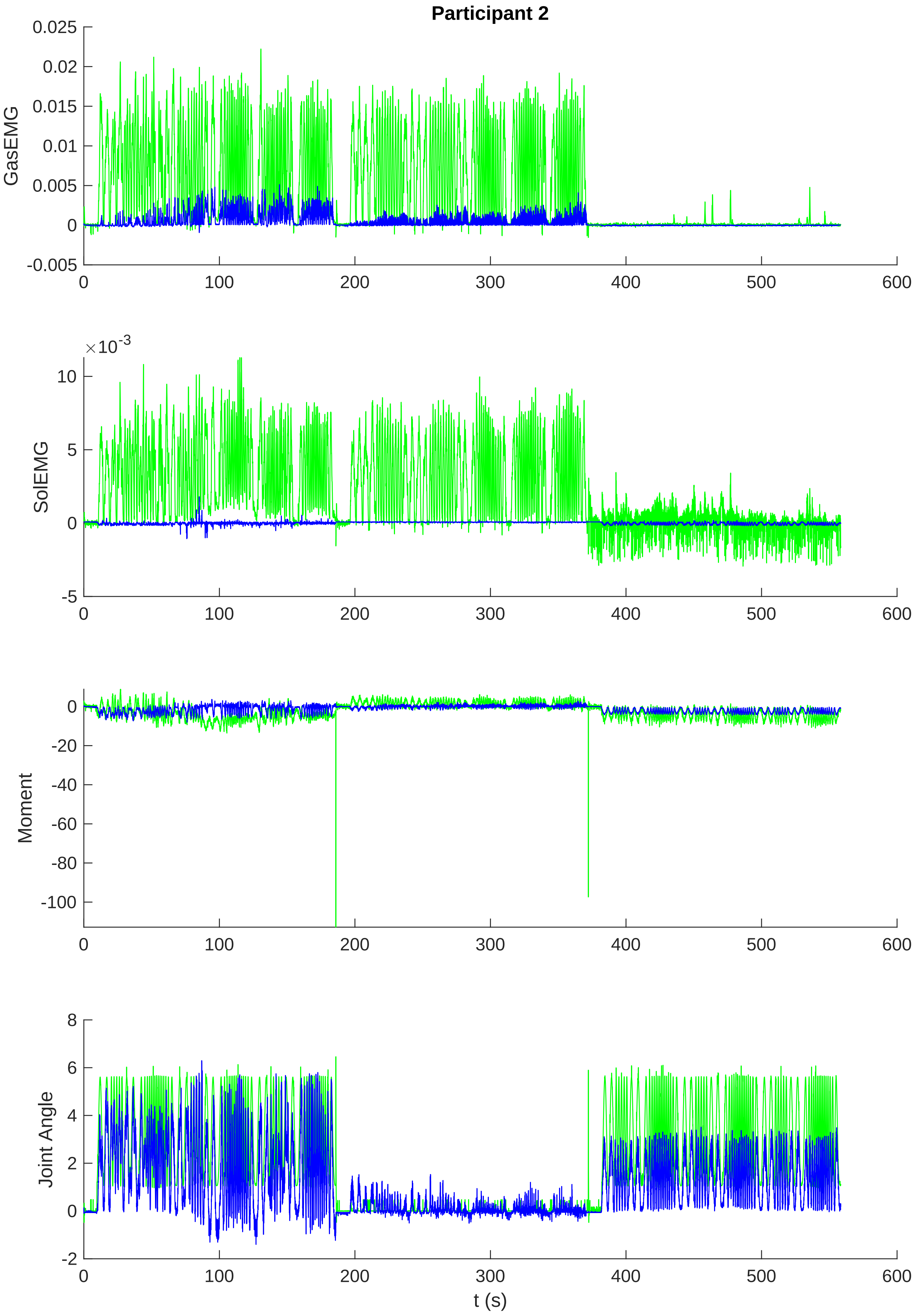

Participant 2
GasEMG
0.025
0.02
0.015
0.01
0.005
0
-0.005
0
100
200
300
400
500
600
SolEMG
×10^-3
10
5
0
-5
Moment
0
-20
-40
-60
-80
-100
Joint Angle
8
6
4
2
0
-2
t (s)

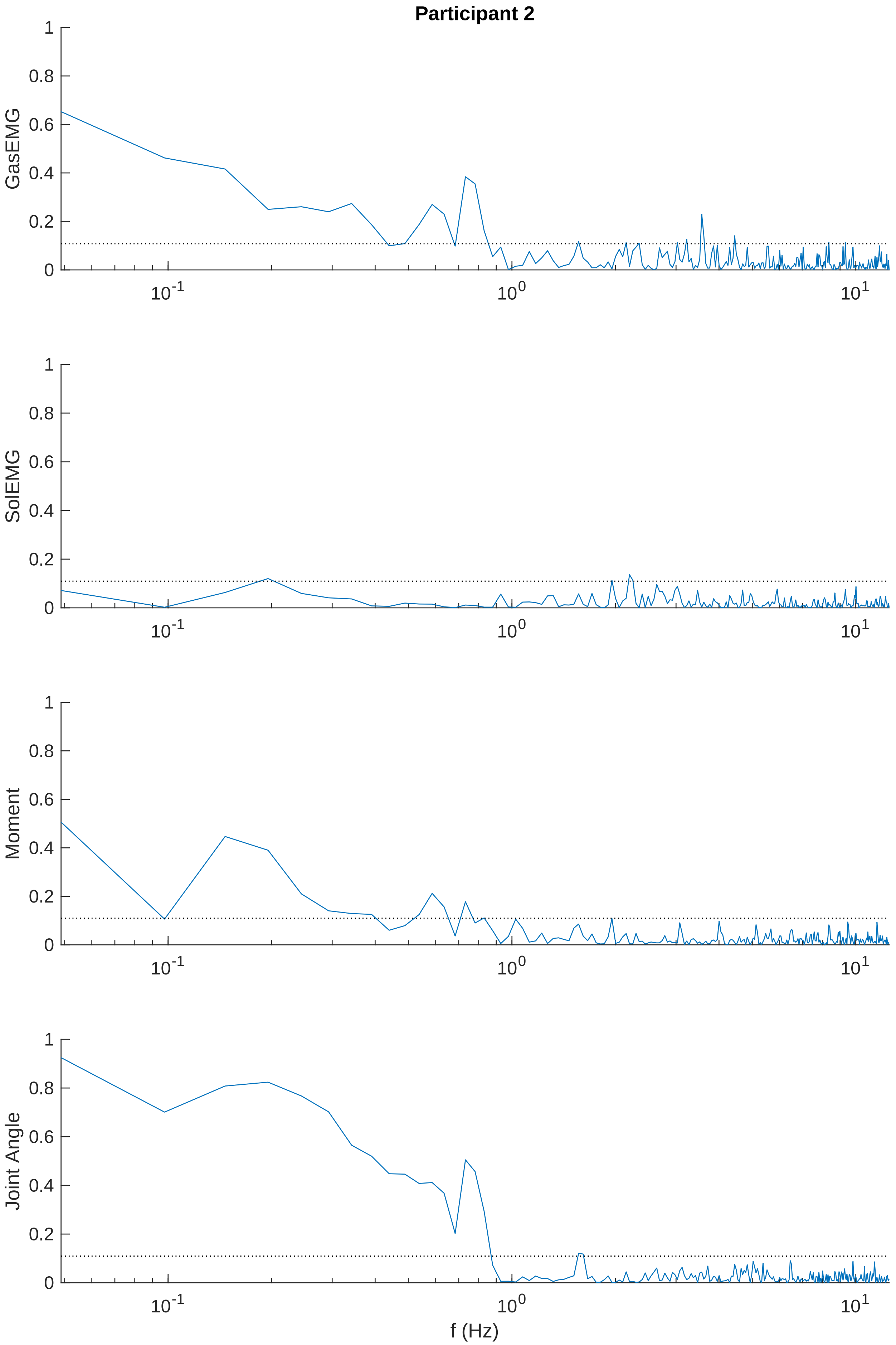
Participant 2
GasEMG
SolEMG
Moment
Joint Angle
1
0.8
0.6
0.4
0.2
0
10-1
100
101
f (Hz)

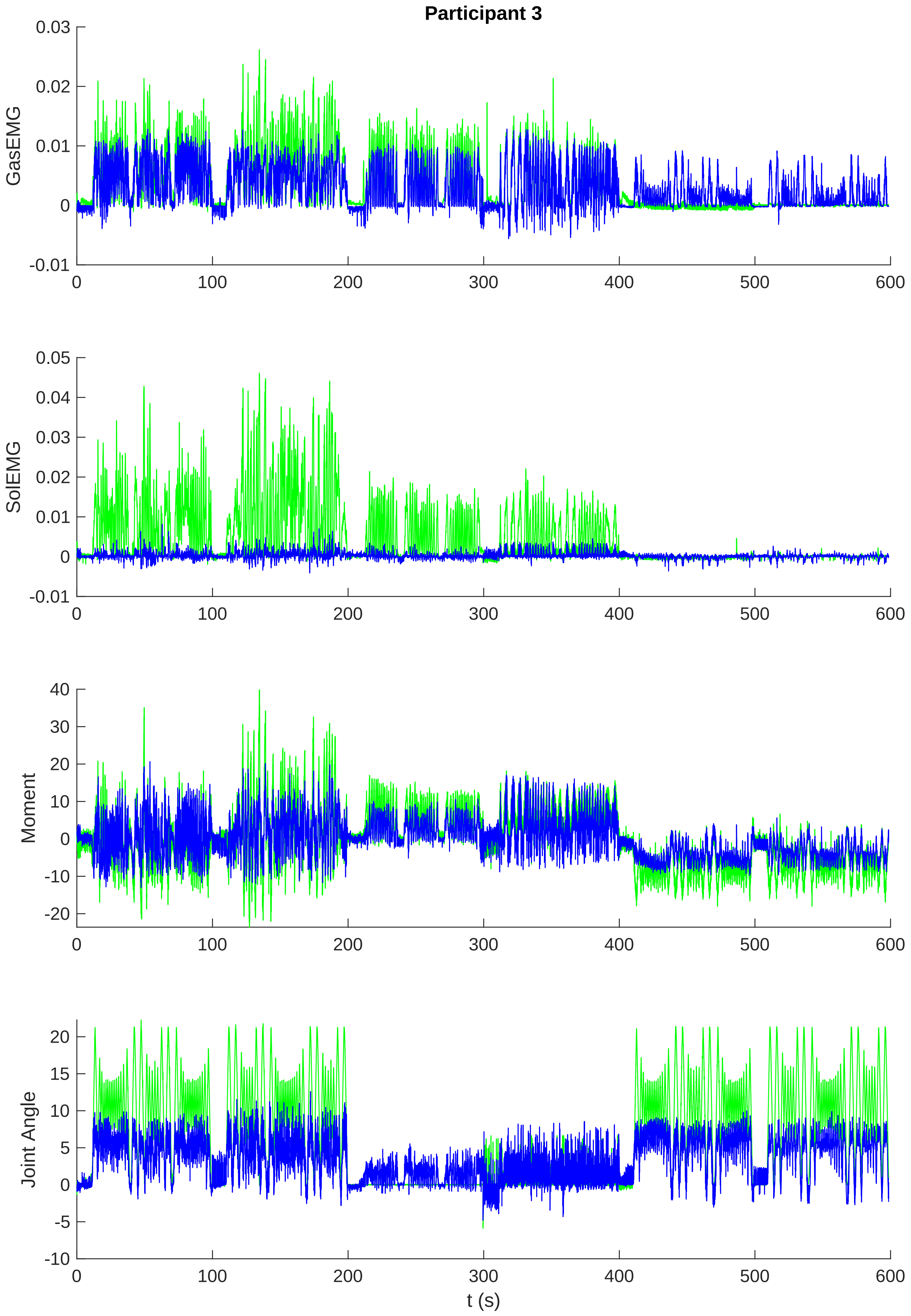
Participant 3
GasEMG
0.03
0.02
0.01
0
-0.01
SolEMG
0.05
0.04
0.03
0.02
0.01
0
-0.01
Moment
40
30
20
10
0
-10
-20
Joint Angle
20
15
10
5
0
-5
-10
0
100
200
300
400
500
600
t (s)

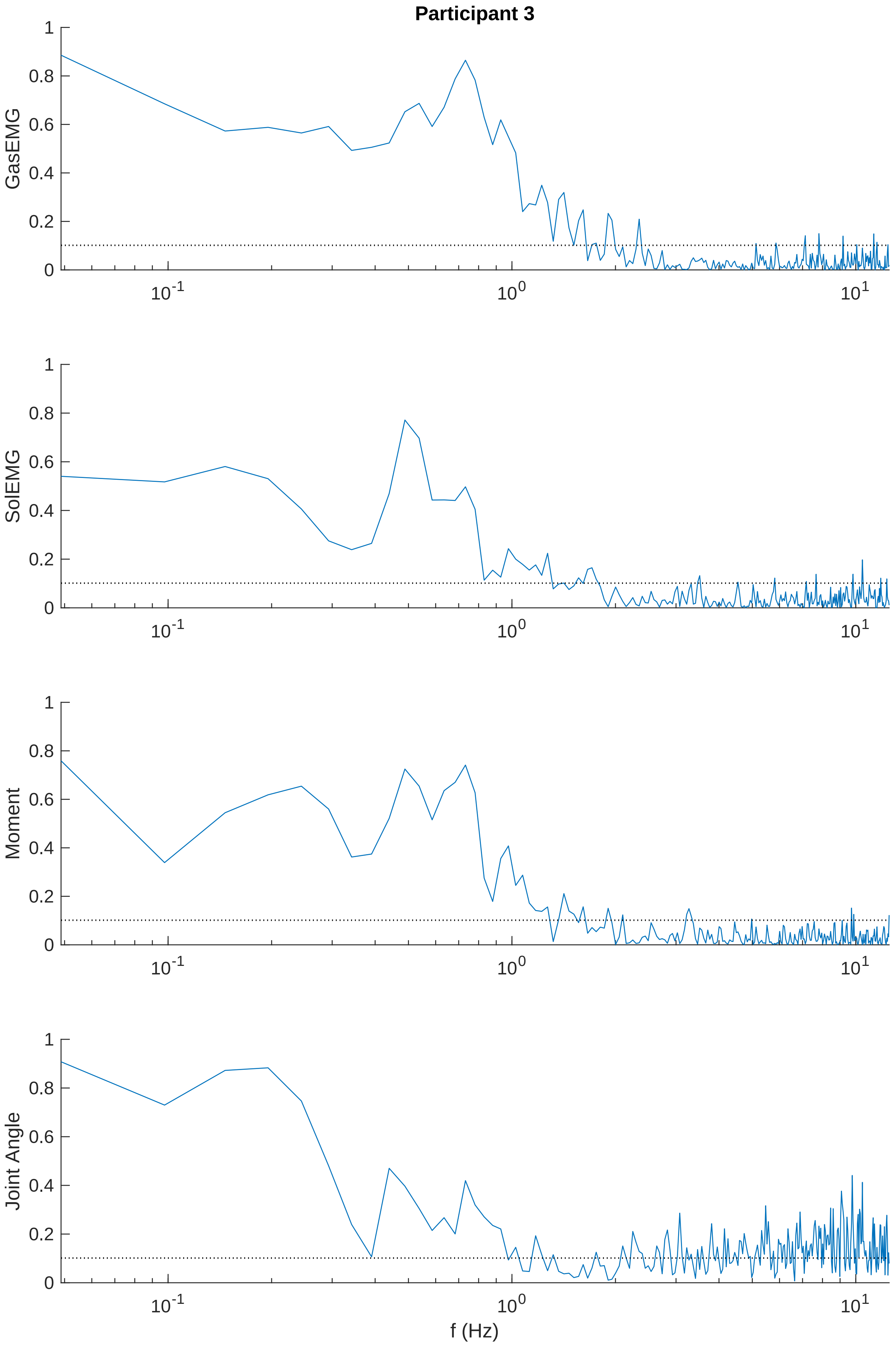

Participant 3
GasEMG
SolEMG
Moment
Joint Angle
1
0.8
0.6
0.4
0.2
0
10^{-1}
10^{0}
10^{1}
f (Hz)

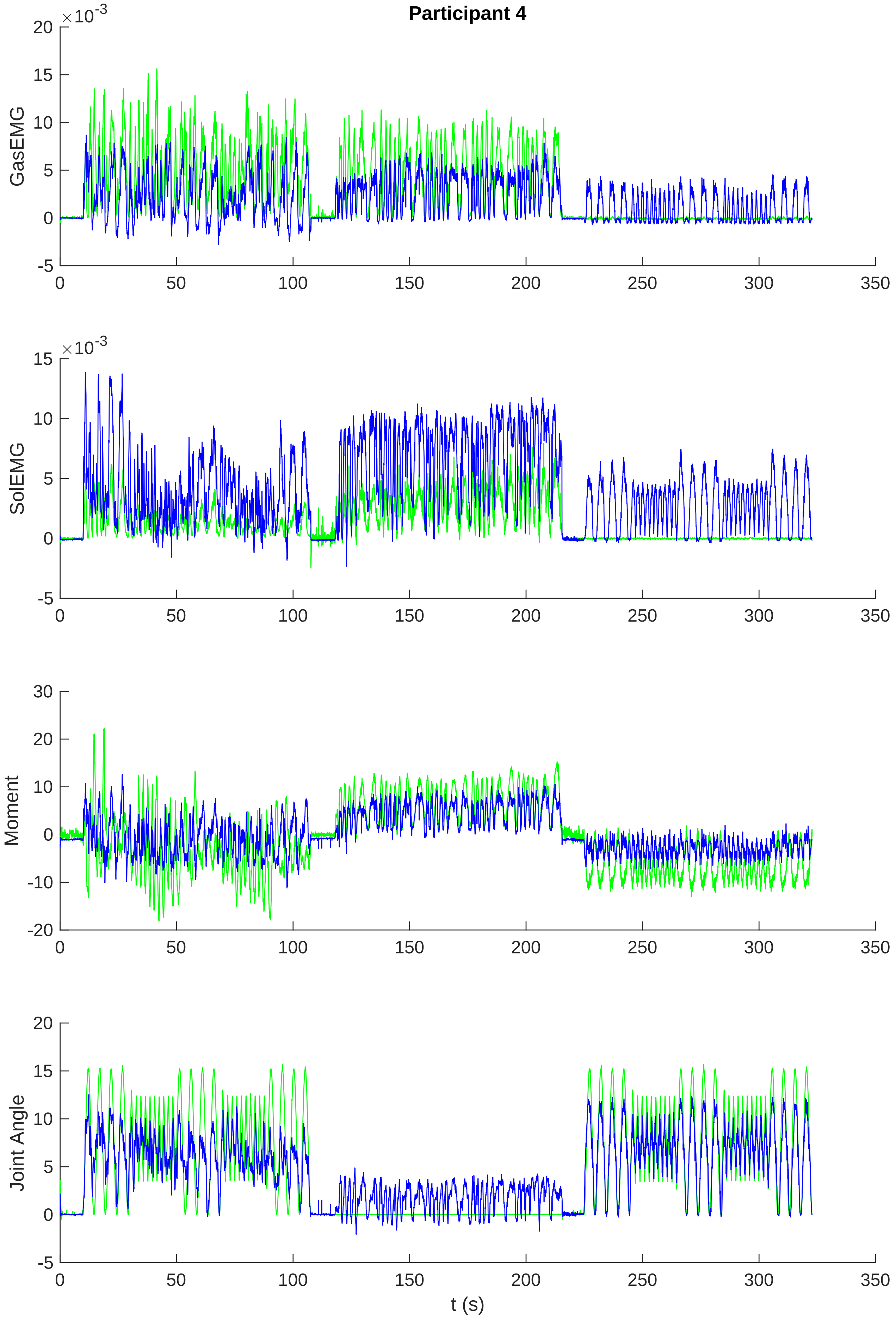
Participant 4
GasEMG
×10^-3
SolEMG
×10^-3
Moment
Joint Angle
t (s)

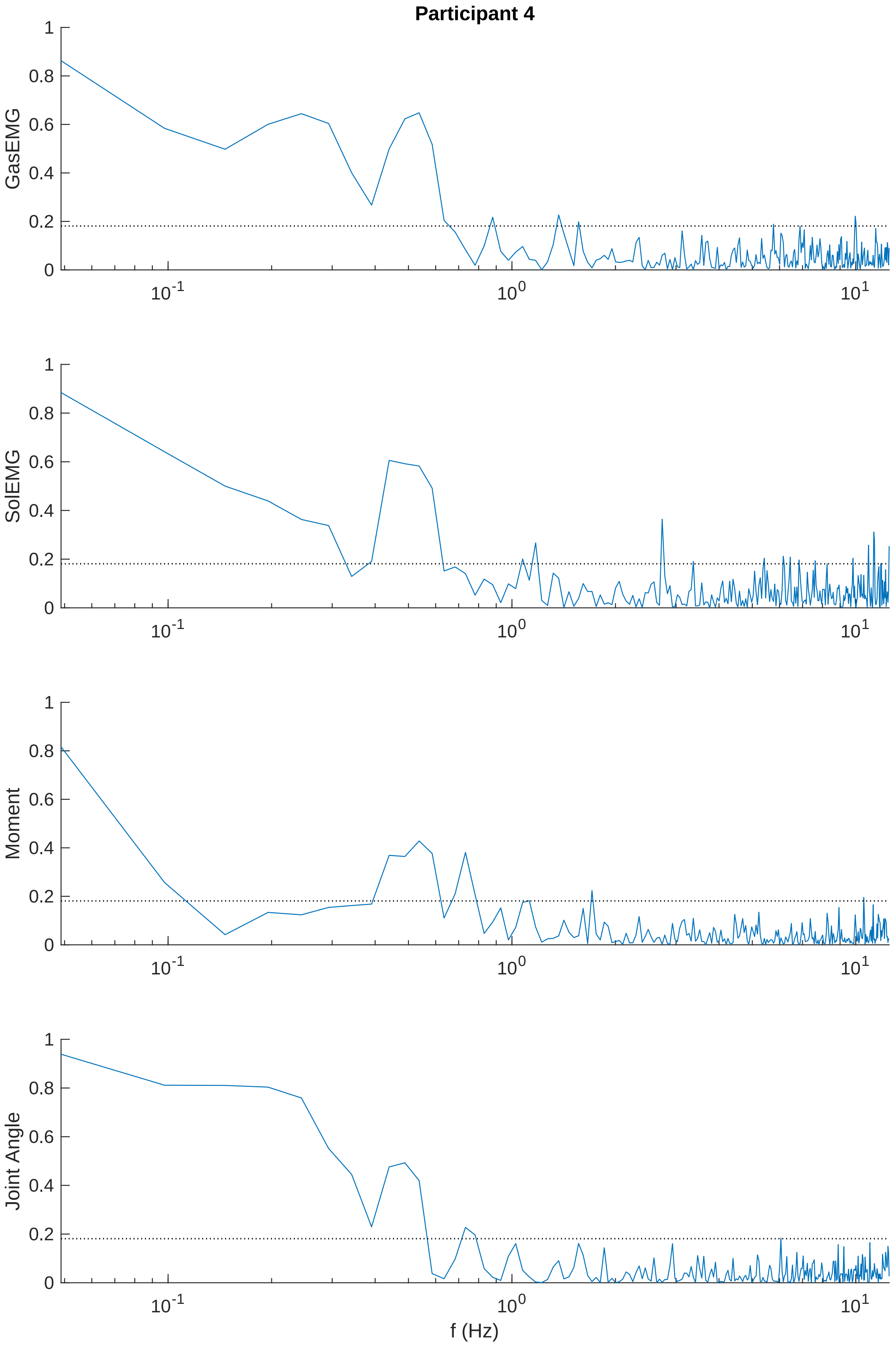
Participant 4
GasEMG
SolEMG
Moment
Joint Angle
f (Hz)
0
0.2
0.4
0.6
0.8
1
10^{-1}
10^{0}
10^{1}

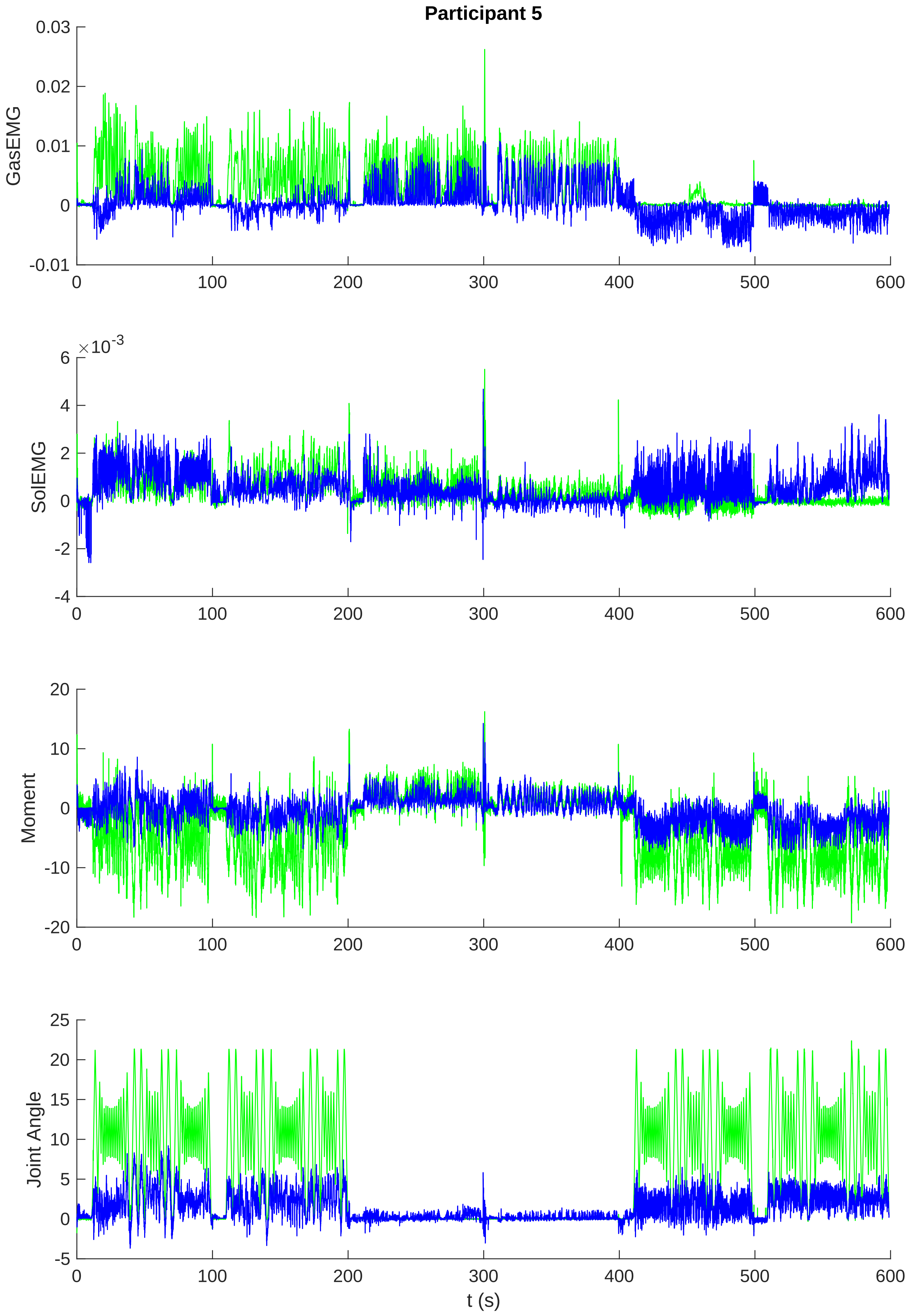

Participant 5
GasEMG
0.03
0.02
0.01
0
-0.01
0
100
200
300
400
500
600
×10-3
SolEMG
6
4
2
0
-2
-4
Moment
20
10
0
-10
-20
Joint Angle
25
20
15
10
5
0
-5
t (s)

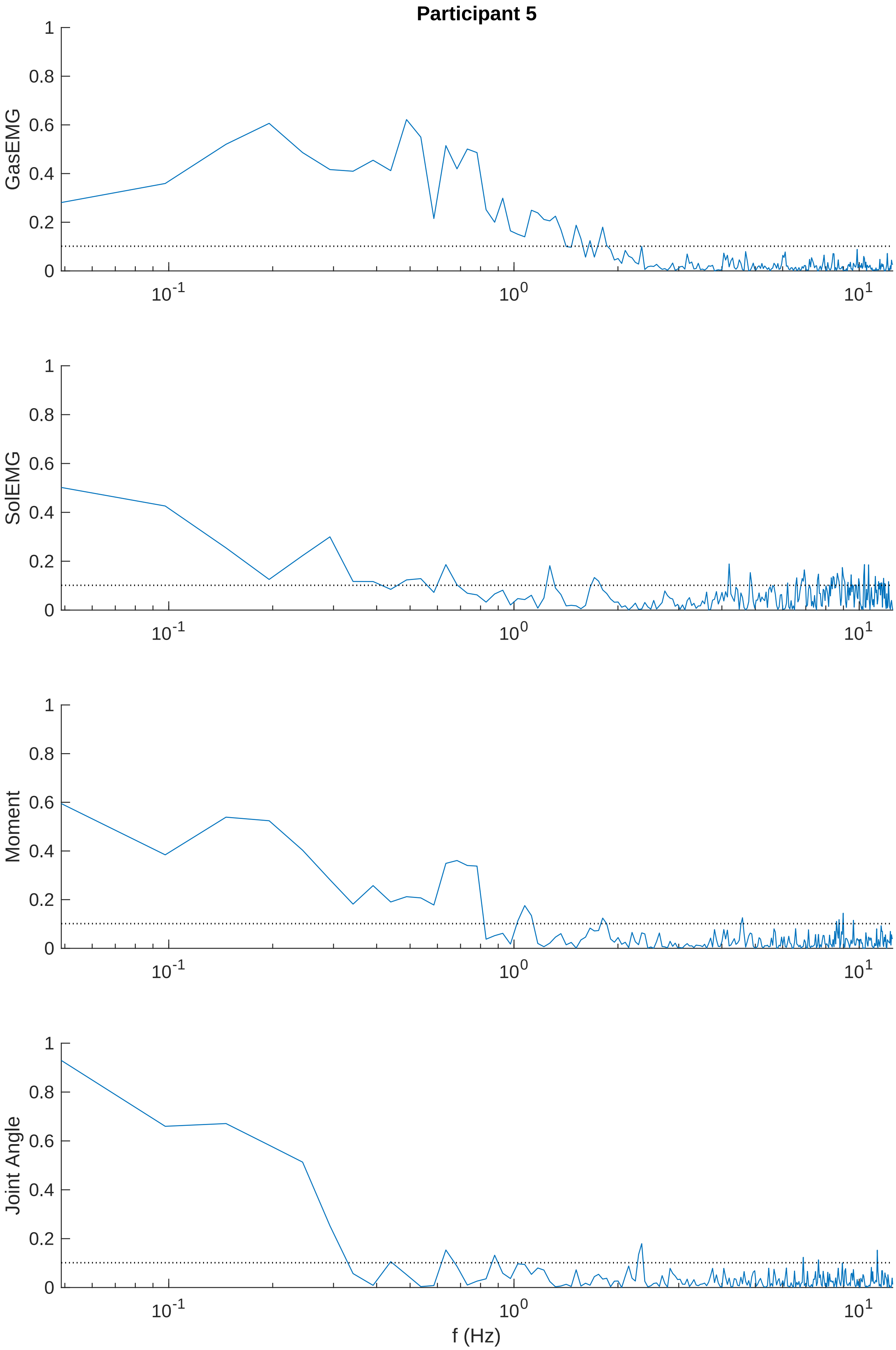
Participant 5
GasEMG
SolEMG
Moment
Joint Angle
f (Hz)
0
0.2
0.4
0.6
0.8
1
10^-1
10^0
10^1

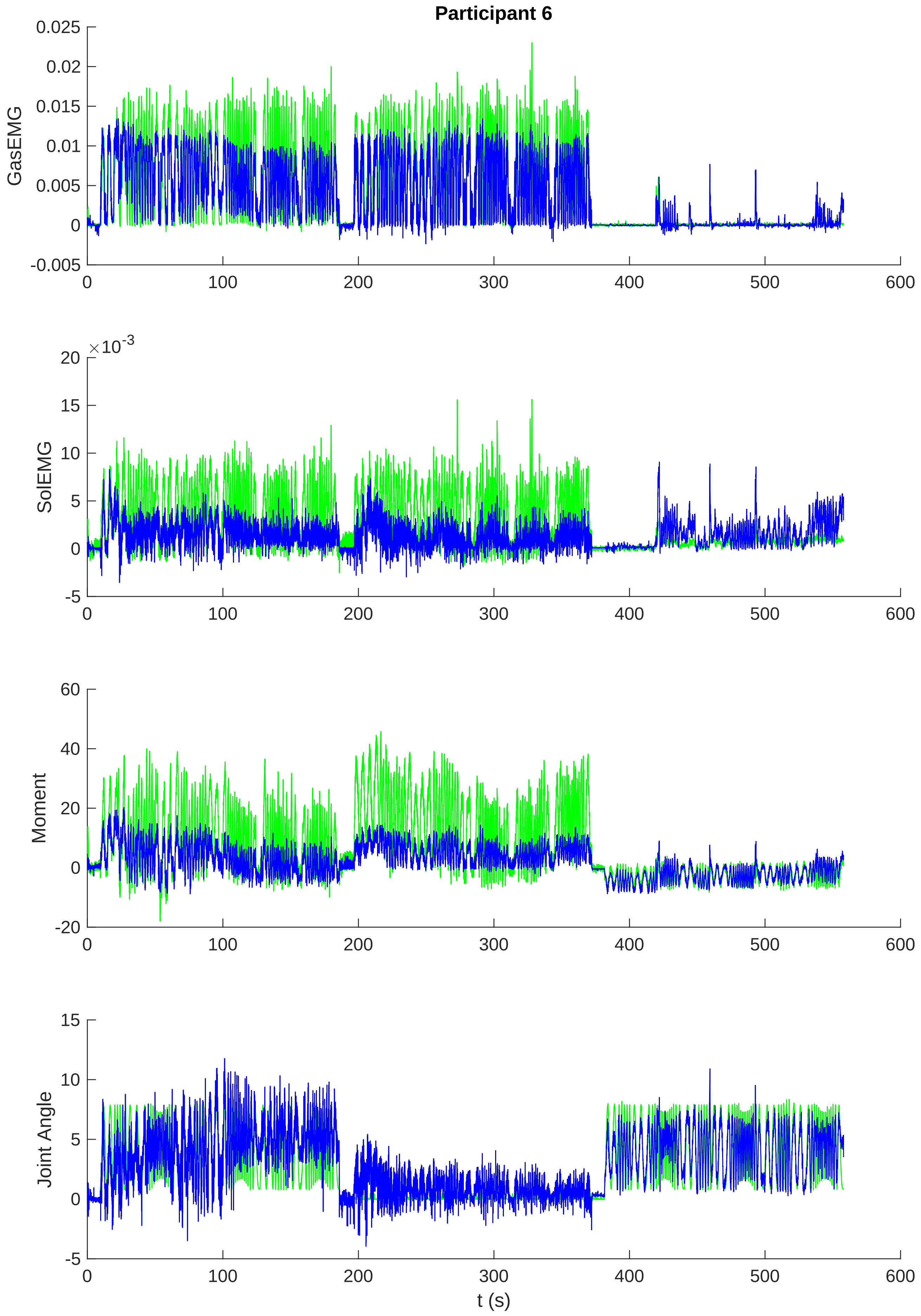

Participant 6
GasEMG
SolEMG
$\times 10^{-3}$
Moment
Joint Angle
t (s)

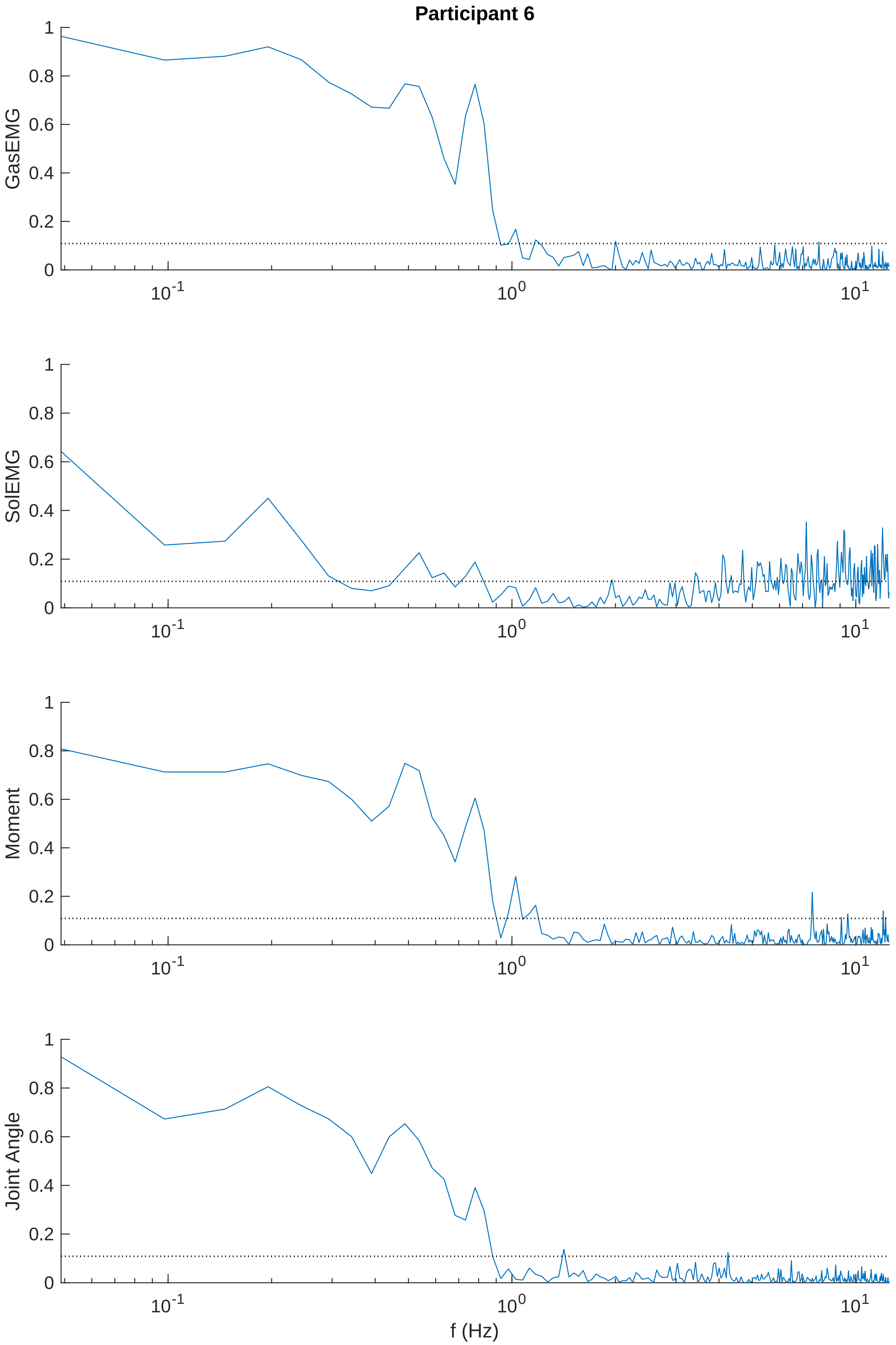
Participant 6
GasEMG
SolEMG
Moment
Joint Angle
0
0.2
0.4
0.6
0.8
1
10^{-1}
10^{0}
10^{1}
f (Hz)

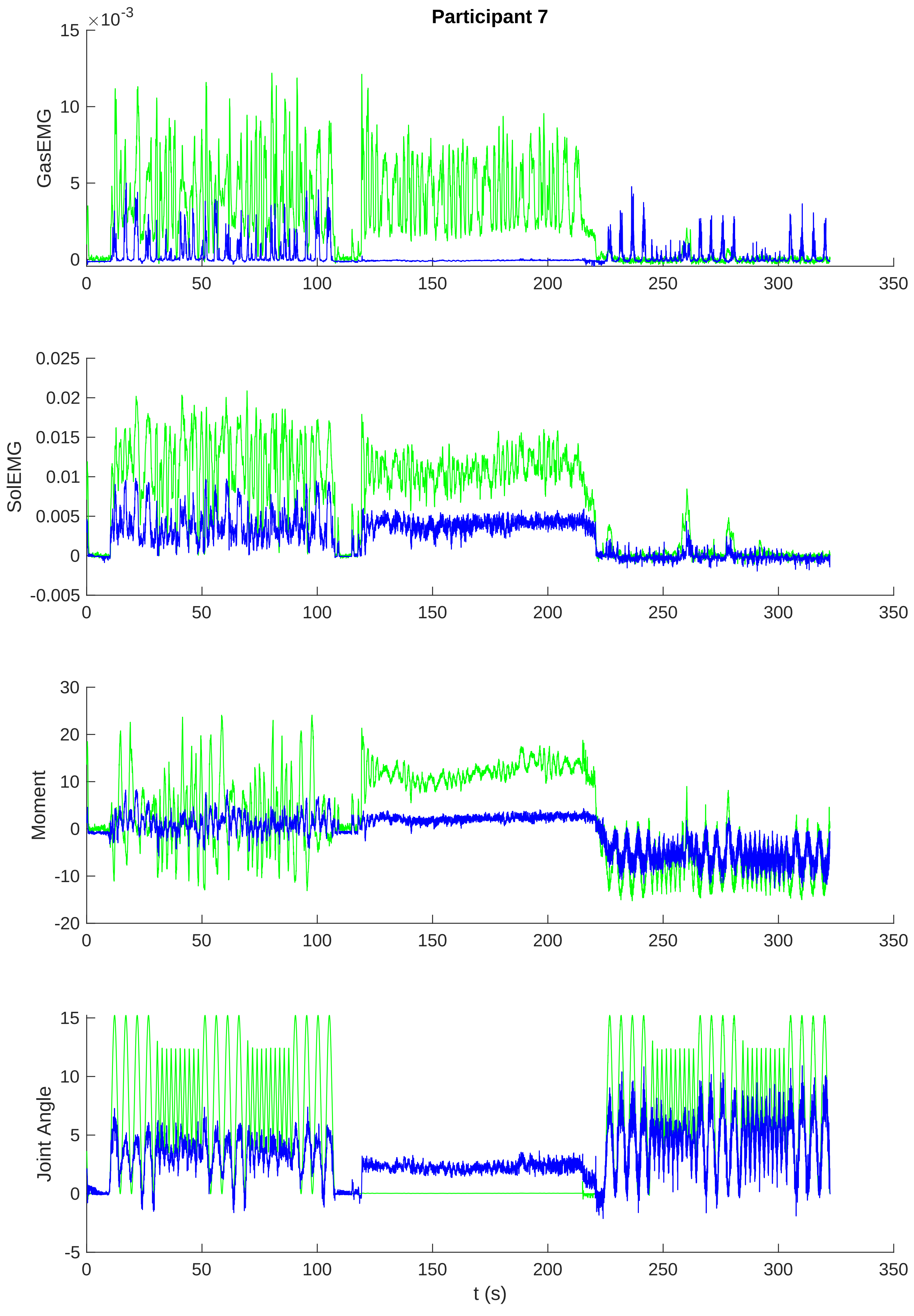
Participant 7
×10^-3
GasEMG
SolEMG
Moment
Joint Angle
t (s)

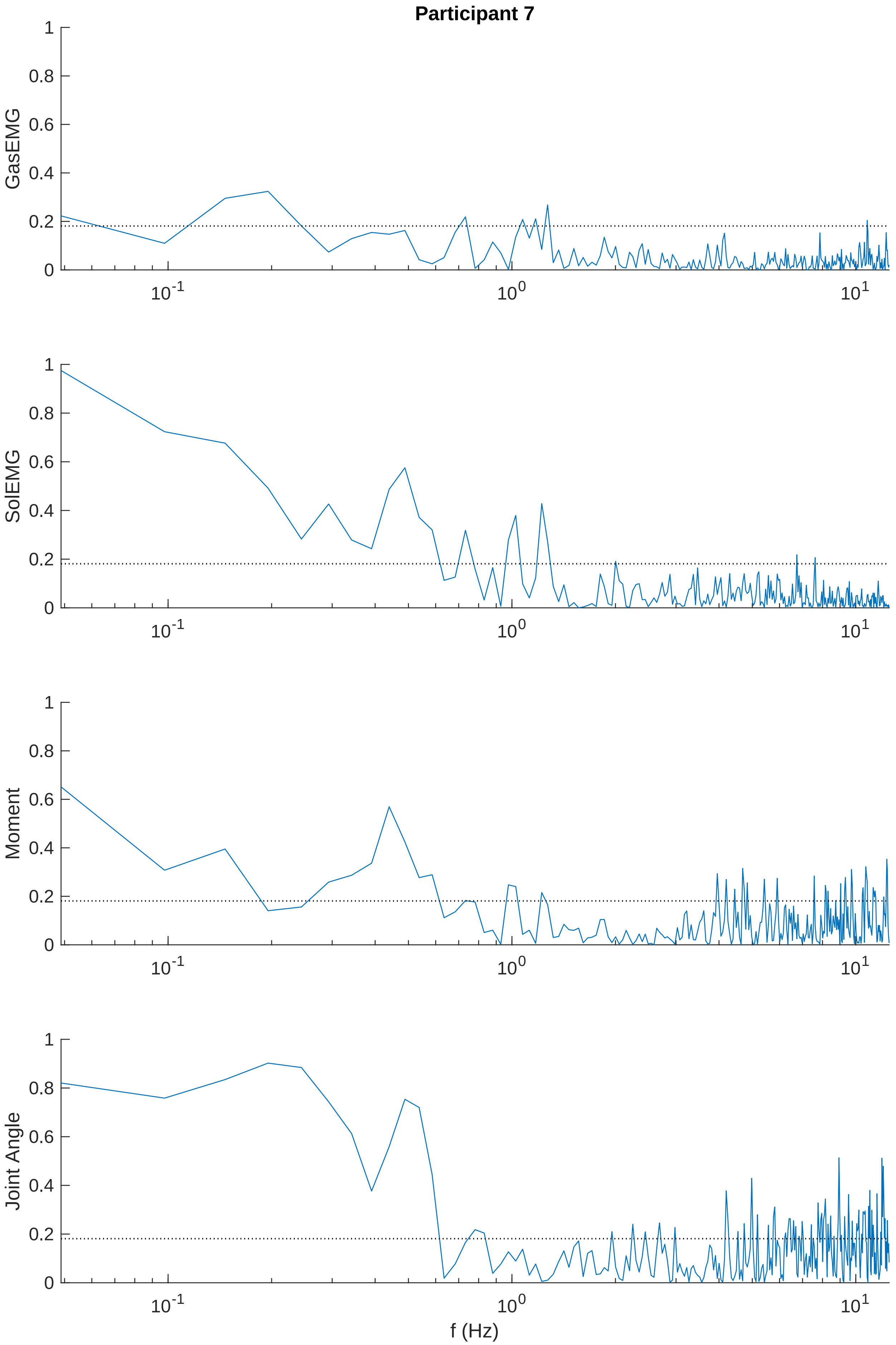

Participant 7
GasEMG
SolEMG
Moment
Joint Angle
1
0.8
0.6
0.4
0.2
0
10^{-1}
10^{0}
10^{1}
f (Hz)

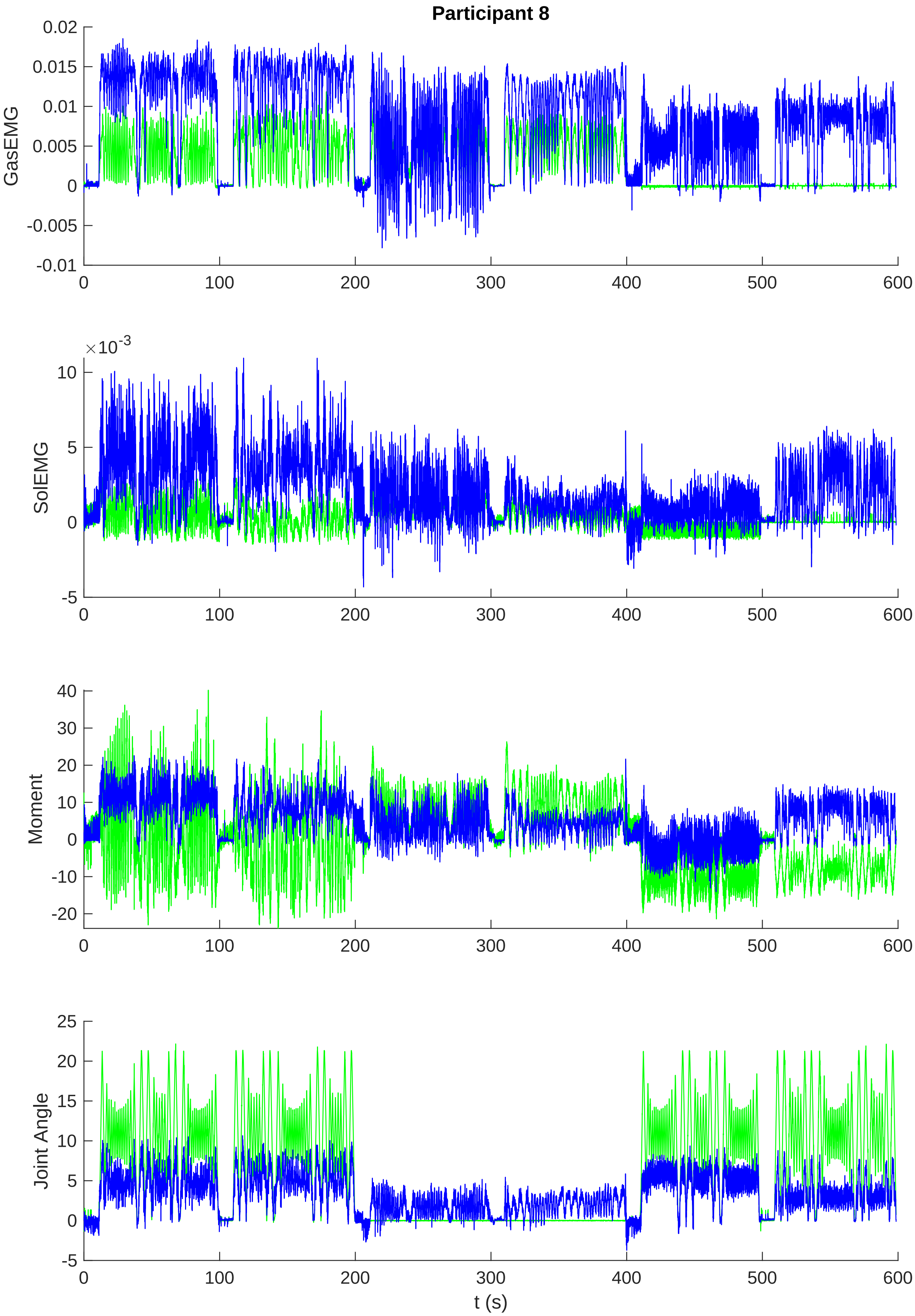
Participant 8
GasEMG
0.02
0.015
0.01
0.005
0
-0.005
-0.01
SolEMG
×10-3
10
5
0
-5
Moment
40
30
20
10
0
-10
-20
Joint Angle
25
20
15
10
5
0
-5
0
100
200
300
400
500
600
t (s)

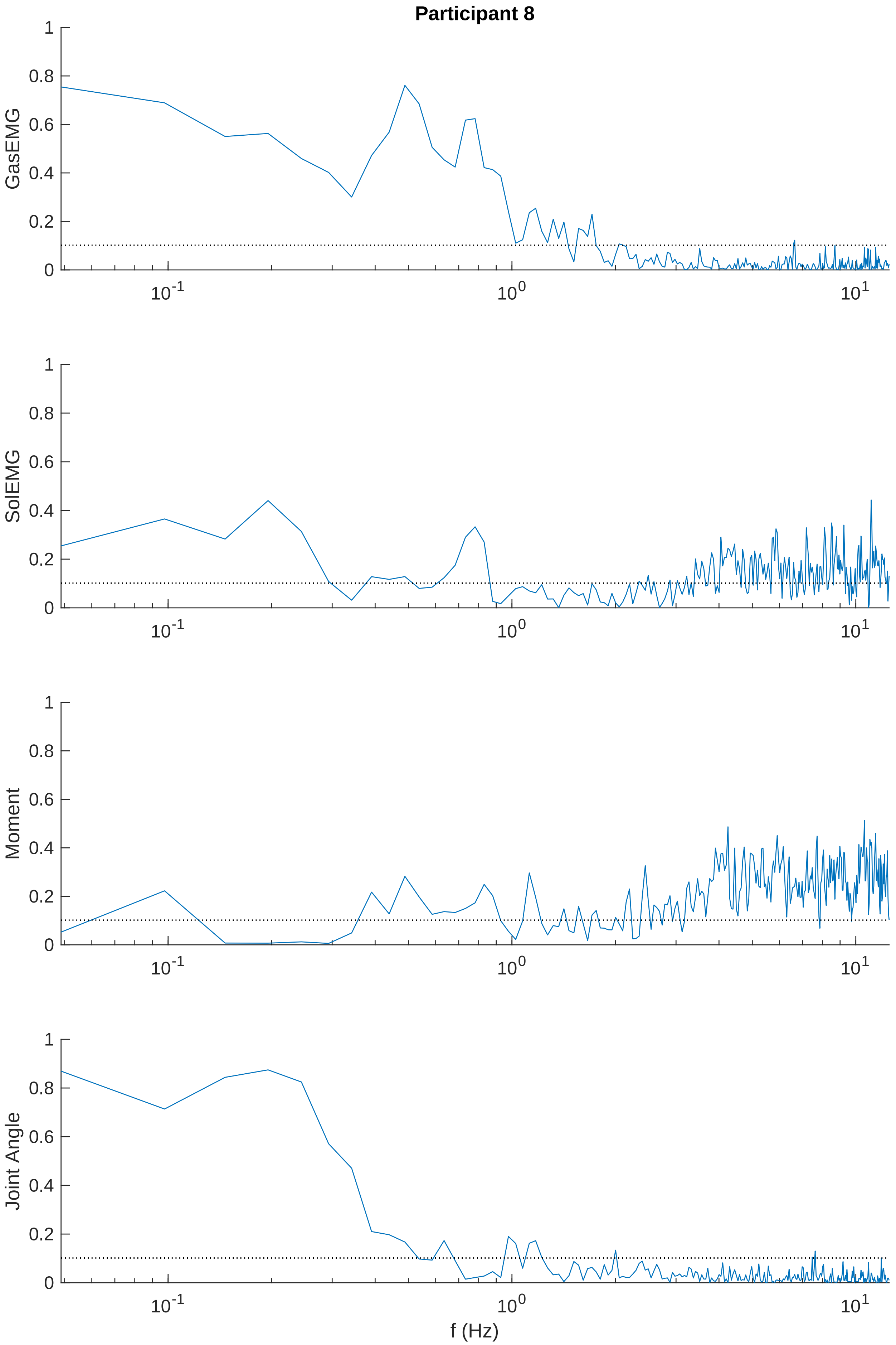
Participant 8
GasEMG
SolEMG
Moment
Joint Angle
f (Hz)
1
0.8
0.6
0.4
0.2
0
10^{-1}
10^{0}
10^{1}

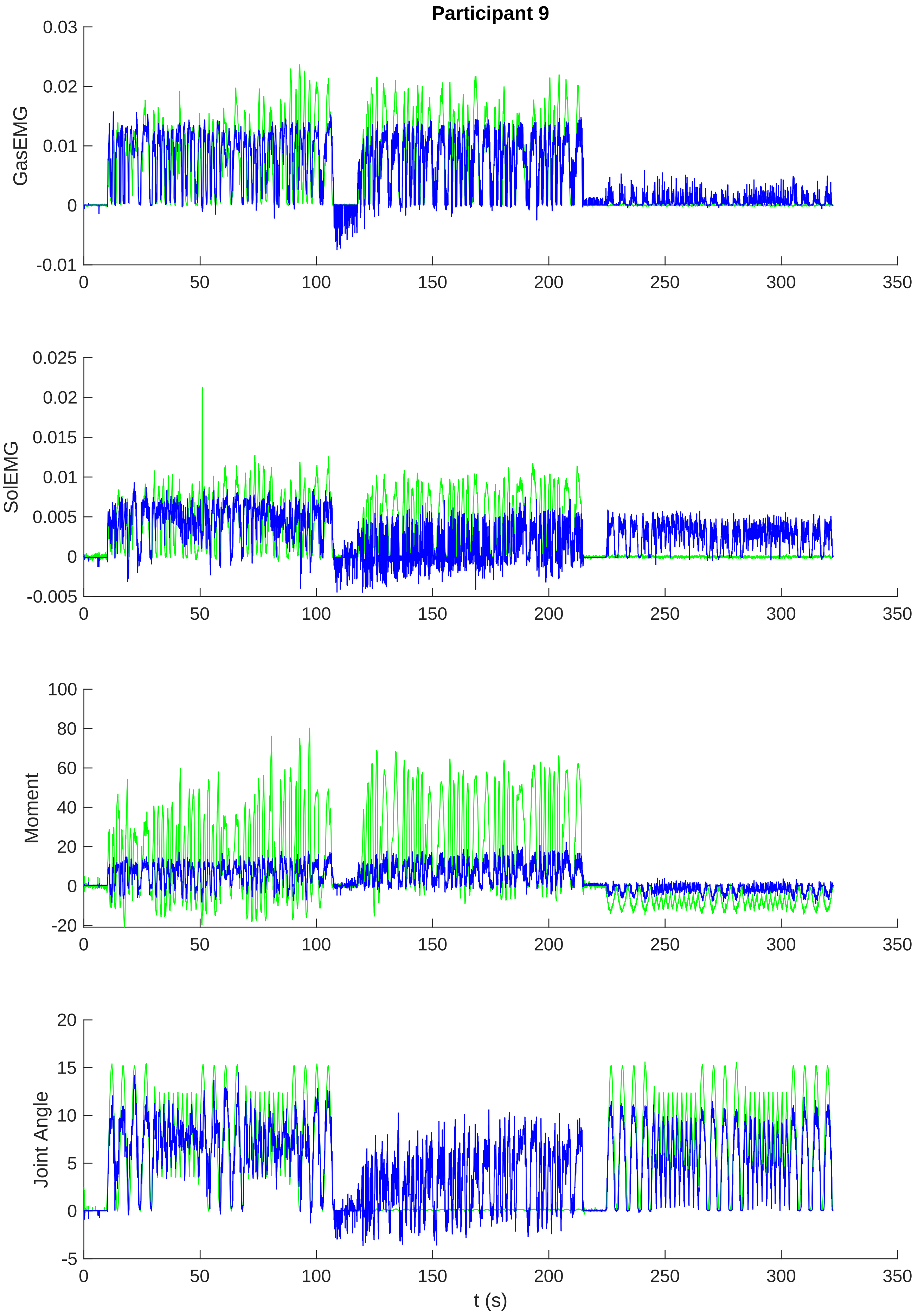

Participant 9
GasEMG
SolEMG
Moment
Joint Angle
t (s)

Participant 9

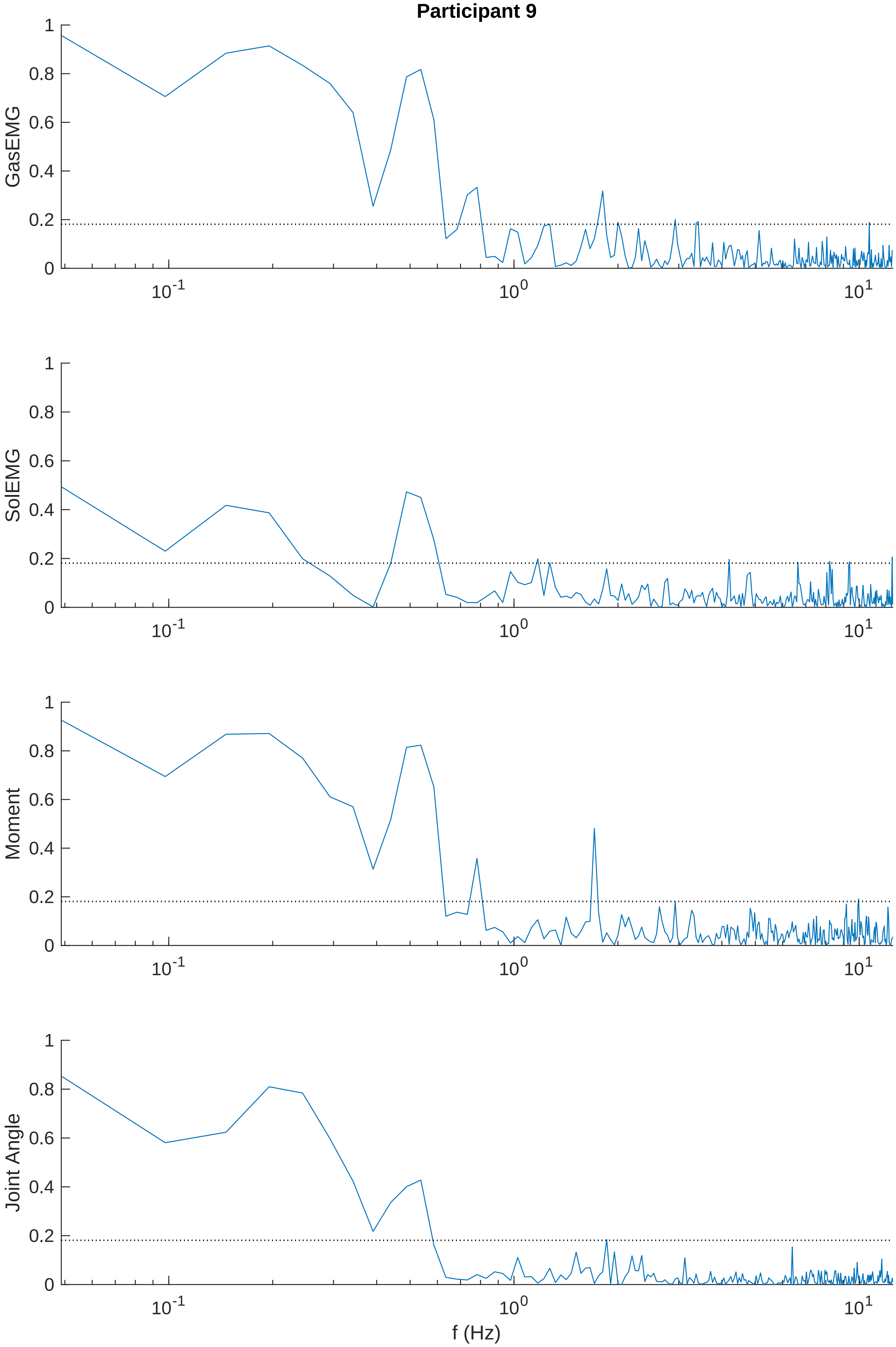

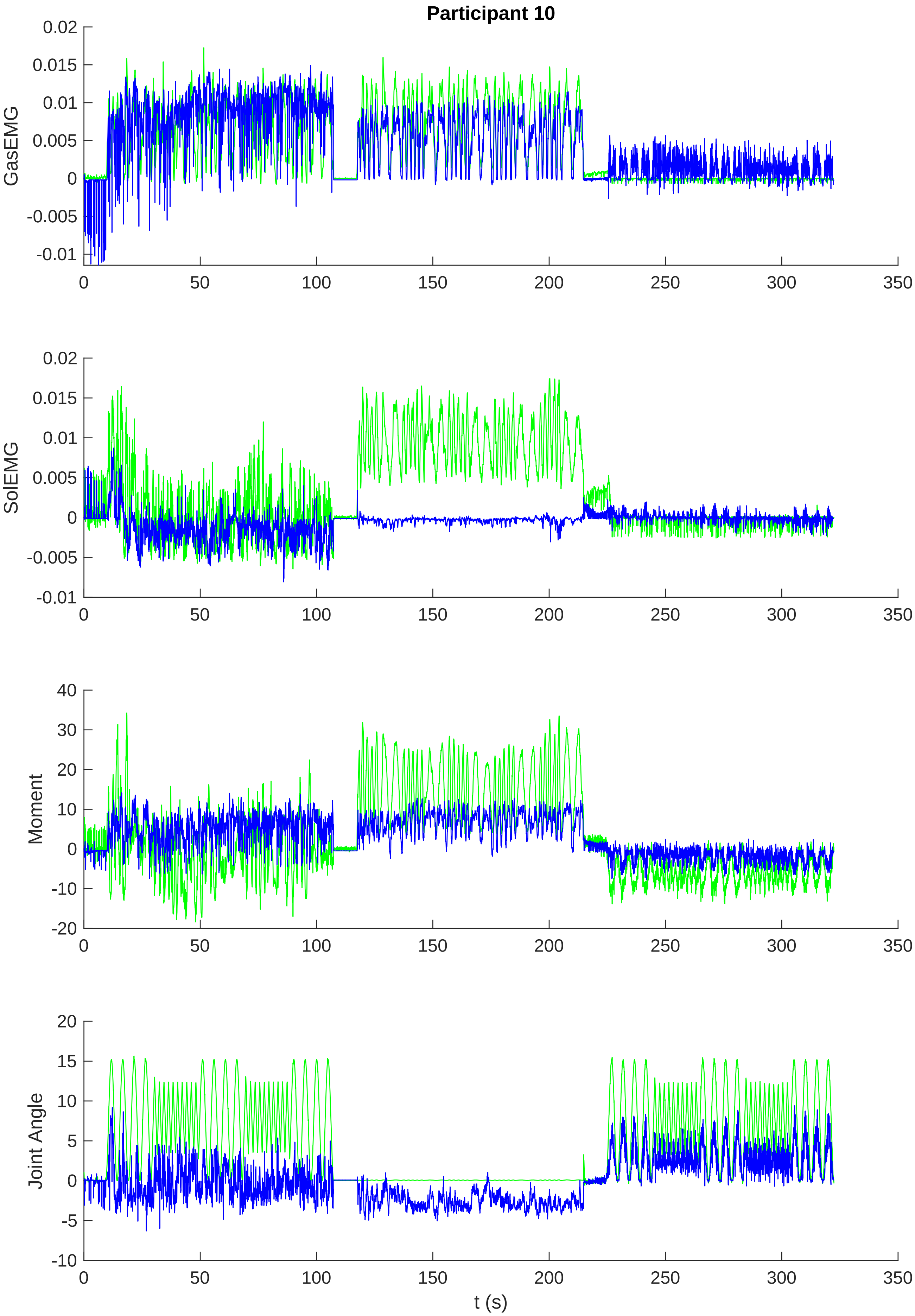
Participant 10
GasEMG
SolEMG
Moment
Joint Angle
t (s)

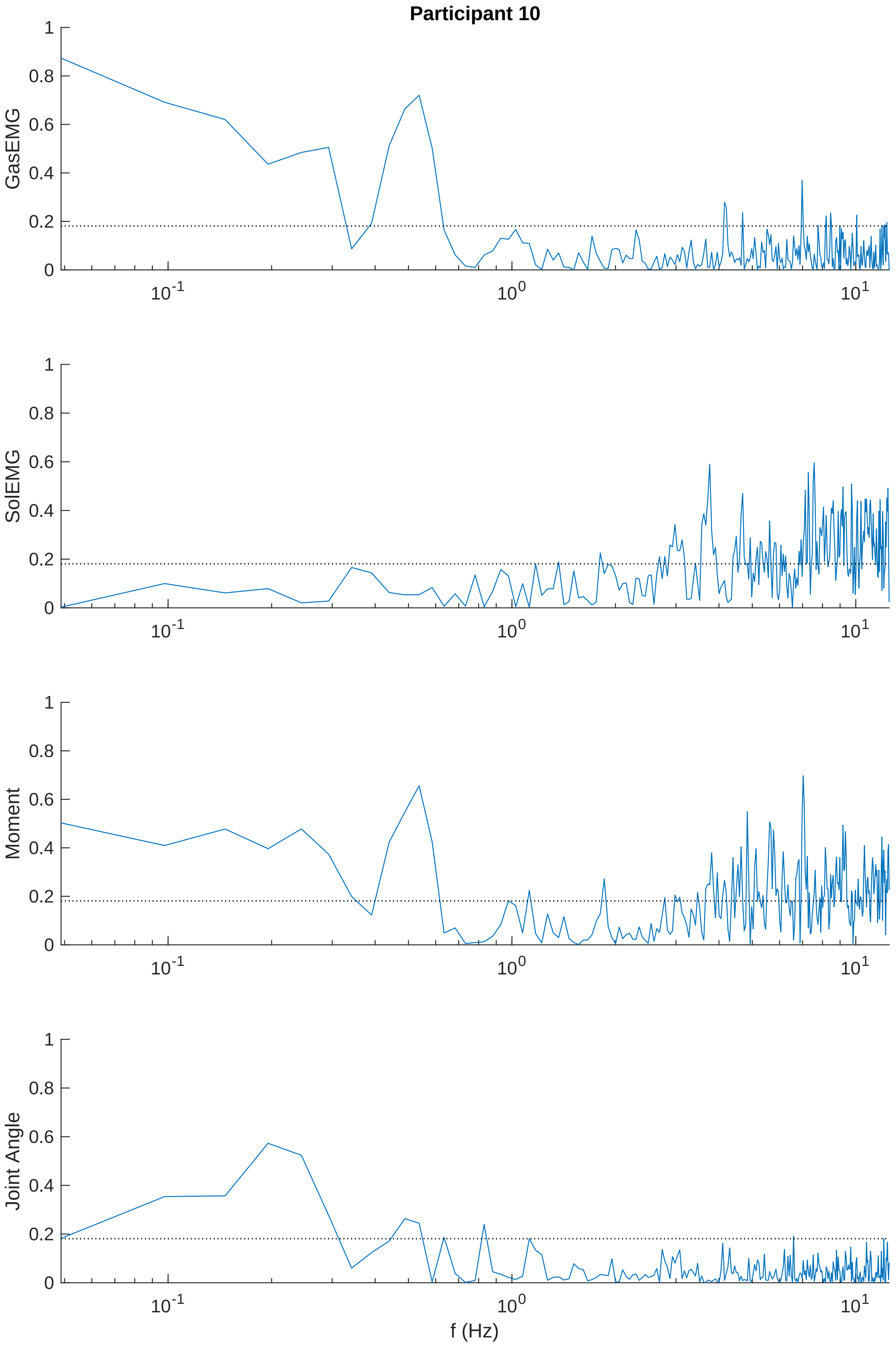
Participant 10
GasEMG
SolEMG
Moment
Joint Angle
1
0.8
0.6
0.4
0.2
0
10^{-1}
10^{0}
10^{1}
f (Hz)

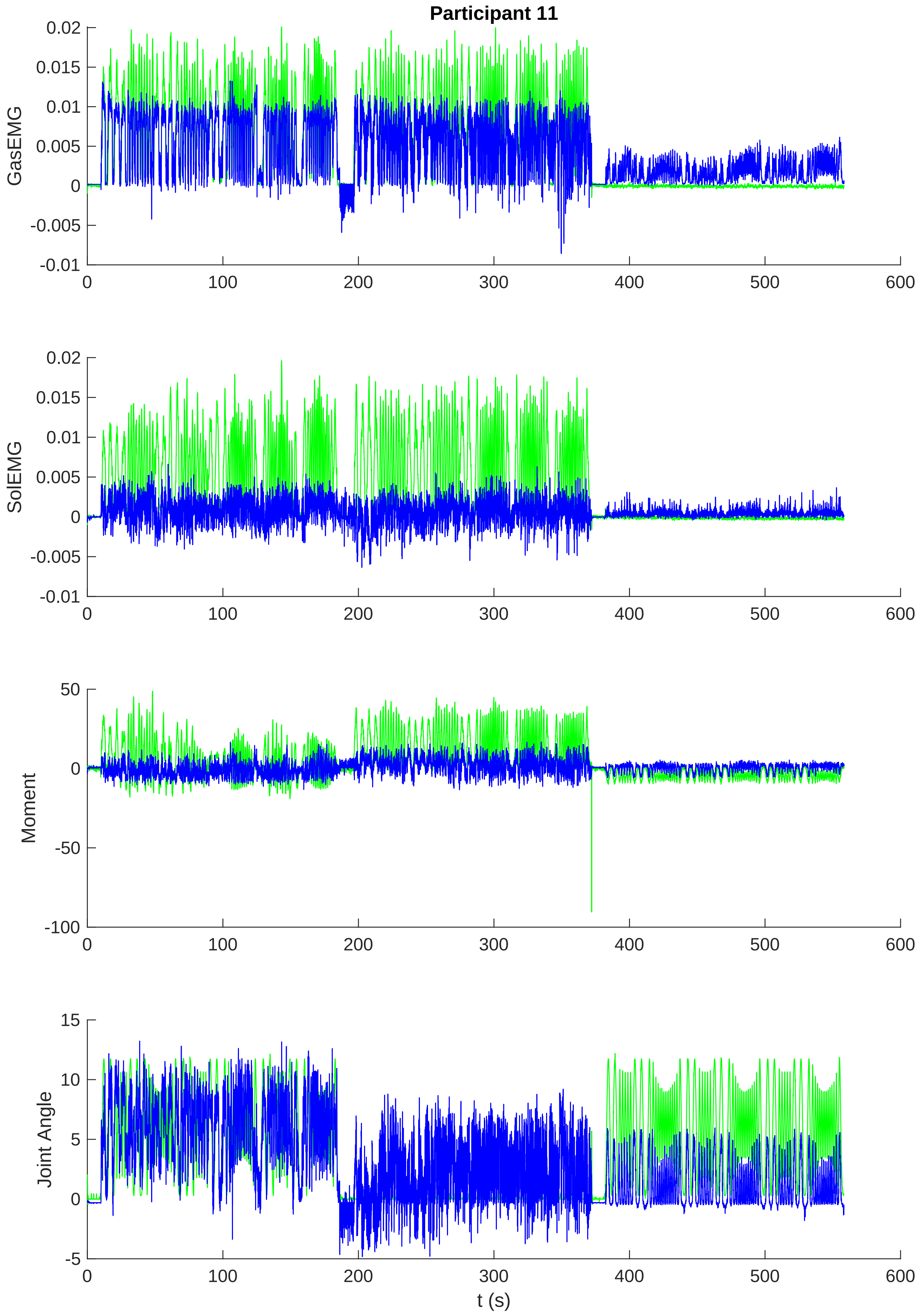

Participant 11
GasEMG
SolEMG
Moment
Joint Angle
t (s)

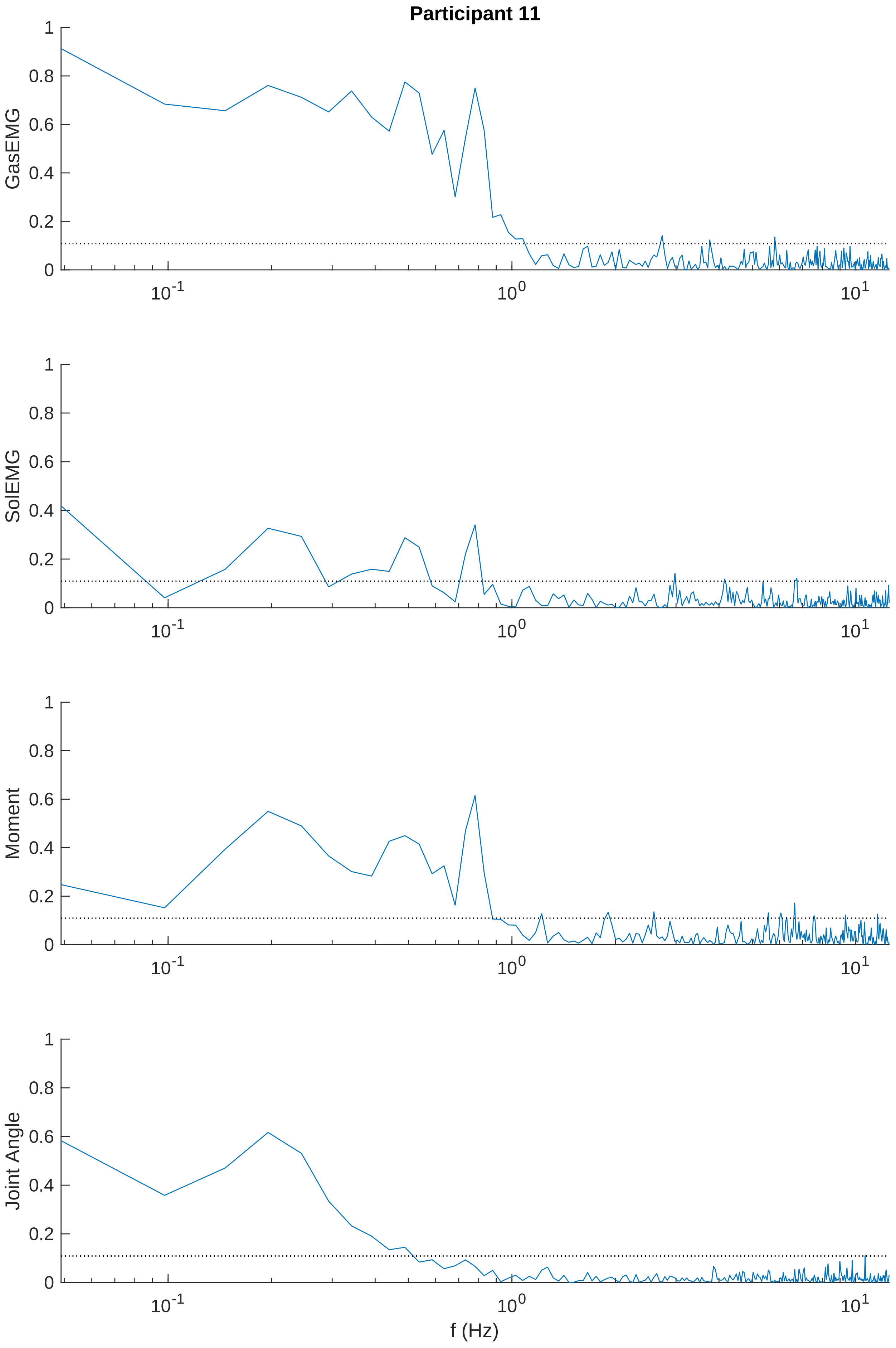
Participant 11
GasEMG
SolEMG
Moment
Joint Angle
0
0.2
0.4
0.6
0.8
1
10^{-1}
10^{0}
10^{1}
f (Hz)

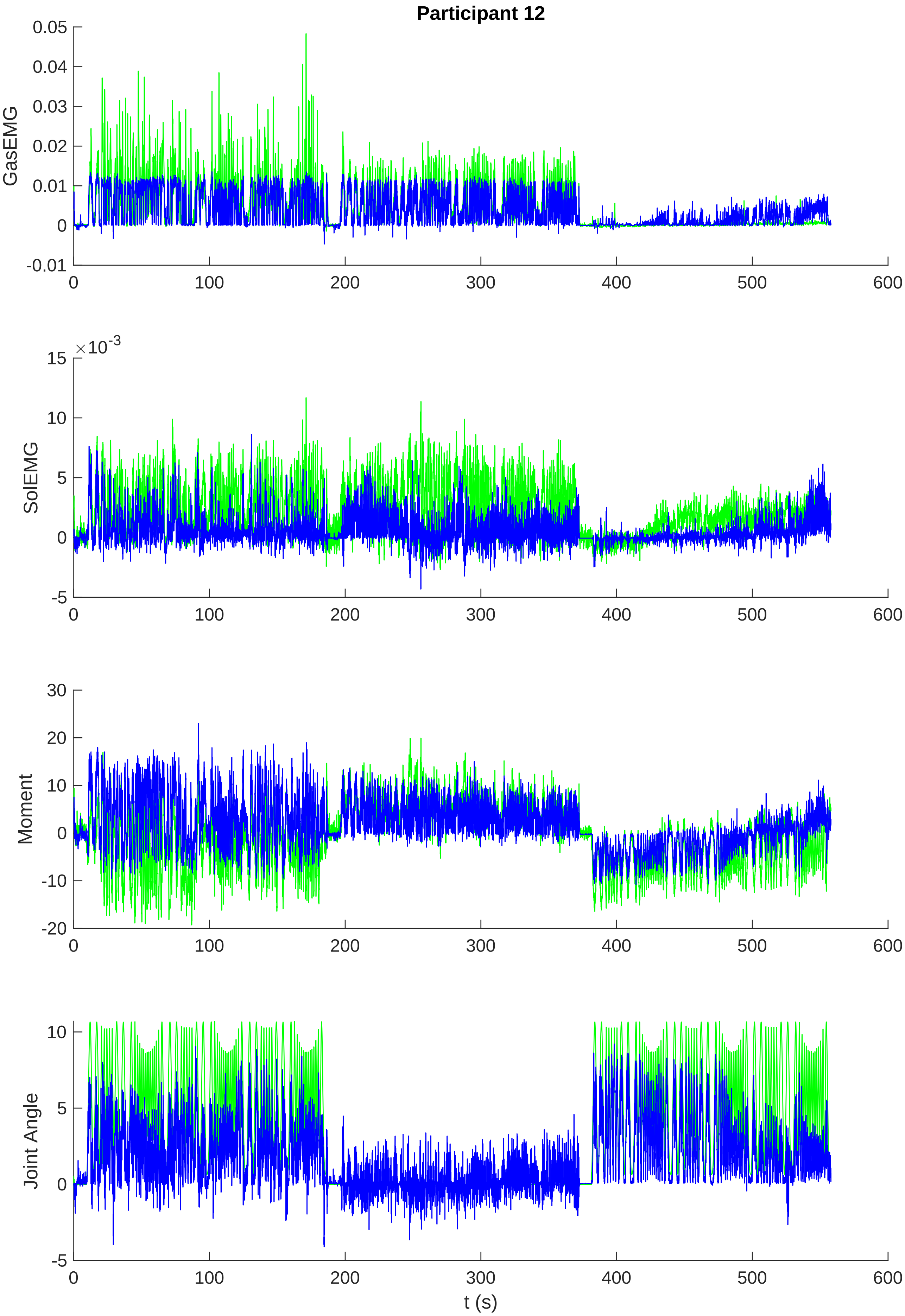

Participant 12
GasEMG
0.05
0.04
0.03
0.02
0.01
0
-0.01
SolEMG
×10^-3
15
10
5
0
-5
Moment
30
20
10
0
-10
-20
Joint Angle
10
5
0
-5
0
100
200
300
400
500
600
t (s)

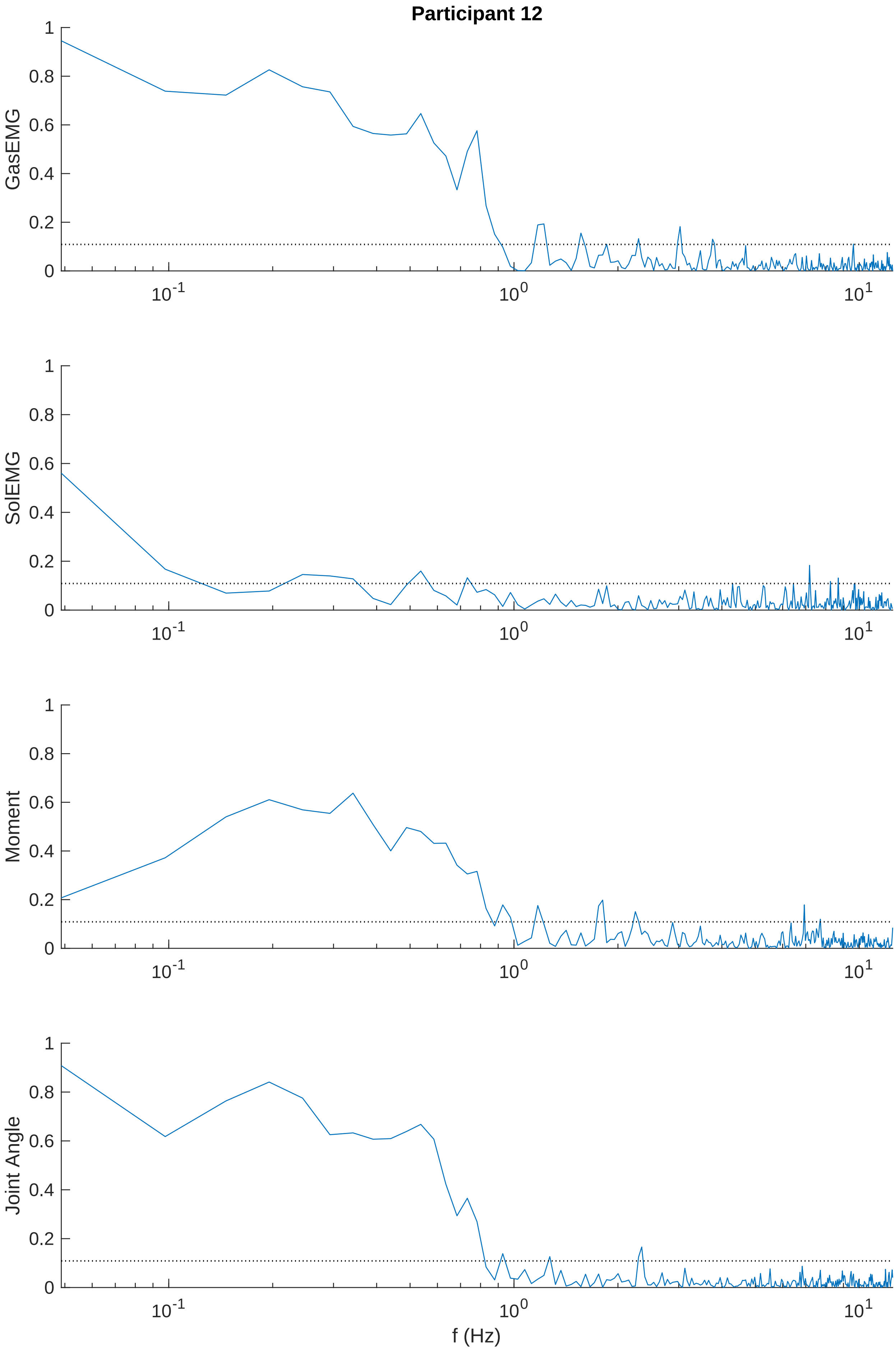

Participant 12
GasEMG
SolEMG
Moment
Joint Angle
f (Hz)
1
0.8
0.6
0.4
0.2
0
10^{-1}
10^{0}
10^{1}

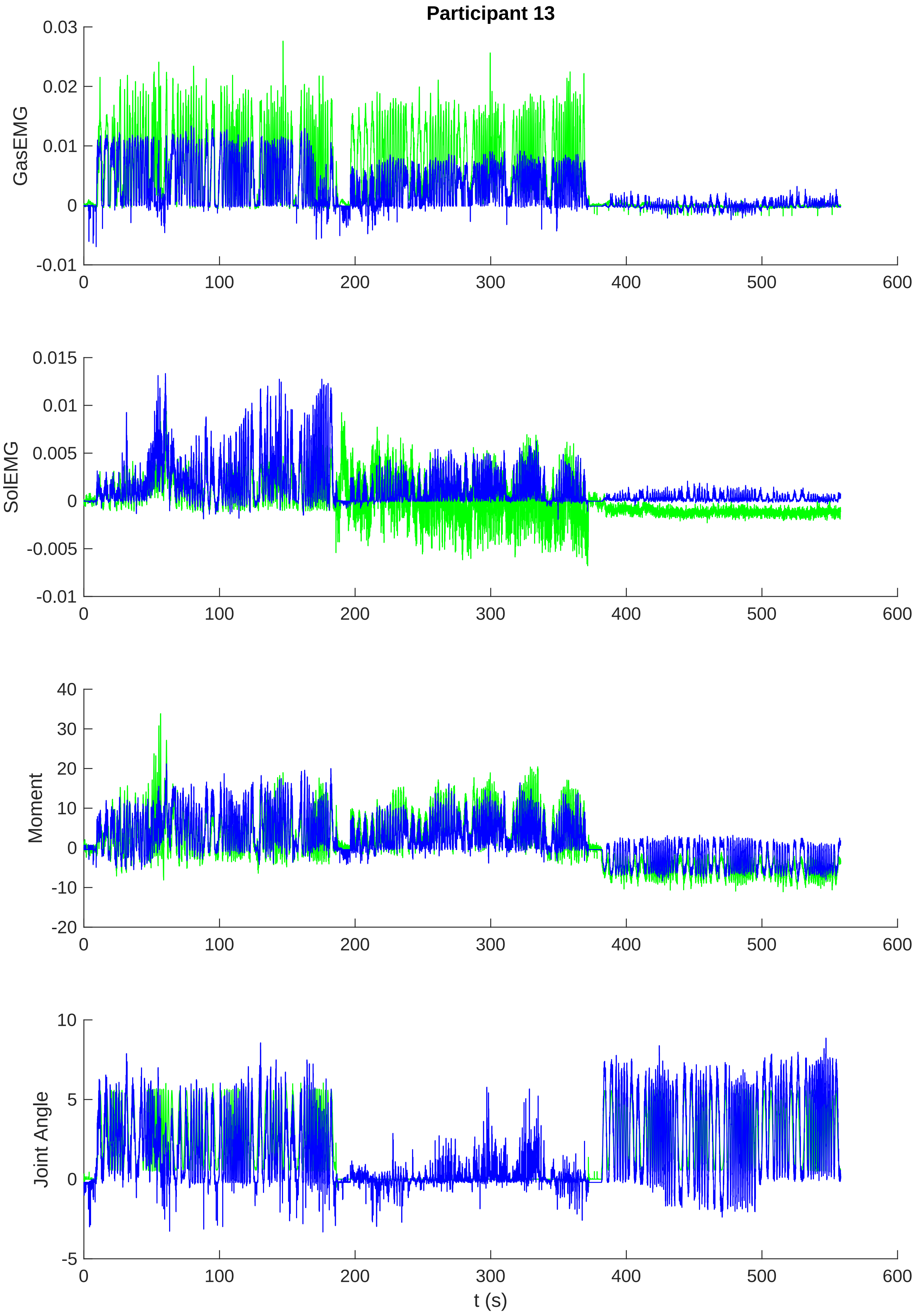
Participant 13
GasEMG
0.03
0.02
0.01
0
-0.01
SolEMG
0.015
0.01
0.005
0
-0.005
-0.01
Moment
40
30
20
10
0
-10
-20
Joint Angle
10
5
0
-5
0
100
200
300
400
500
600
t (s)

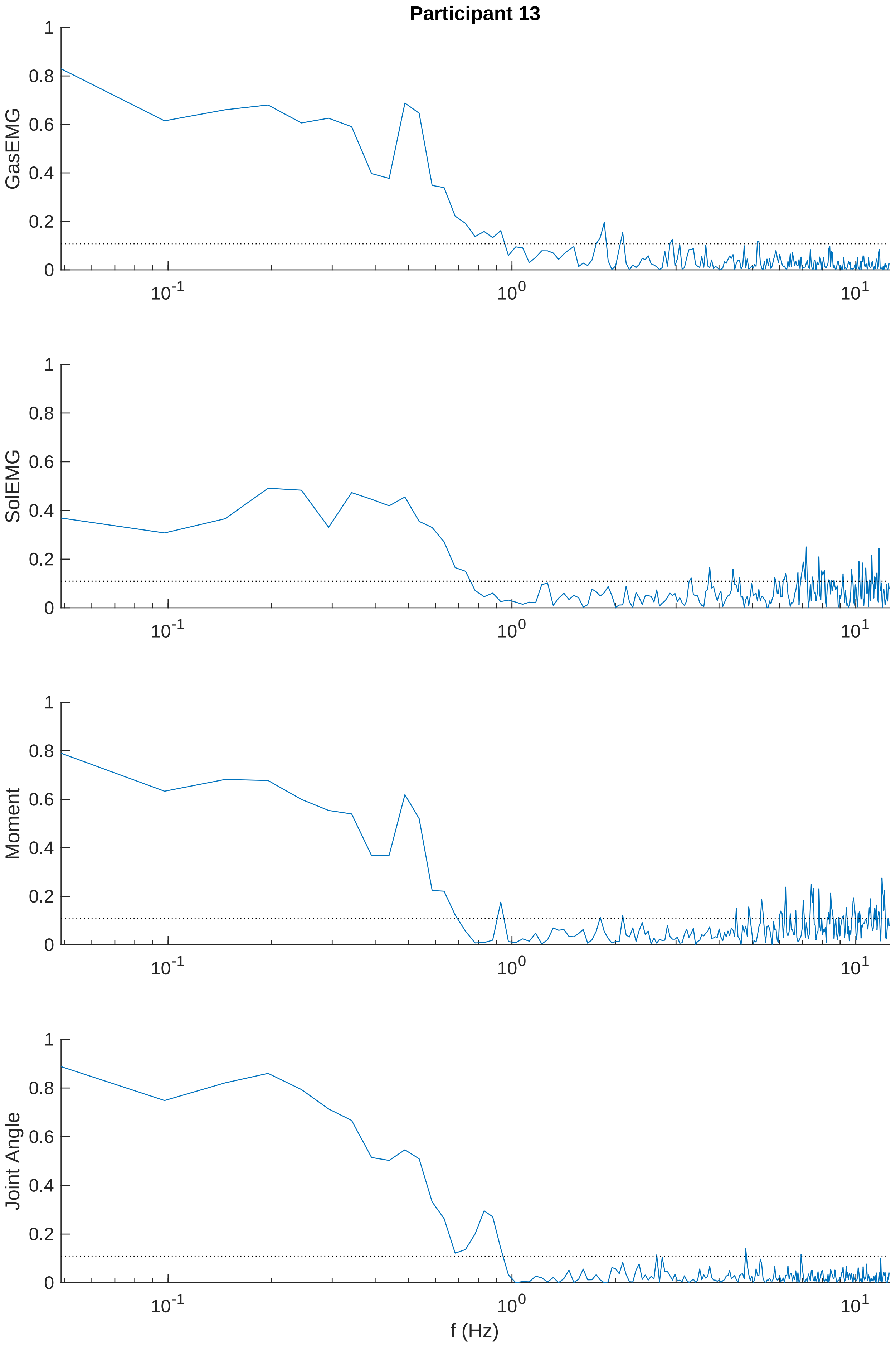

Participant 13
GasEMG
SolEMG
Moment
Joint Angle
1
0.8
0.6
0.4
0.2
0
10^{-1}
10^{0}
10^{1}
f (Hz)

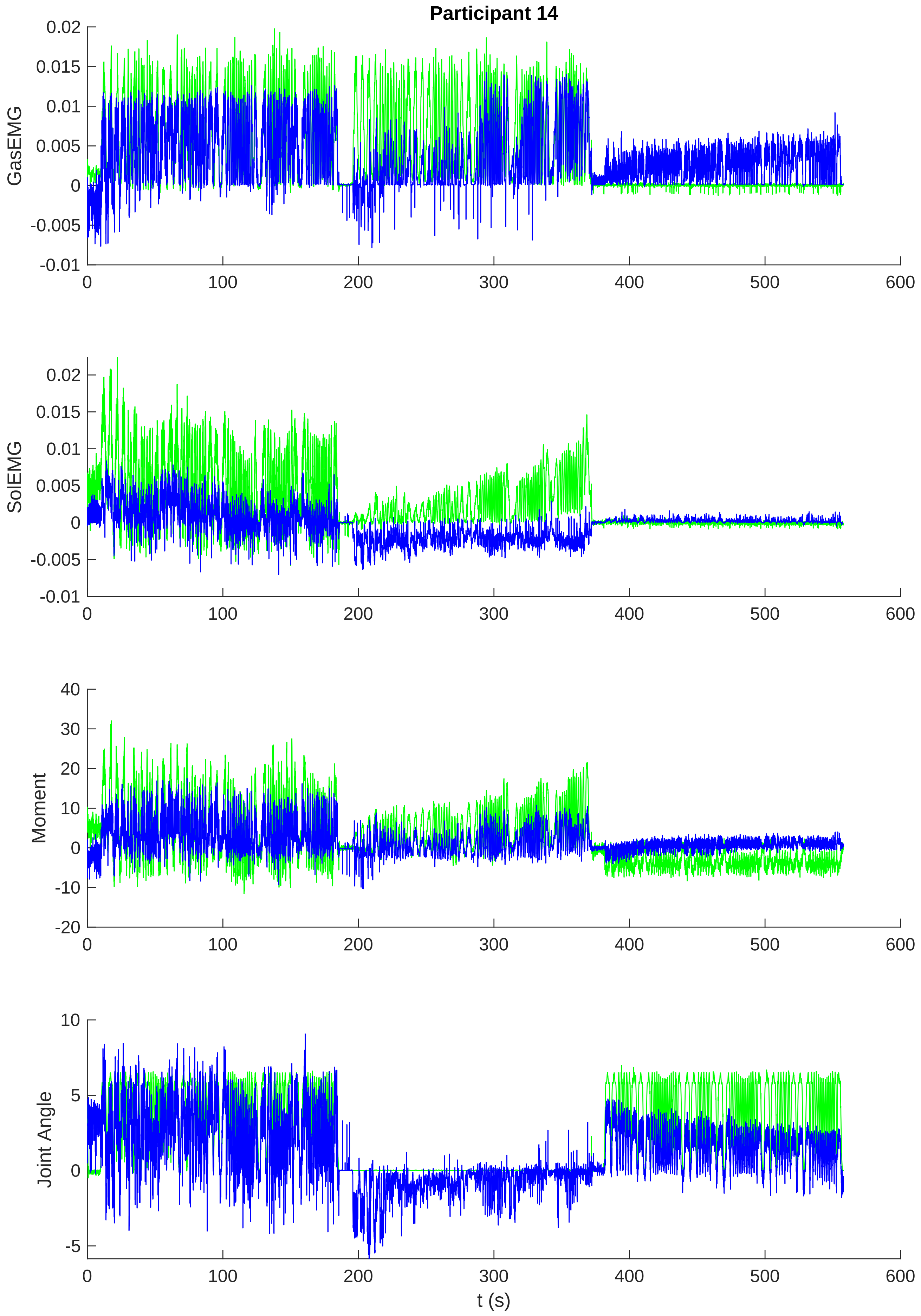
Participant 14
GasEMG
0.02
0.015
0.01
0.005
0
-0.005
-0.01
0
100
200
300
400
500
600
SolEMG
0.02
0.015
0.01
0.005
0
-0.005
-0.01
Moment
40
30
20
10
0
-10
-20
Joint Angle
10
5
0
-5
t (s)

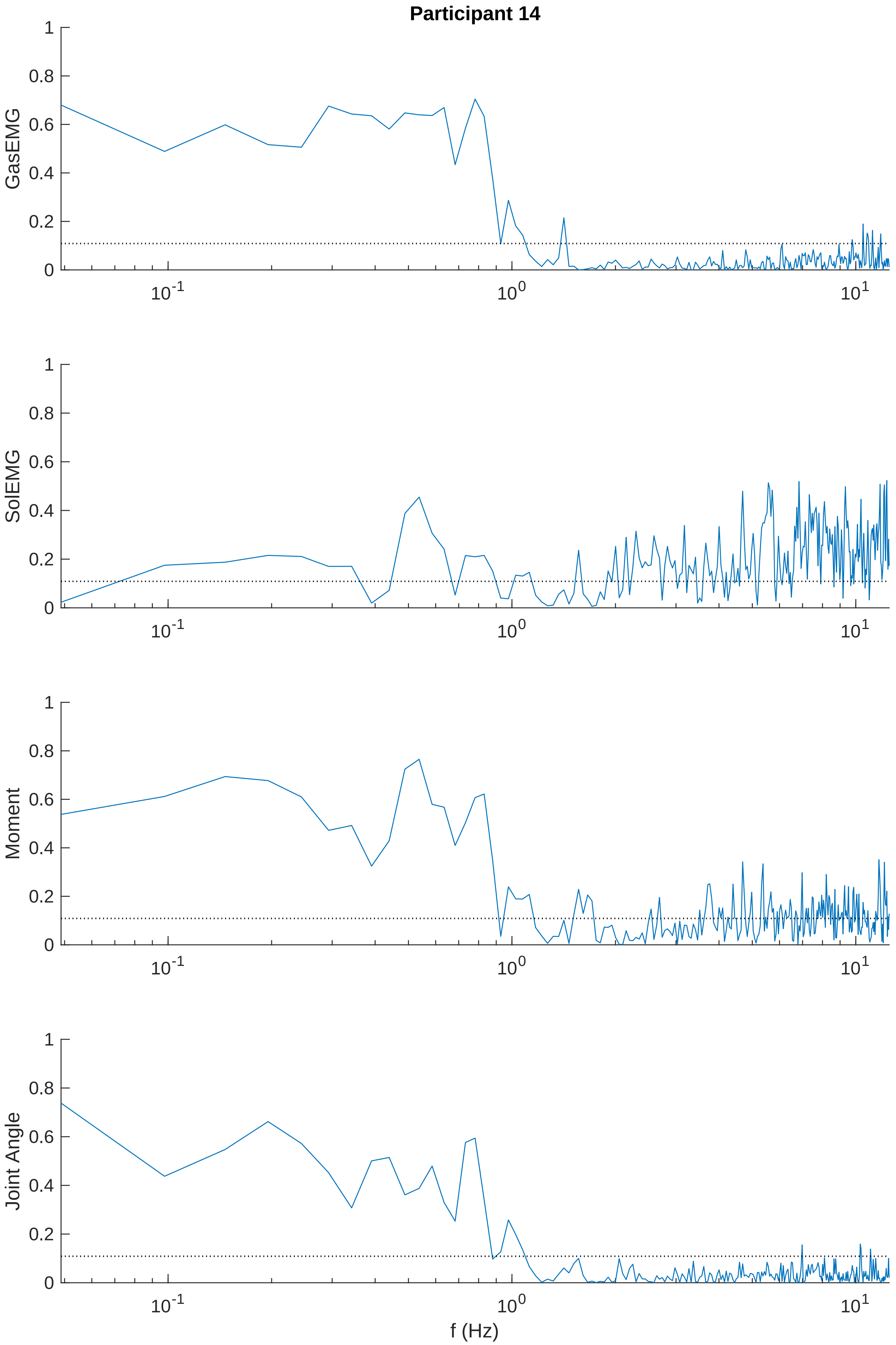

Participant 14
GasEMG
SolEMG
Moment
Joint Angle
f (Hz)

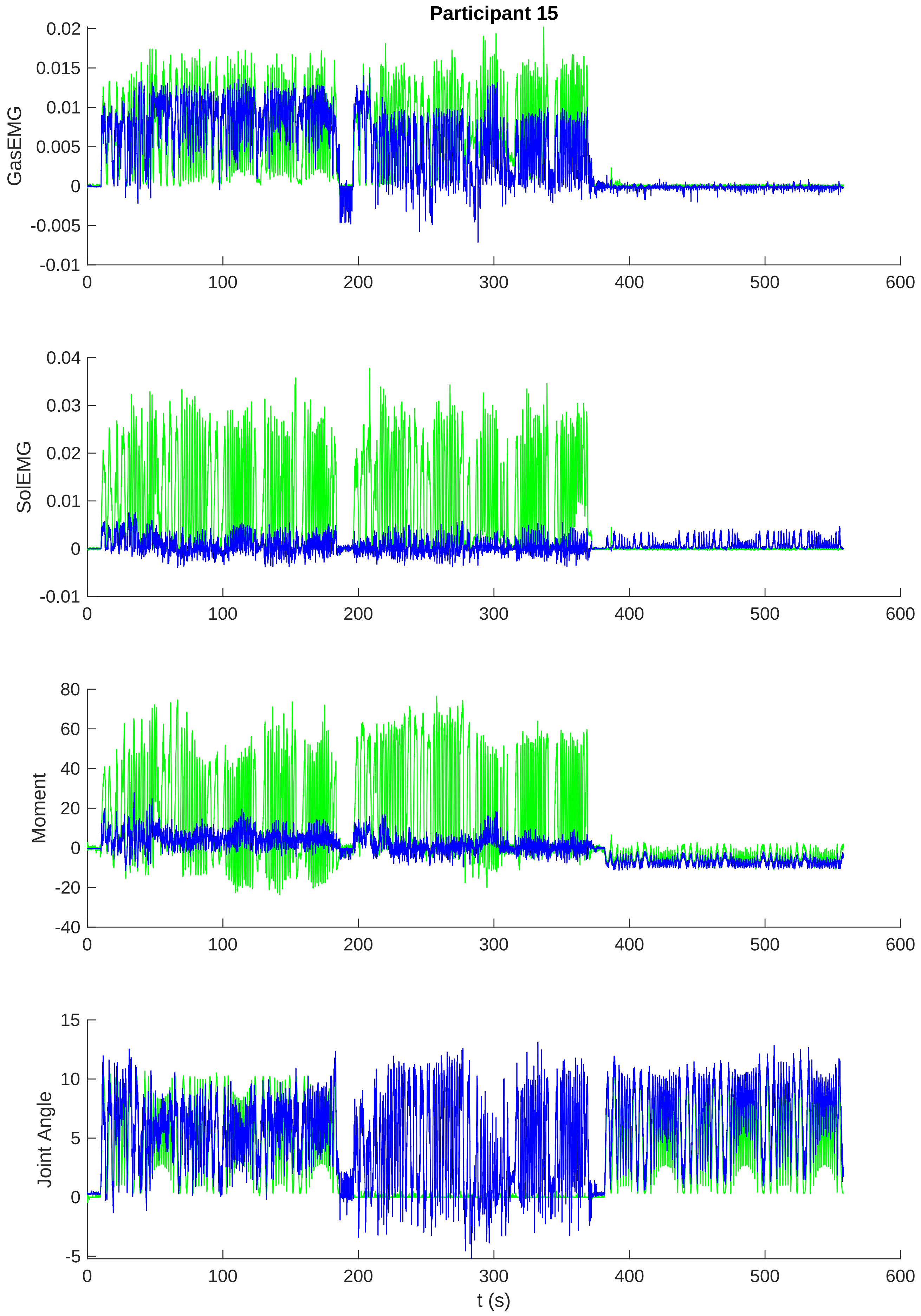

Participant 15
GasEMG
SolEMG
Moment
Joint Angle
t (s)

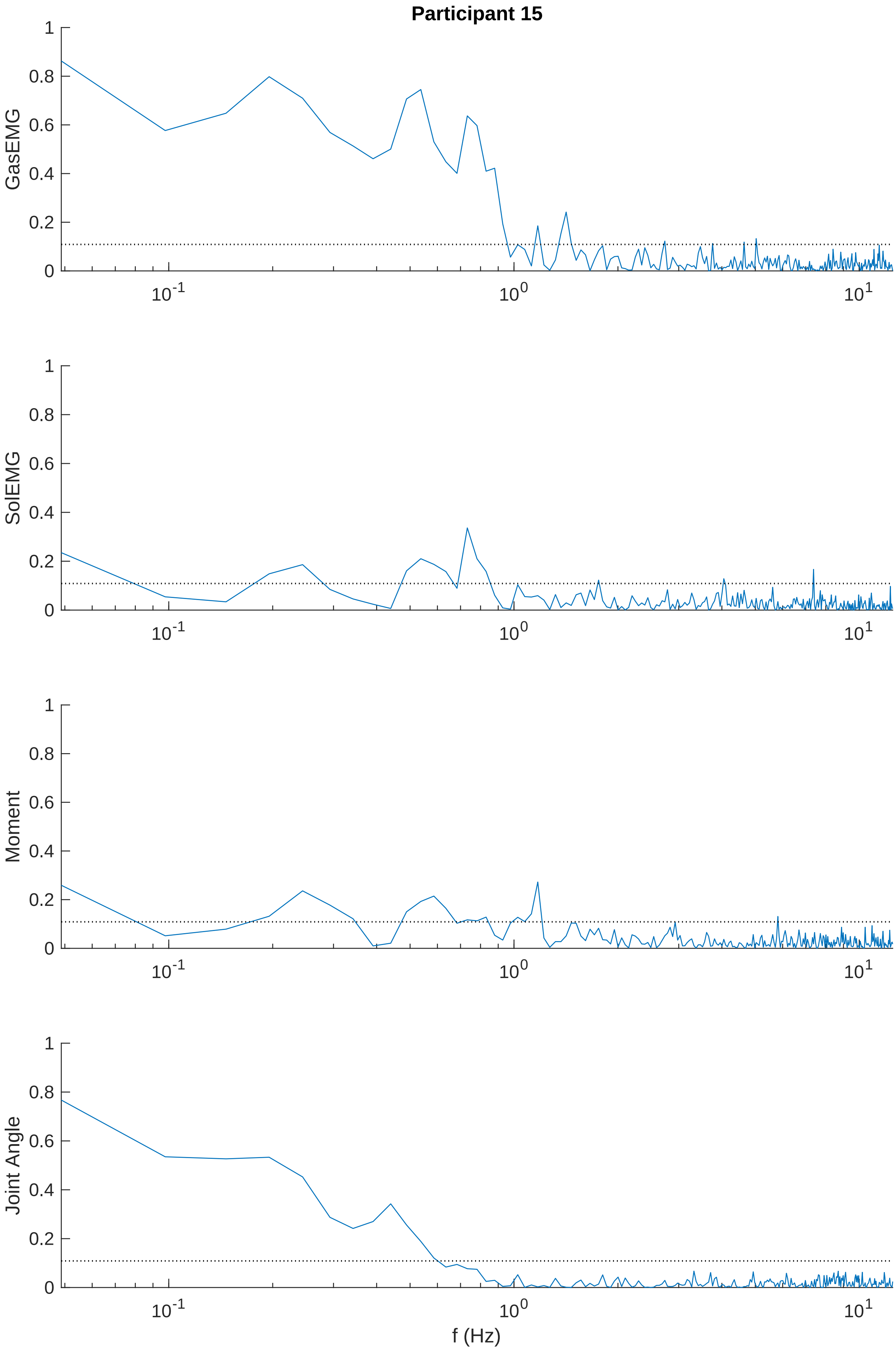
Participant 15
GasEMG
SolEMG
Moment
Joint Angle
1
0.8
0.6
0.4
0.2
0
10^{-1}
10^{0}
10^{1}
f (Hz)

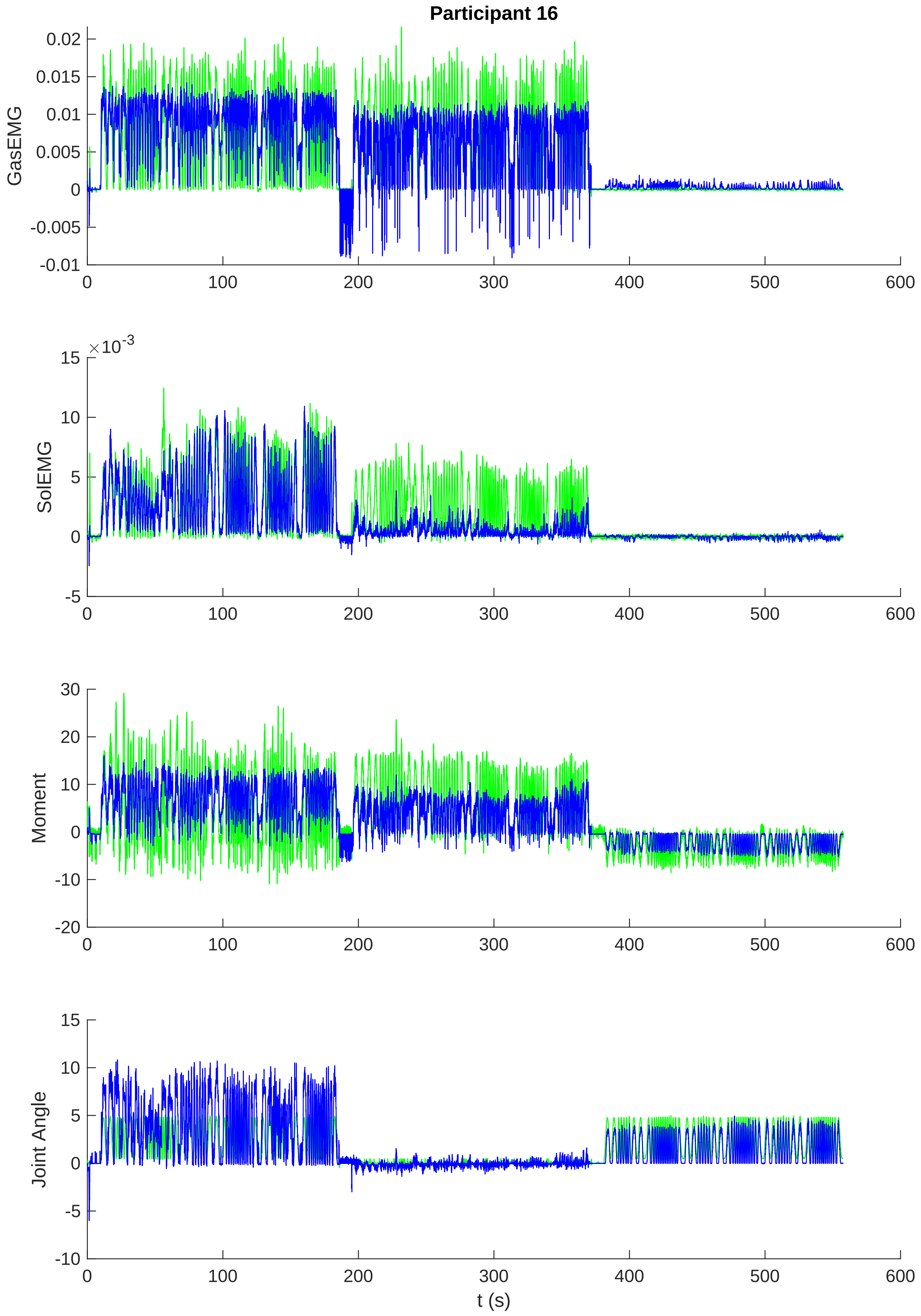
Participant 16
GasEMG
SolEMG
×10^-3
Moment
Joint Angle
t (s)

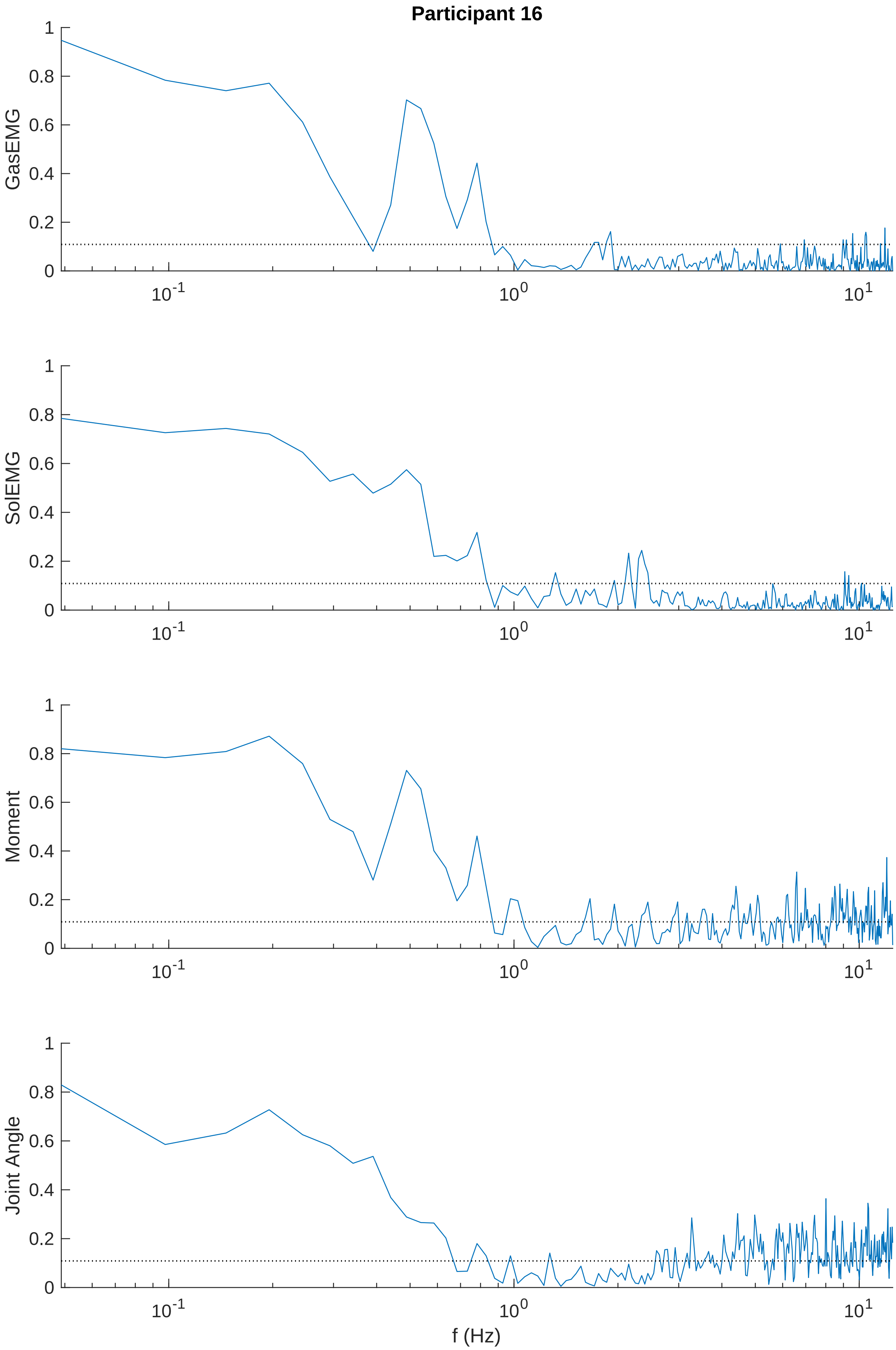

Participant 16
GasEMG
SolEMG
Moment
Joint Angle
0
0.2
0.4
0.6
0.8
1
10^{-1}
10^{0}
10^{1}
f (Hz)

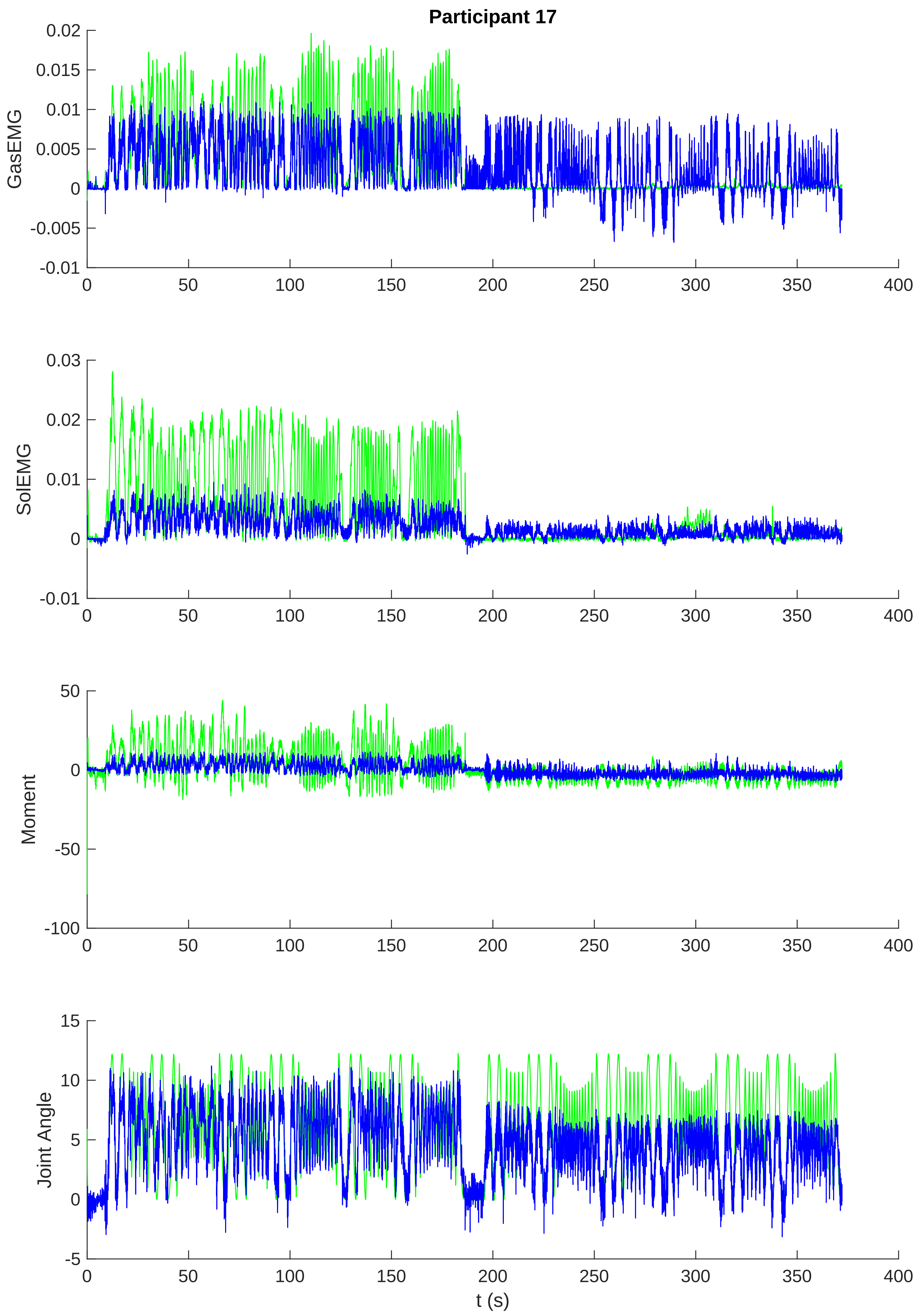
Participant 17
GasEMG
SolEMG
Moment
Joint Angle
t (s)

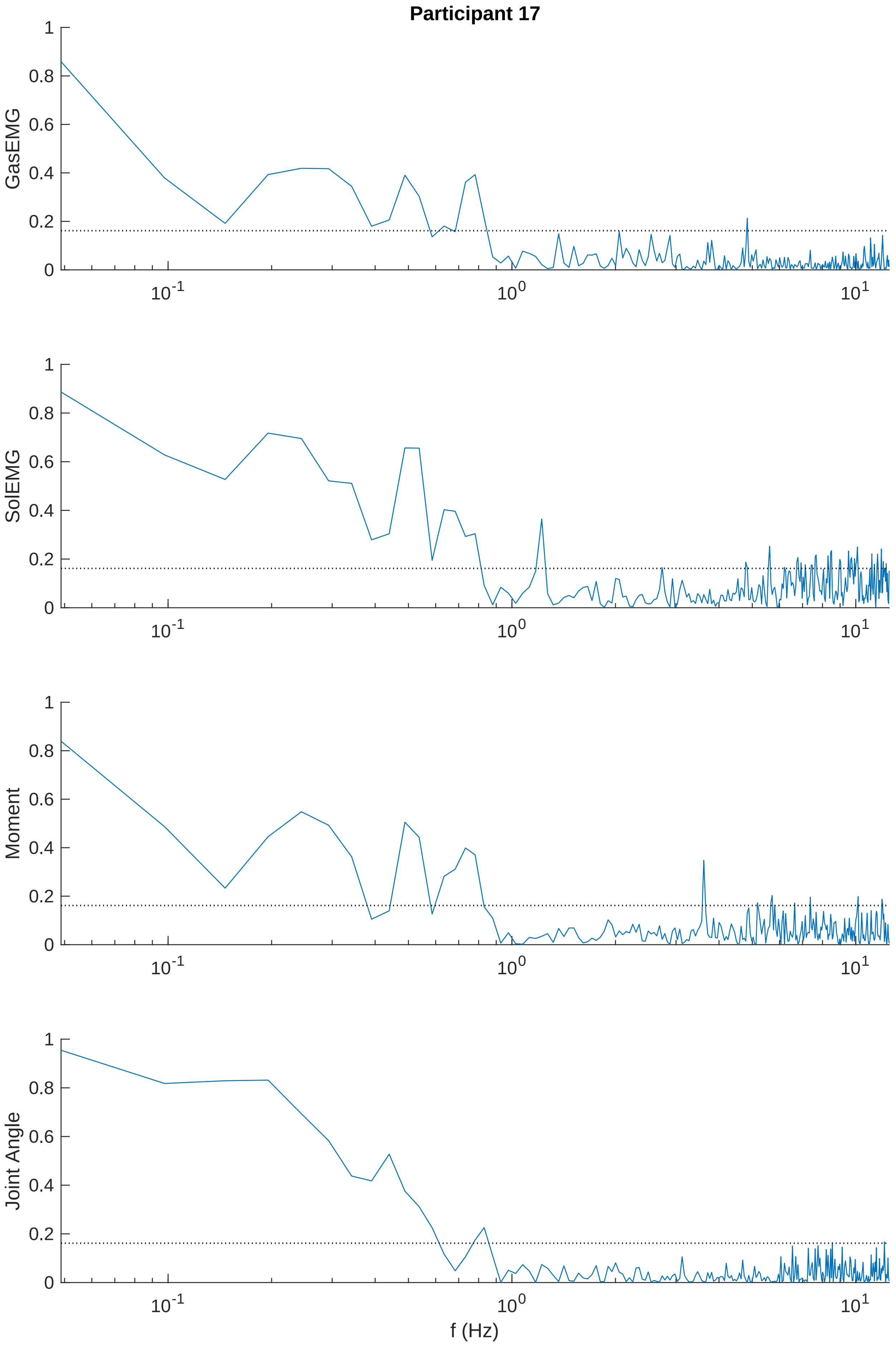
Participant 17
GasEMG
SolEMG
Moment
Joint Angle
0
0.2
0.4
0.6
0.8
1
10^{-1}
10^{0}
10^{1}
f (Hz)

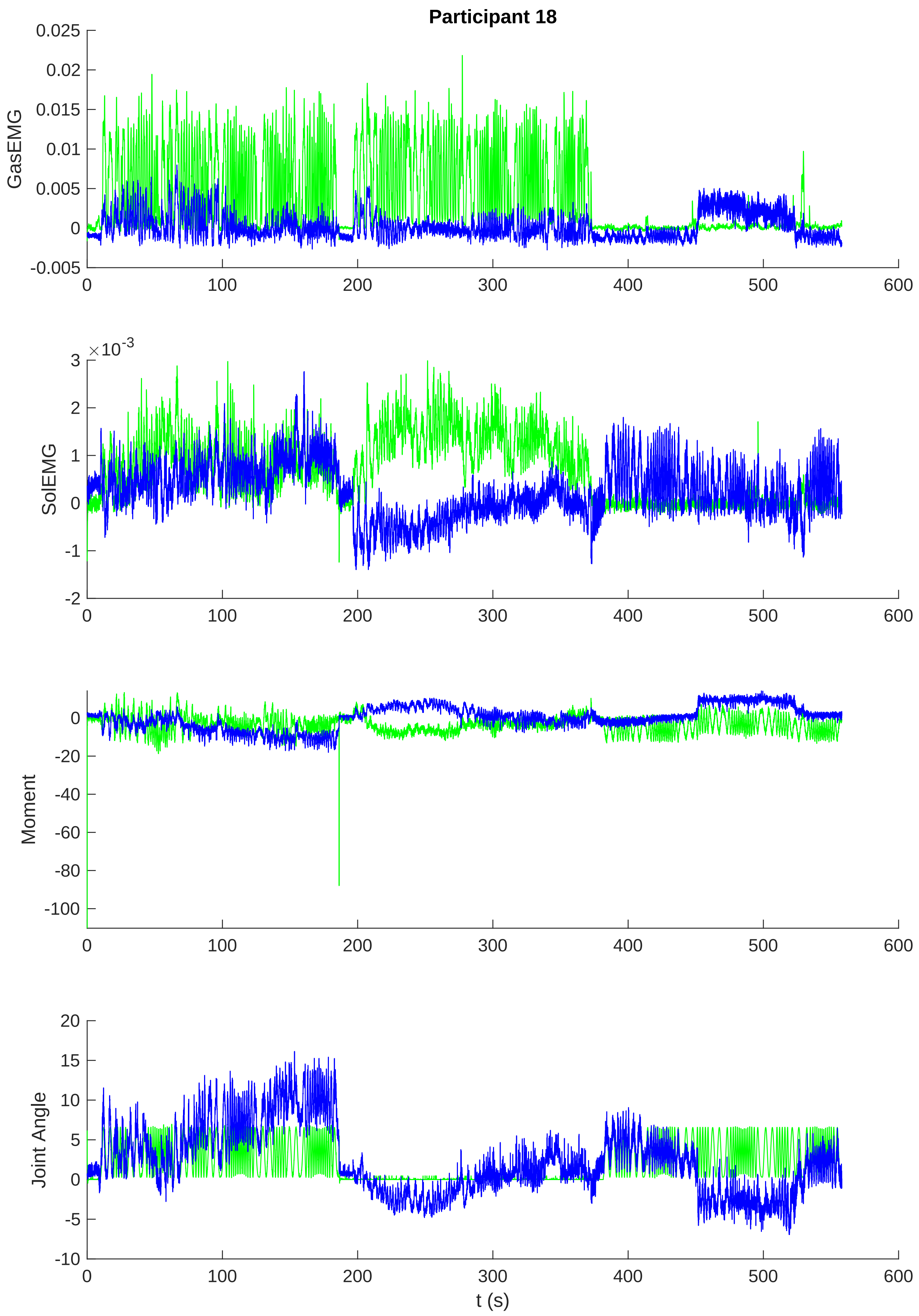

Participant 18
GasEMG
0.025
0.02
0.015
0.01
0.005
0
-0.005
SolEMG
×10^-3
3
2
1
0
-1
-2
Moment
0
-20
-40
-60
-80
-100
Joint Angle
20
15
10
5
0
-5
-10
0
100
200
300
400
500
600
t (s)

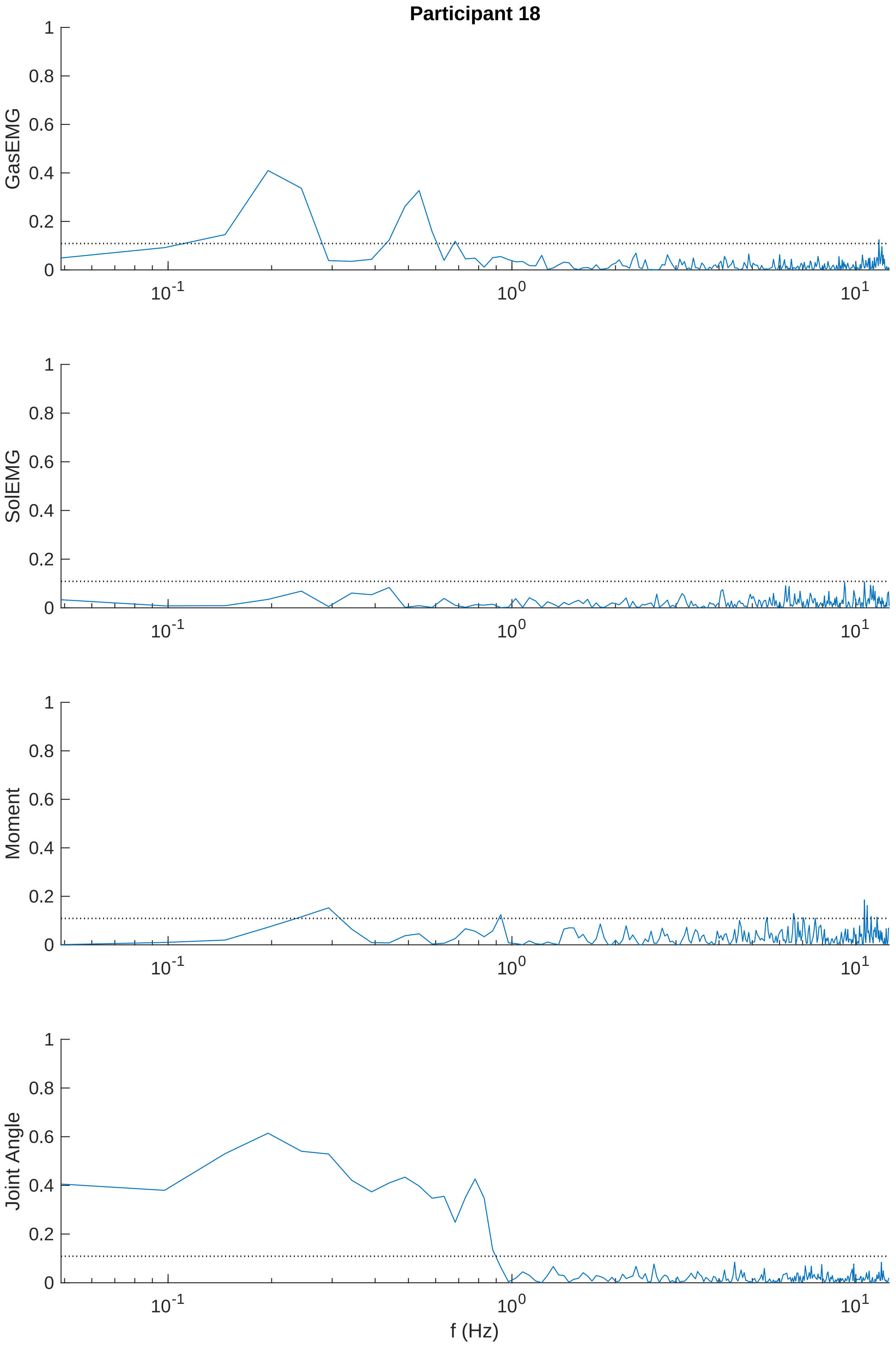

Participant 18
GasEMG
SolEMG
Moment
Joint Angle
1
0.8
0.6
0.4
0.2
0
10^{-1}
10^{0}
10^{1}
f (Hz)

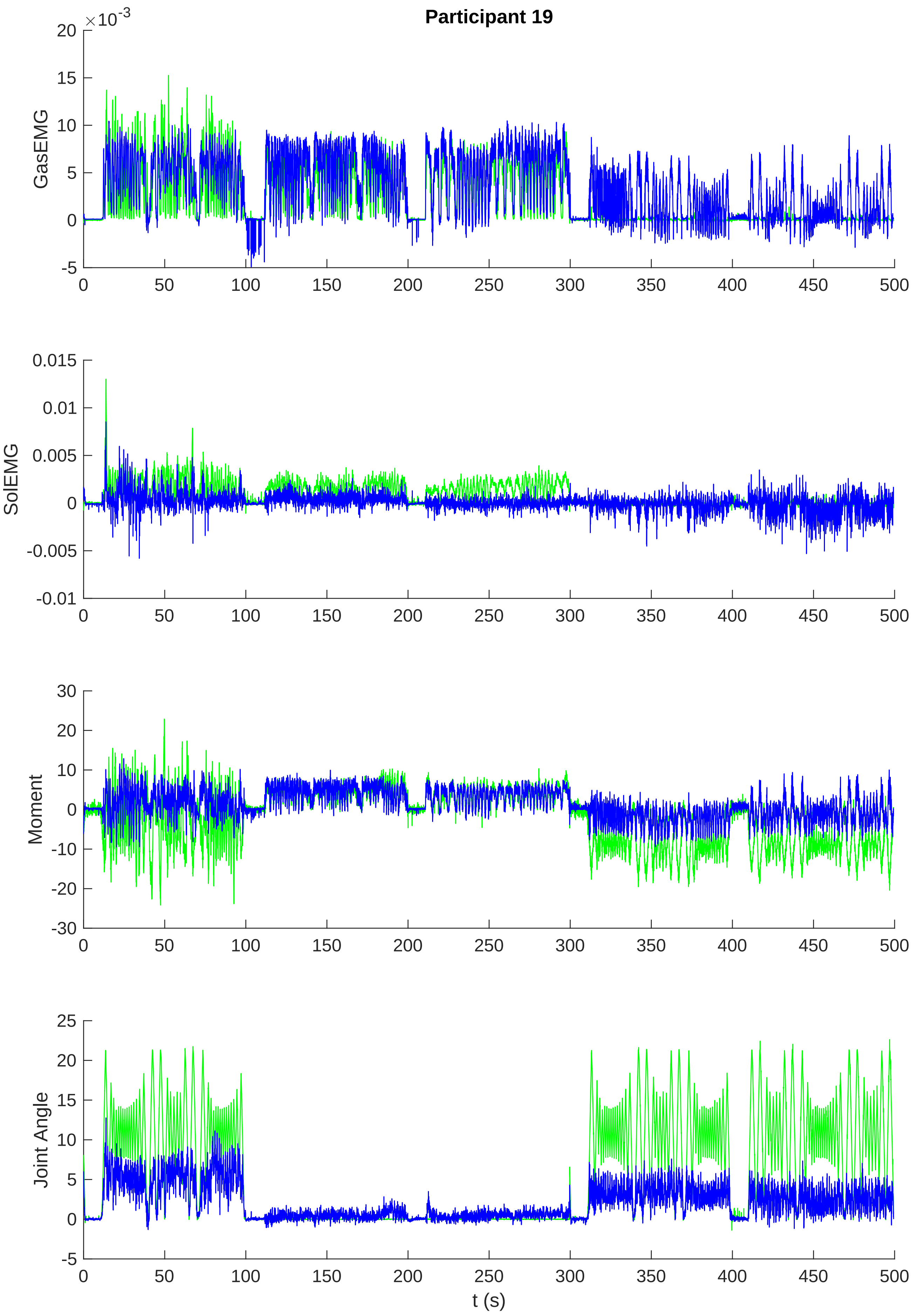
Participant 19
GasEMG
×10^-3
SolEMG
Moment
Joint Angle
t (s)

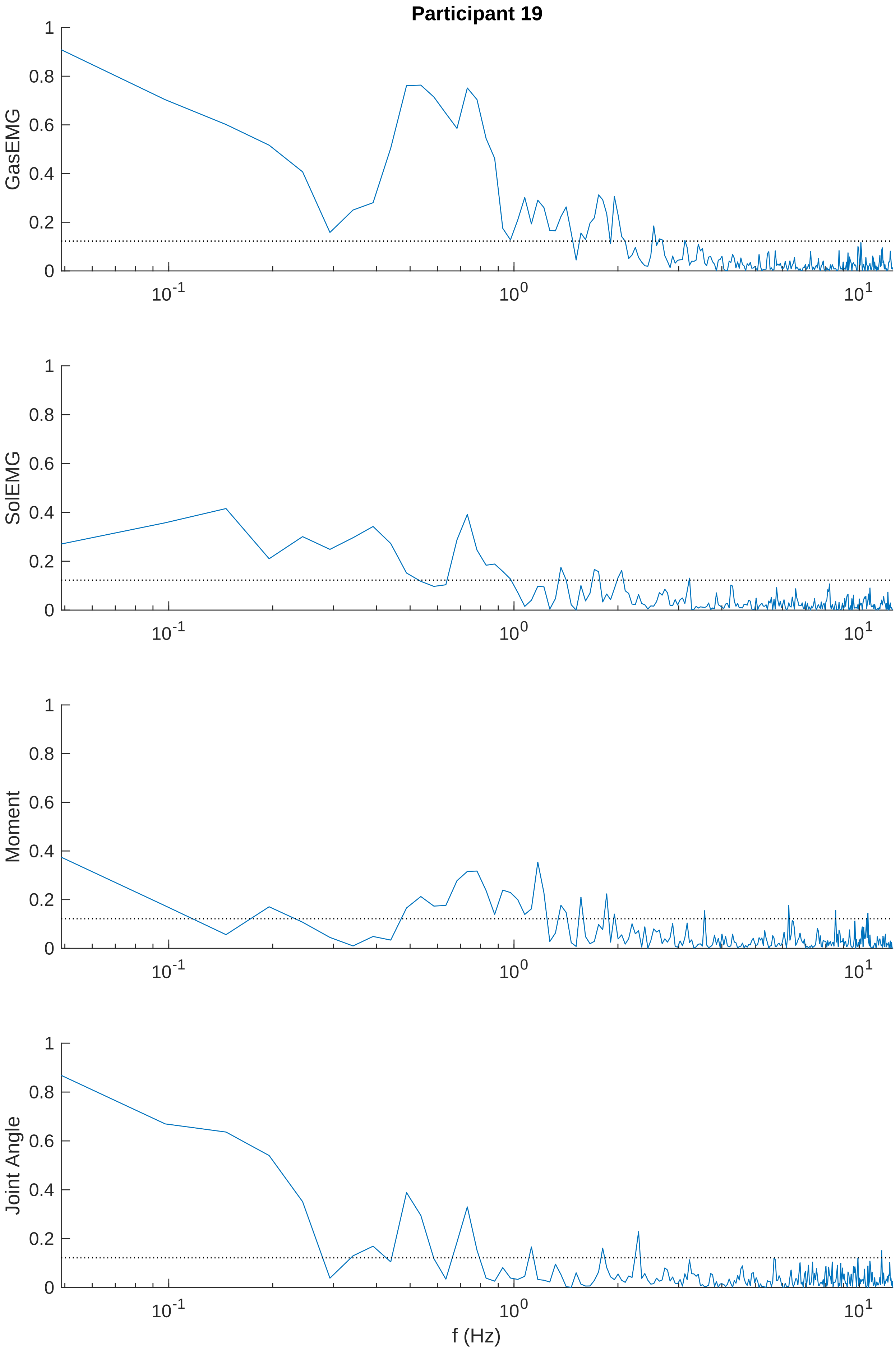

Participant 19
GasEMG
SolEMG
Moment
Joint Angle
1
0.8
0.6
0.4
0.2
0
10^{-1}
10^{0}
10^{1}
f (Hz)

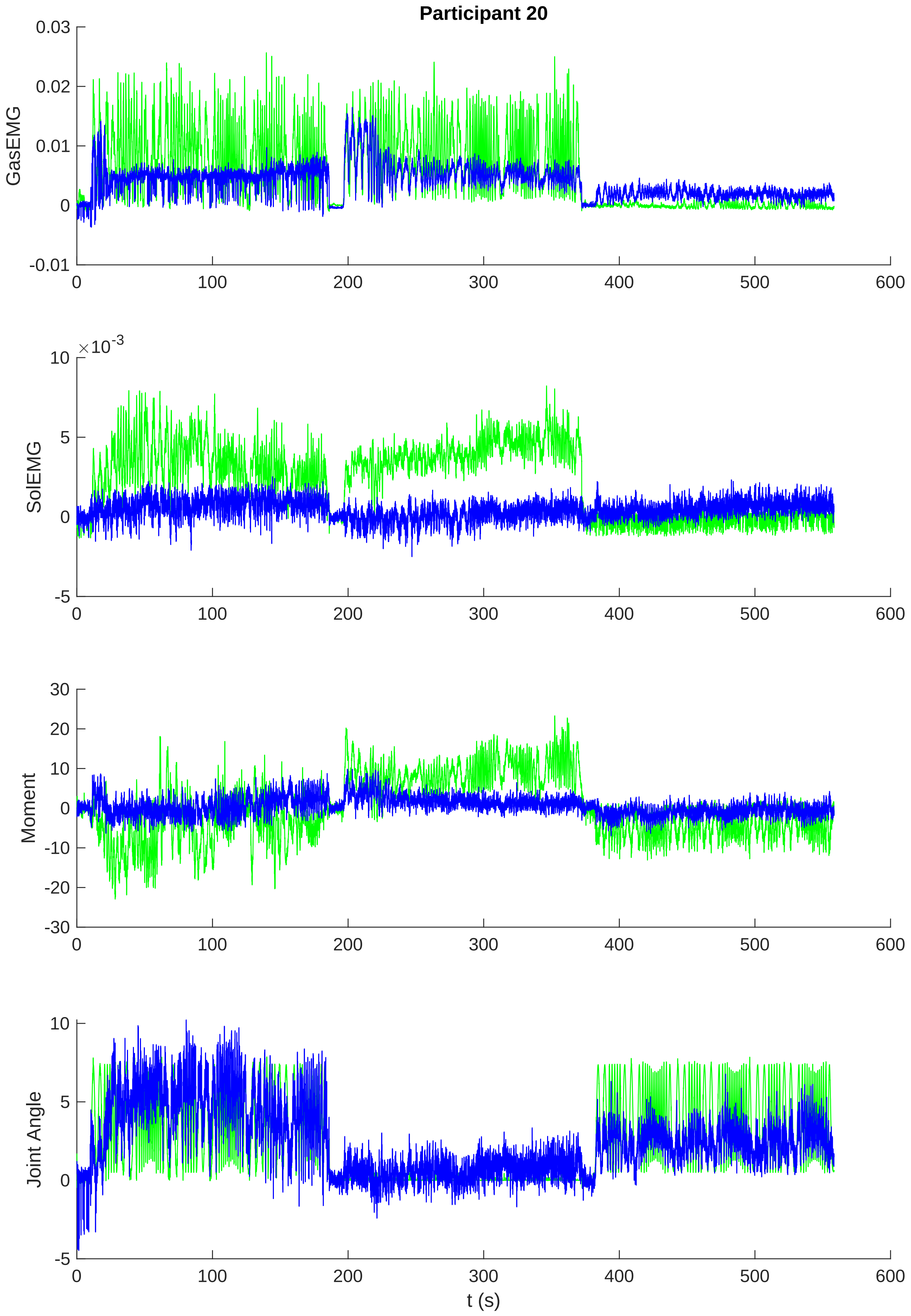

Participant 20
GasEMG
0.03
0.02
0.01
0
-0.01
0
100
200
300
400
500
600
SolEMG
×10^-3
10
5
0
-5
Moment
30
20
10
0
-10
-20
-30
Joint Angle
10
5
0
-5
t (s)

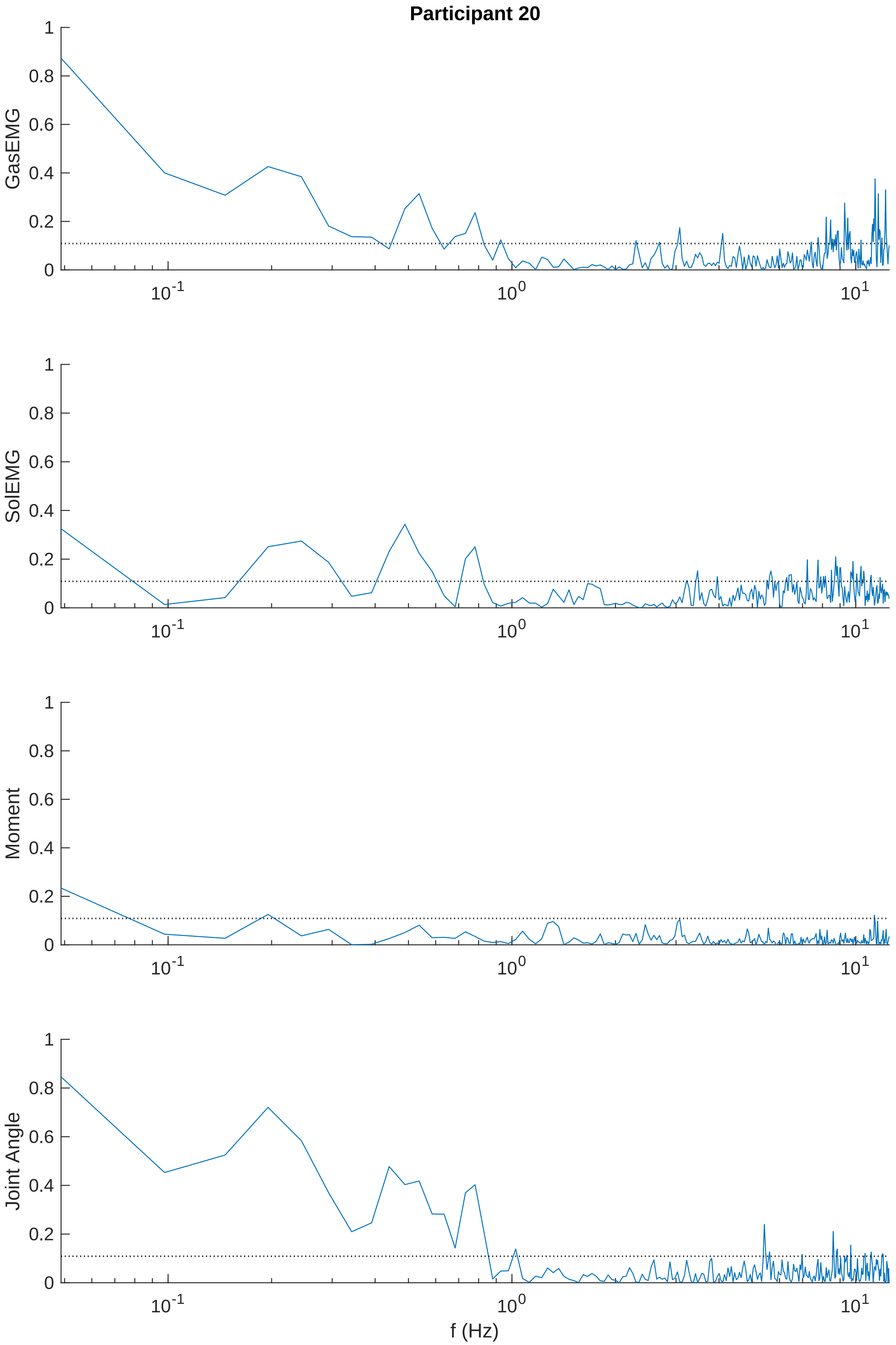
Participant 20
GasEMG
SolEMG
Moment
Joint Angle
f (Hz)
0
0.2
0.4
0.6
0.8
1
10^{-1}
10^{0}
10^{1}

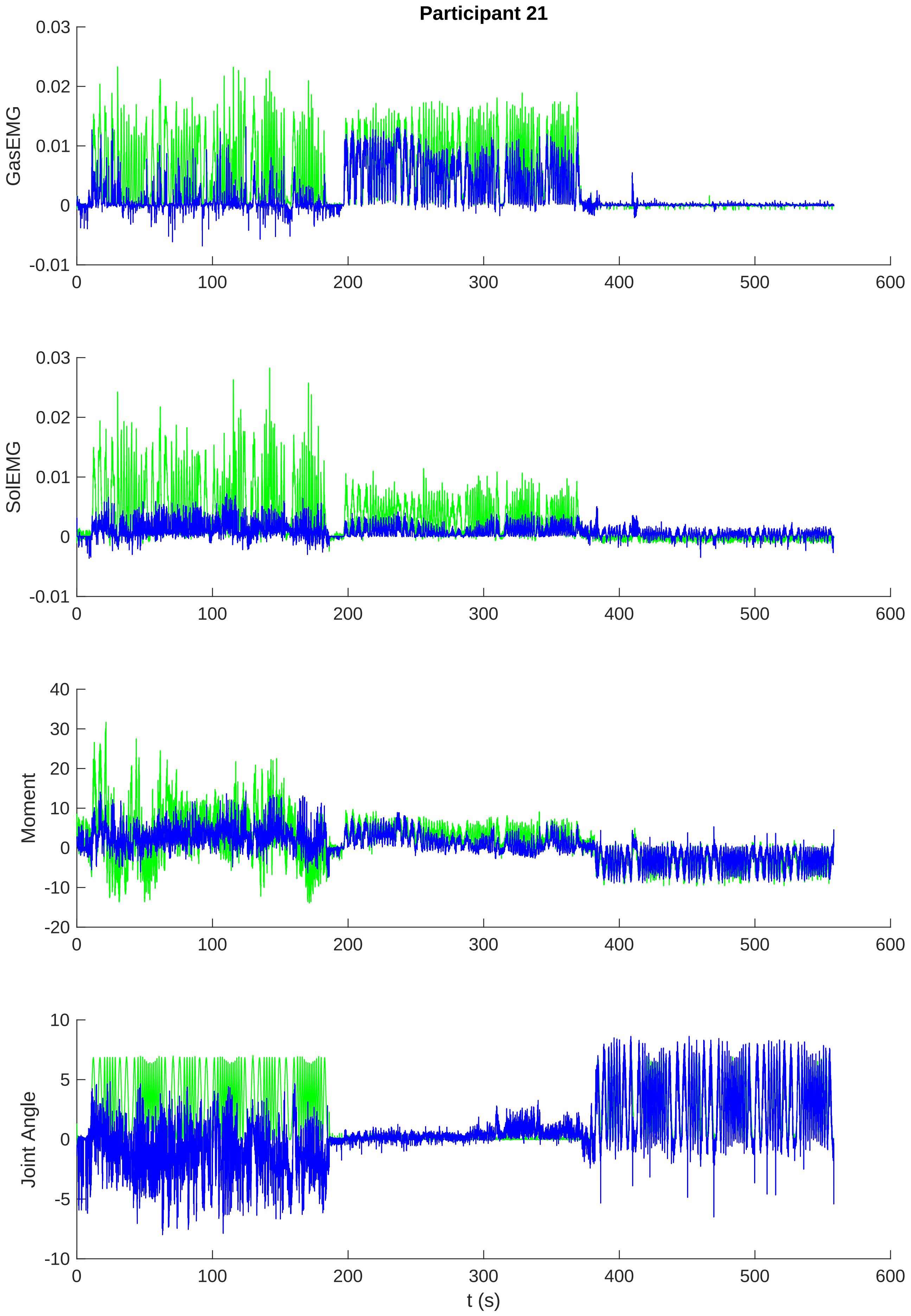

Participant 21
GasEMG
SolEMG
Moment
Joint Angle
t (s)

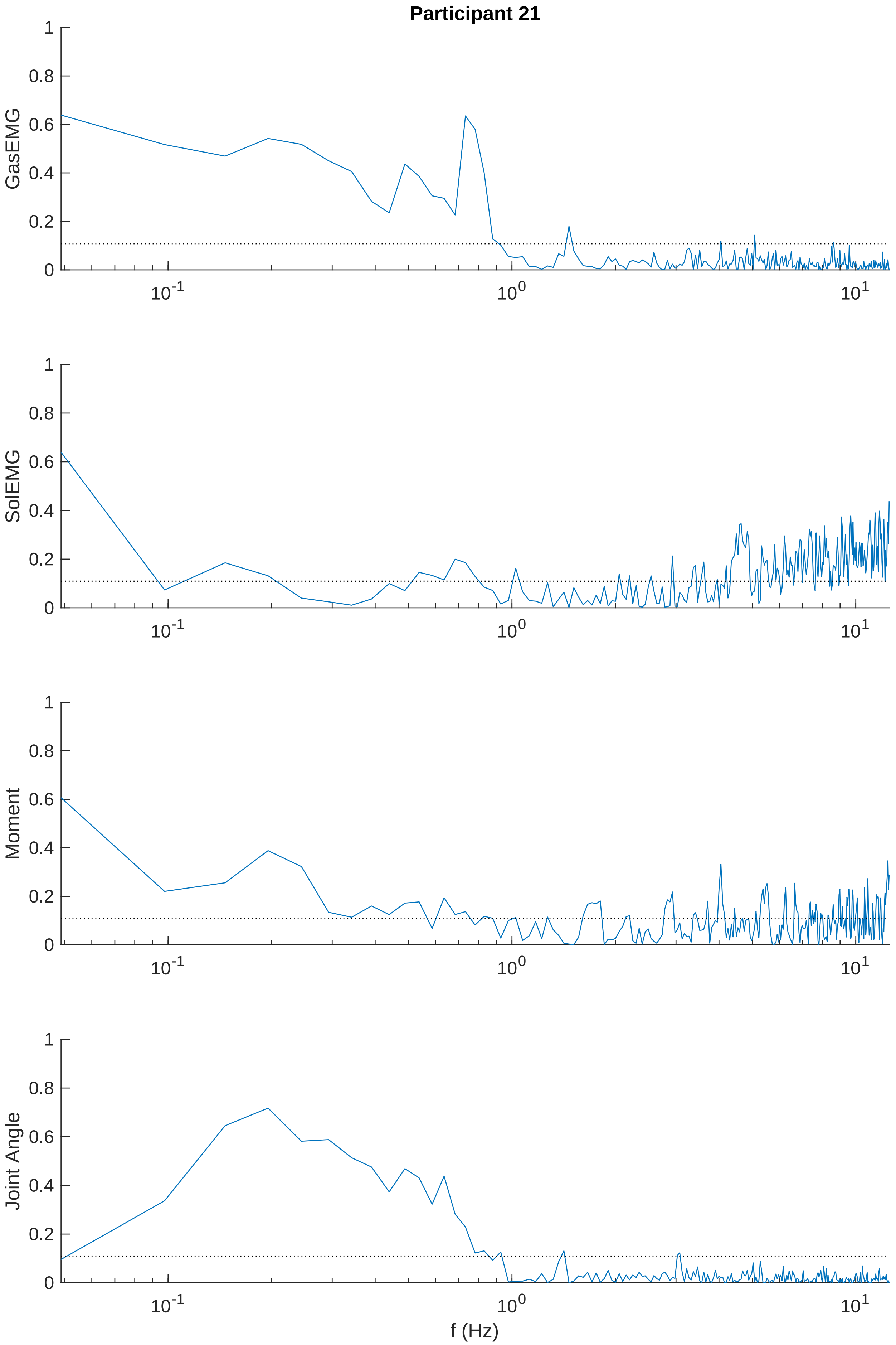
Participant 21
GasEMG
SolEMG
Moment
Joint Angle
0
0.2
0.4
0.6
0.8
1
10^{-1}
10^{0}
10^{1}
f (Hz)

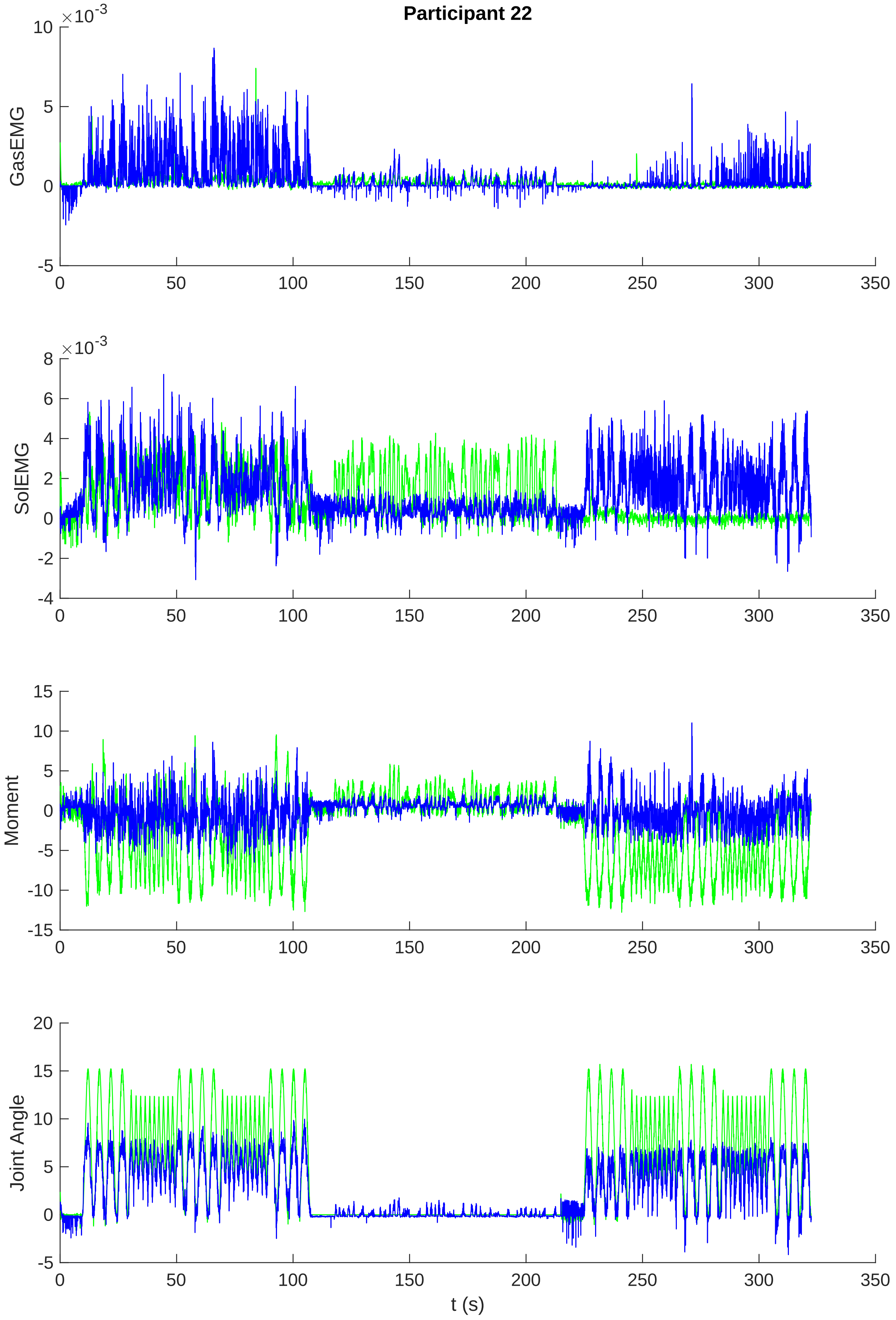

Participant 22
GasEMG
×10^-3
SolEMG
Moment
Joint Angle
t (s)

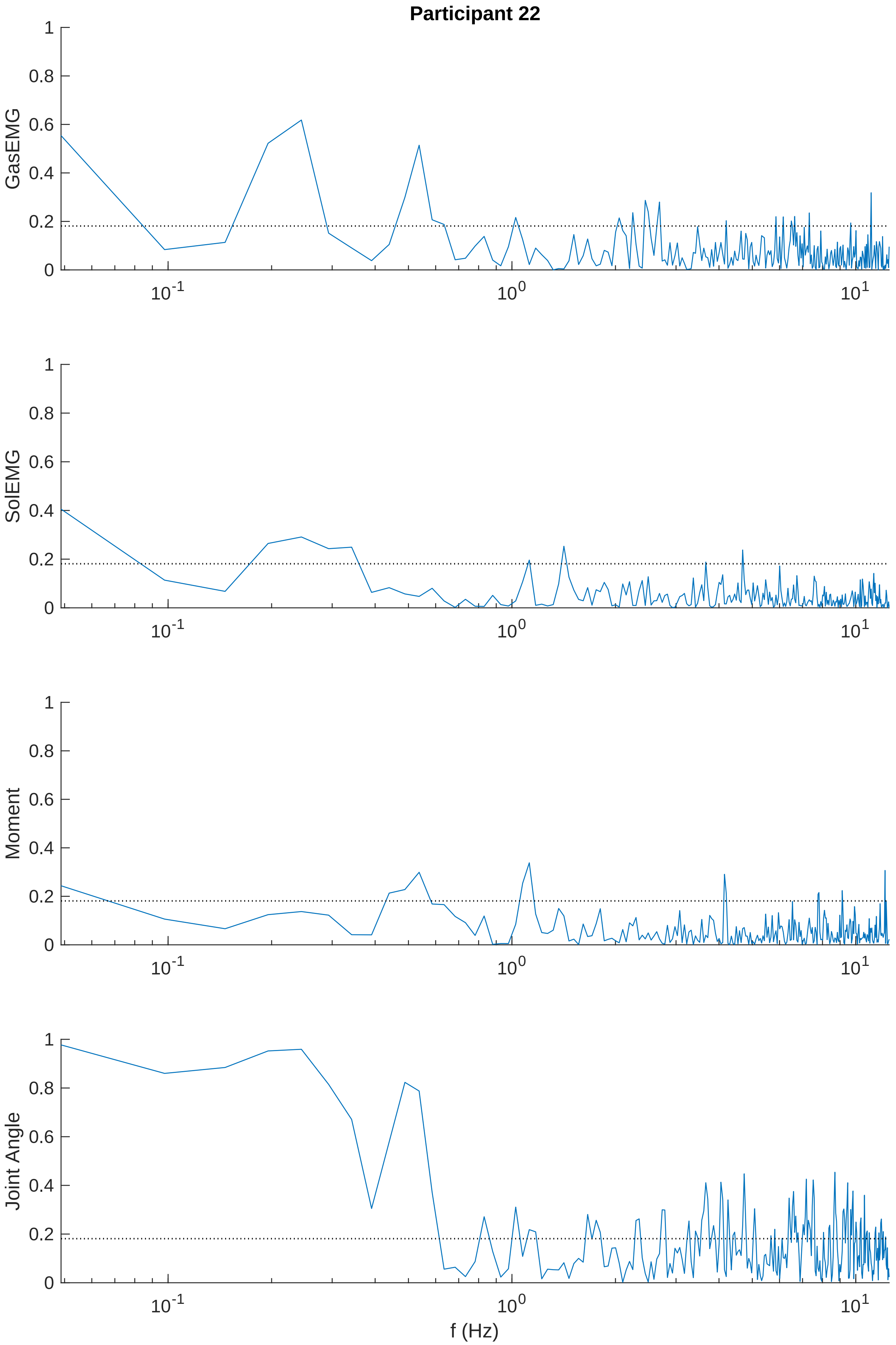
Participant 22
GasEMG
SolEMG
Moment
Joint Angle
0
0.2
0.4
0.6
0.8
1
10^{-1}
10^{0}
10^{1}
f (Hz)

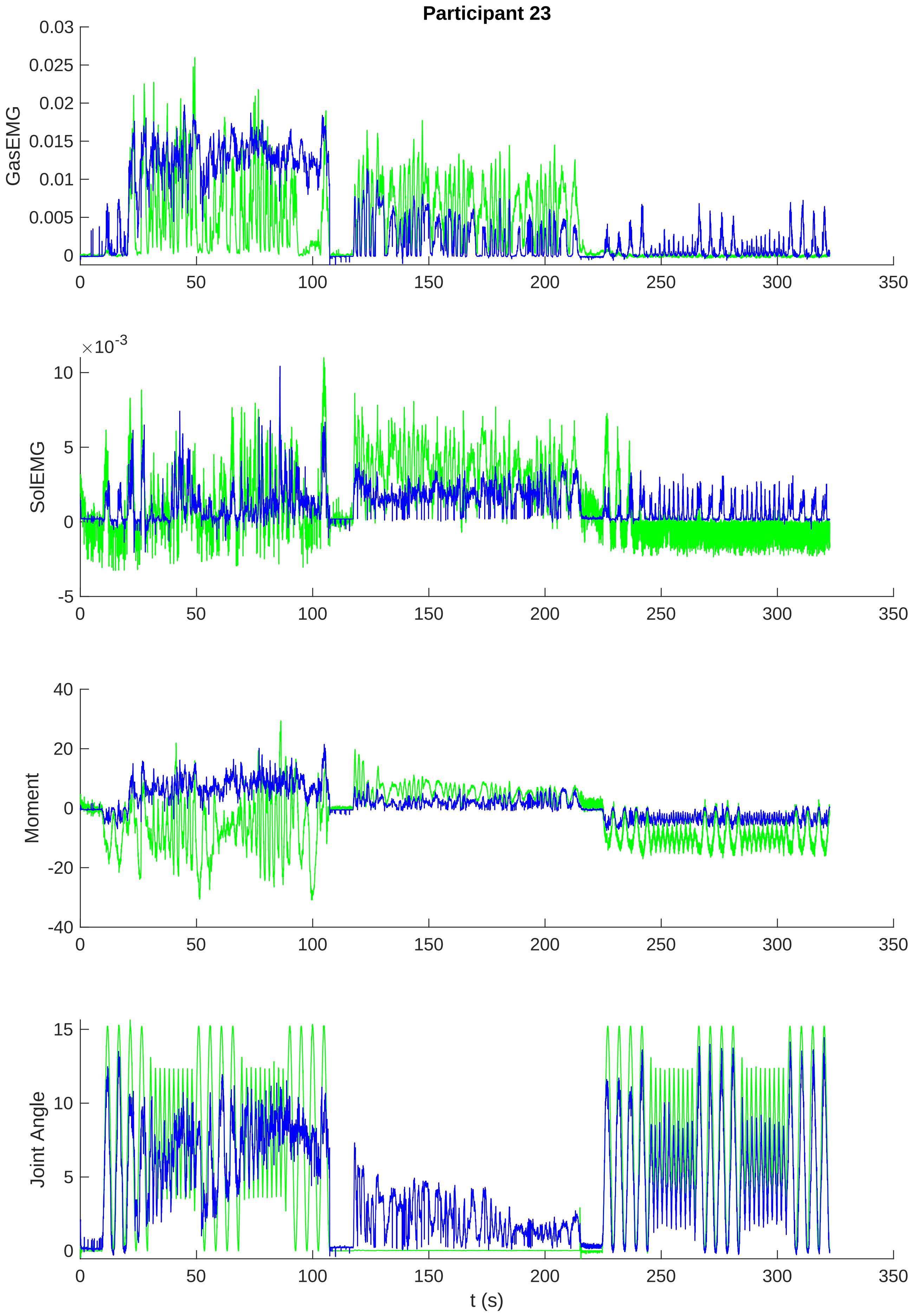

Participant 23
GasEMG
0.03
0.025
0.02
0.015
0.01
0.005
0
SolEMG
×10^-3
10
5
0
-5
Moment
40
20
0
-20
-40
Joint Angle
15
10
5
0
0
50
100
150
200
250
300
350
t (s)

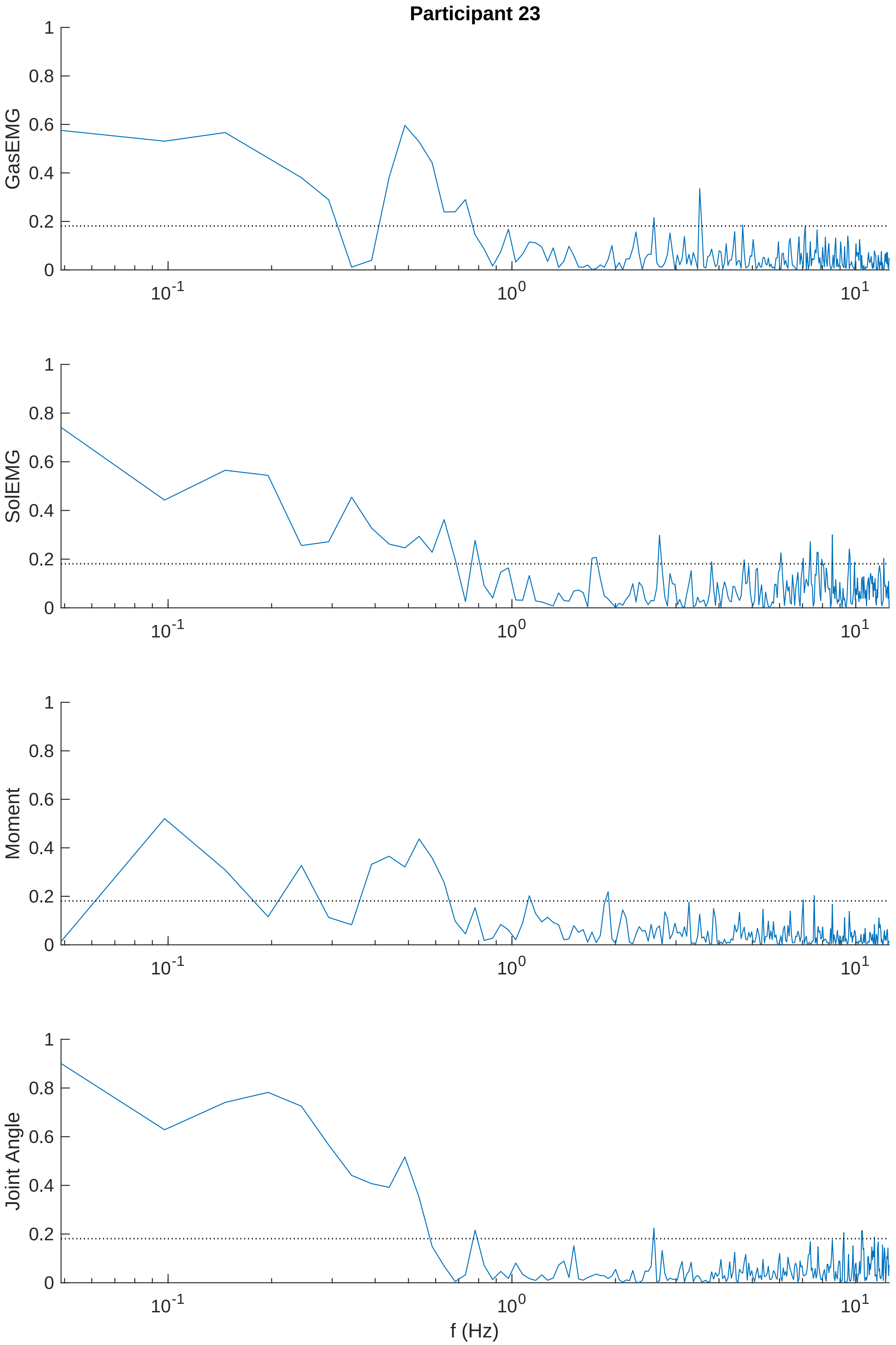

Participant 23
GasEMG
SolEMG
Moment
Joint Angle
1
0.8
0.6
0.4
0.2
0
10^{-1}
10^{0}
10^{1}
f (Hz)

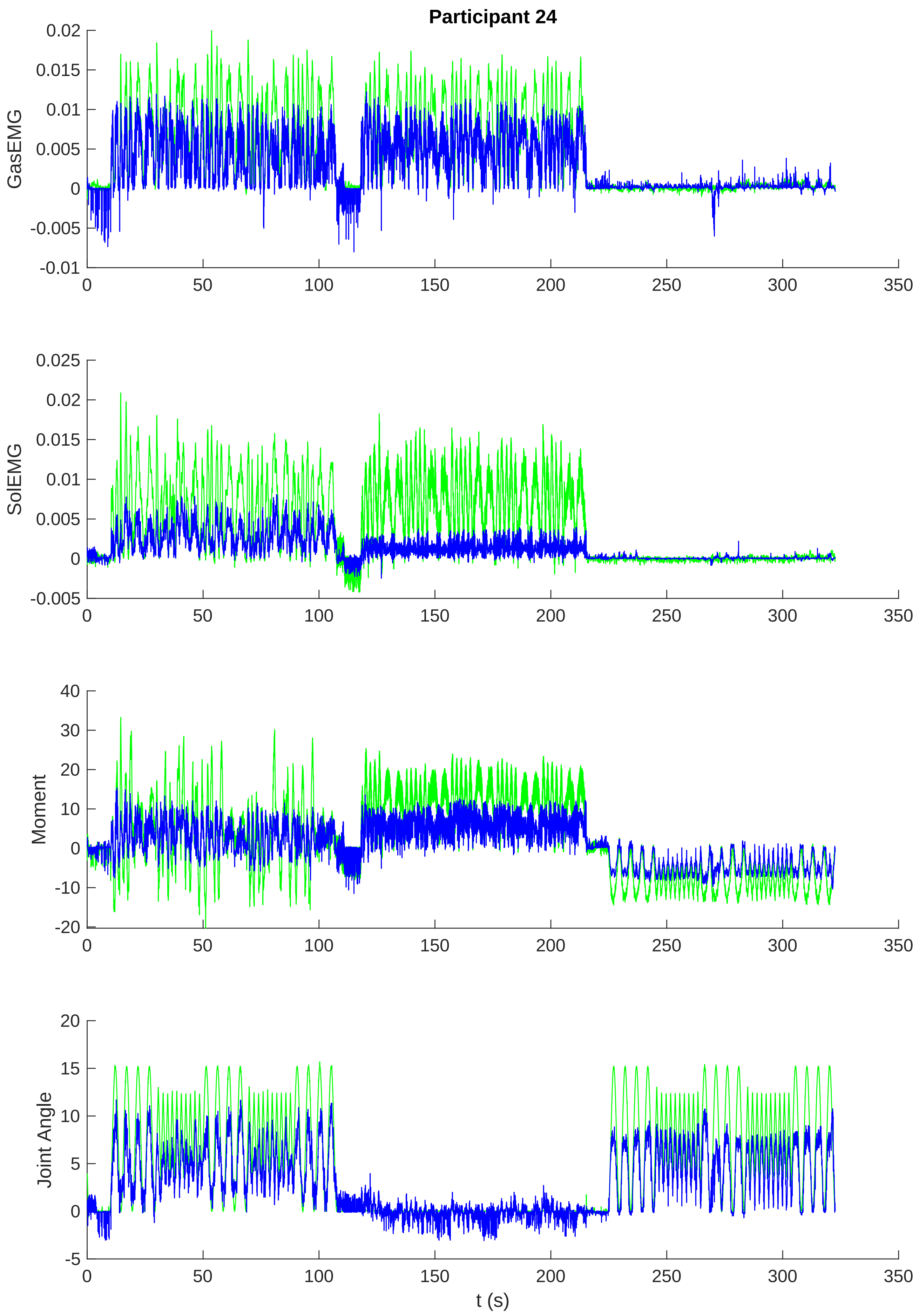
Participant 24
GasEMG
0.02
0.015
0.01
0.005
0
-0.005
-0.01
SolEMG
0.025
0.02
0.015
0.01
0.005
0
-0.005
Moment
40
30
20
10
0
-10
-20
Joint Angle
20
15
10
5
0
-5
0
50
100
150
200
250
300
350
t (s)

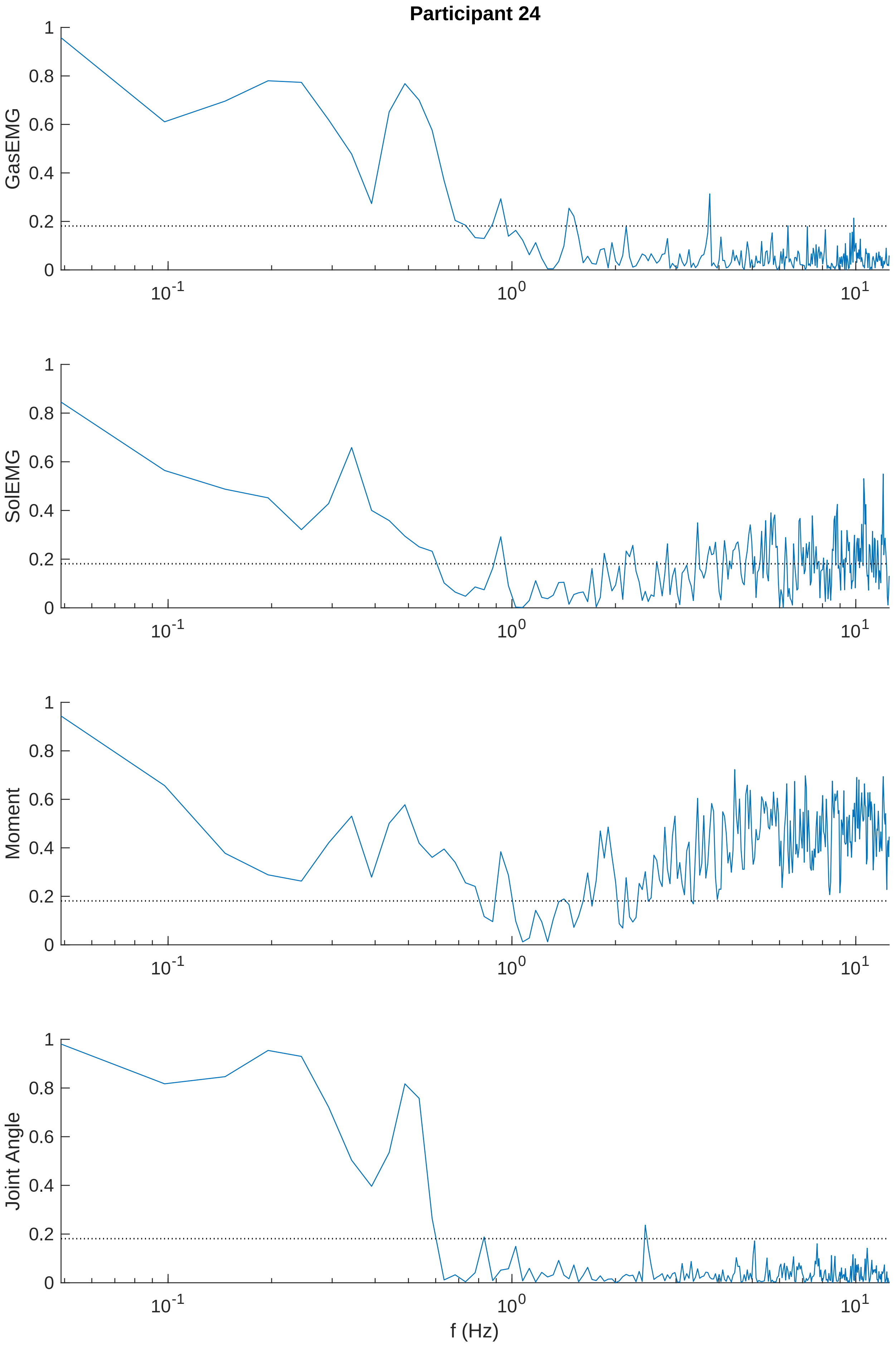

Participant 24
GasEMG
SolEMG
Moment
Joint Angle
0
0.2
0.4
0.6
0.8
1
10^{-1}
10^{0}
10^{1}
f (Hz)

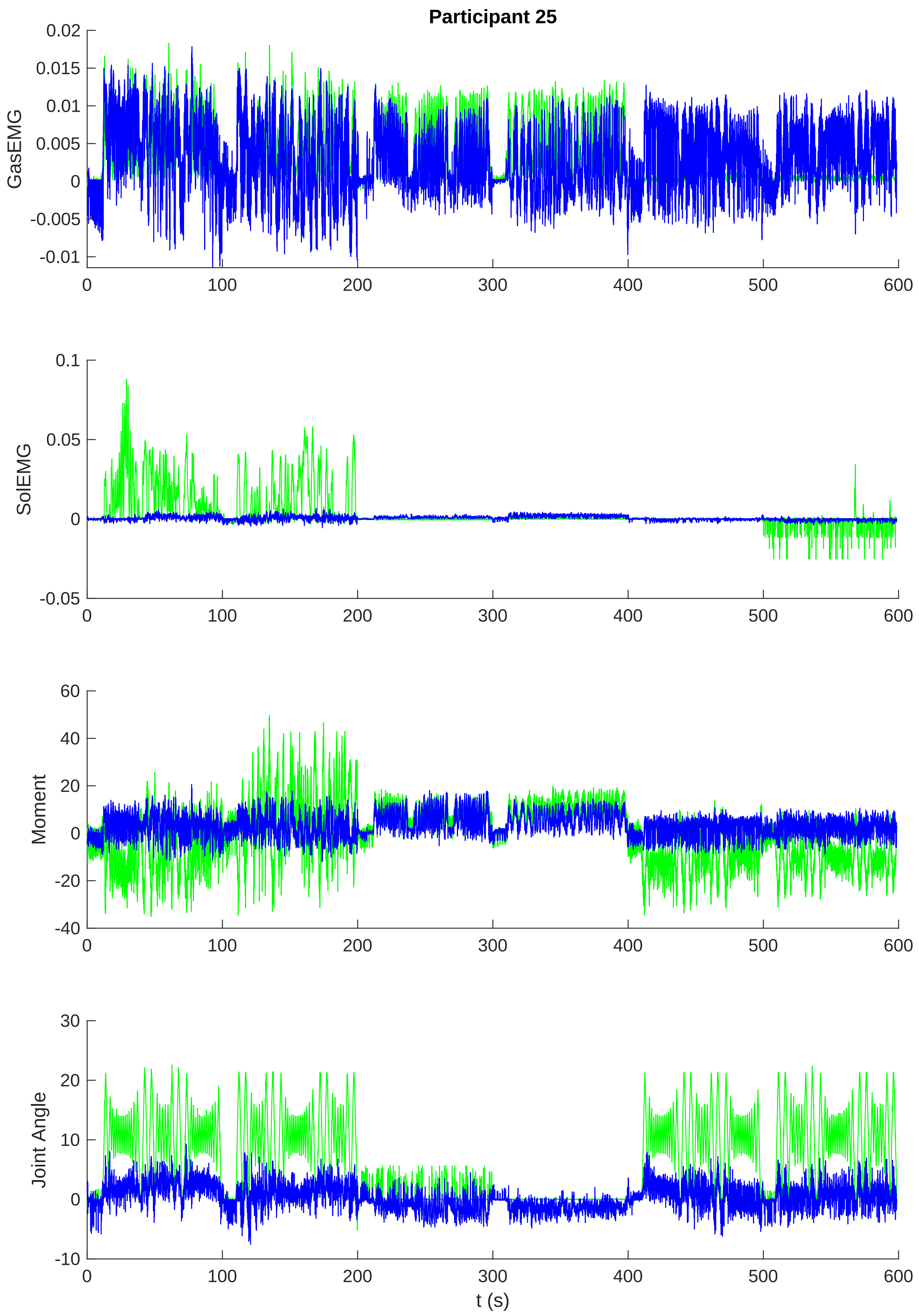

Participant 25
GasEMG
0.02
0.015
0.01
0.005
0
-0.005
-0.01
0
100
200
300
400
500
600
SolEMG
0.1
0.05
0
-0.05
Moment
60
40
20
0
-20
-40
Joint Angle
30
20
10
0
-10
t (s)

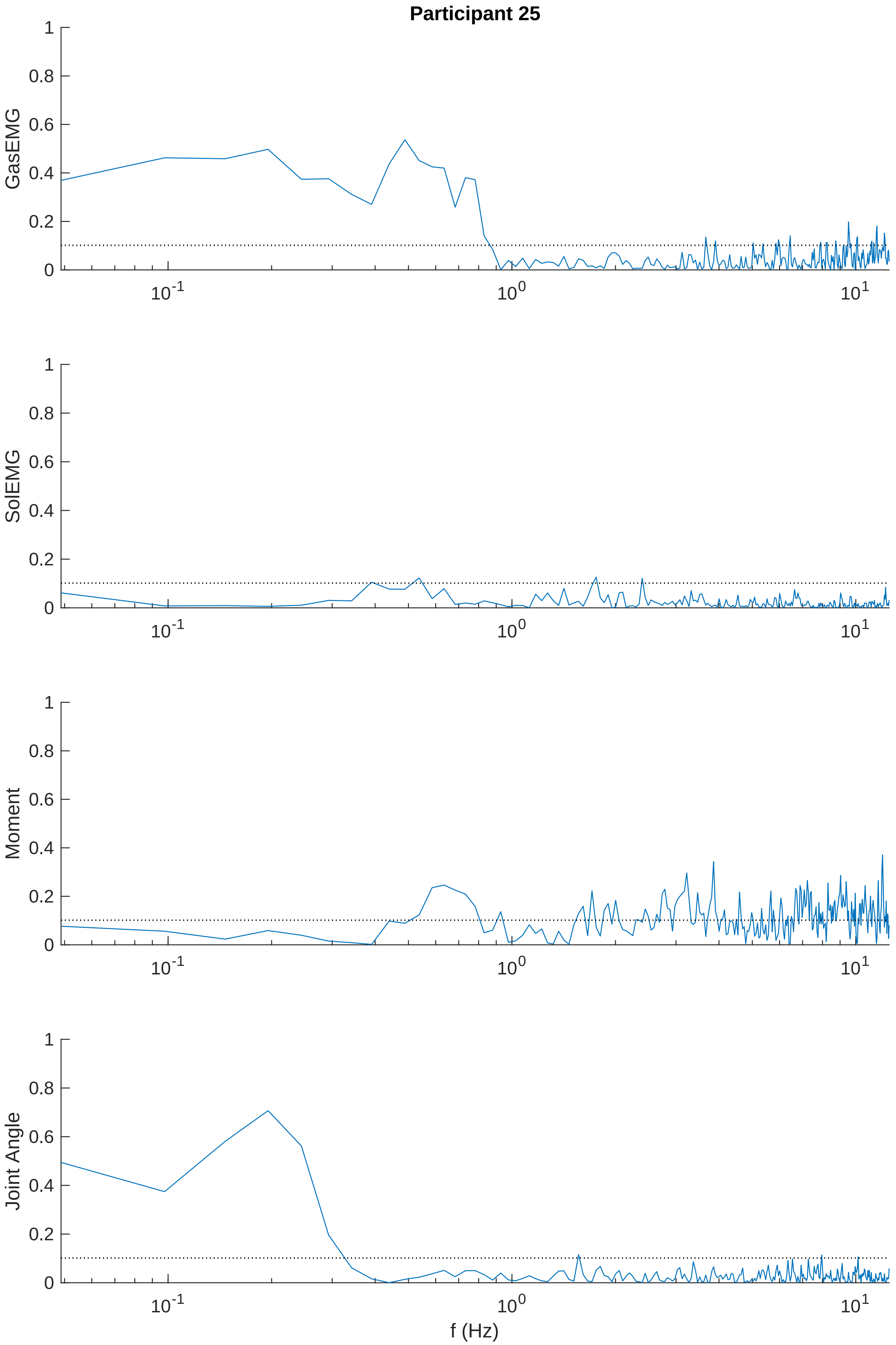

Participant 25
GasEMG
SolEMG
Moment
Joint Angle
1
0.8
0.6
0.4
0.2
0
10^{-1}
10^{0}
10^{1}
f (Hz)

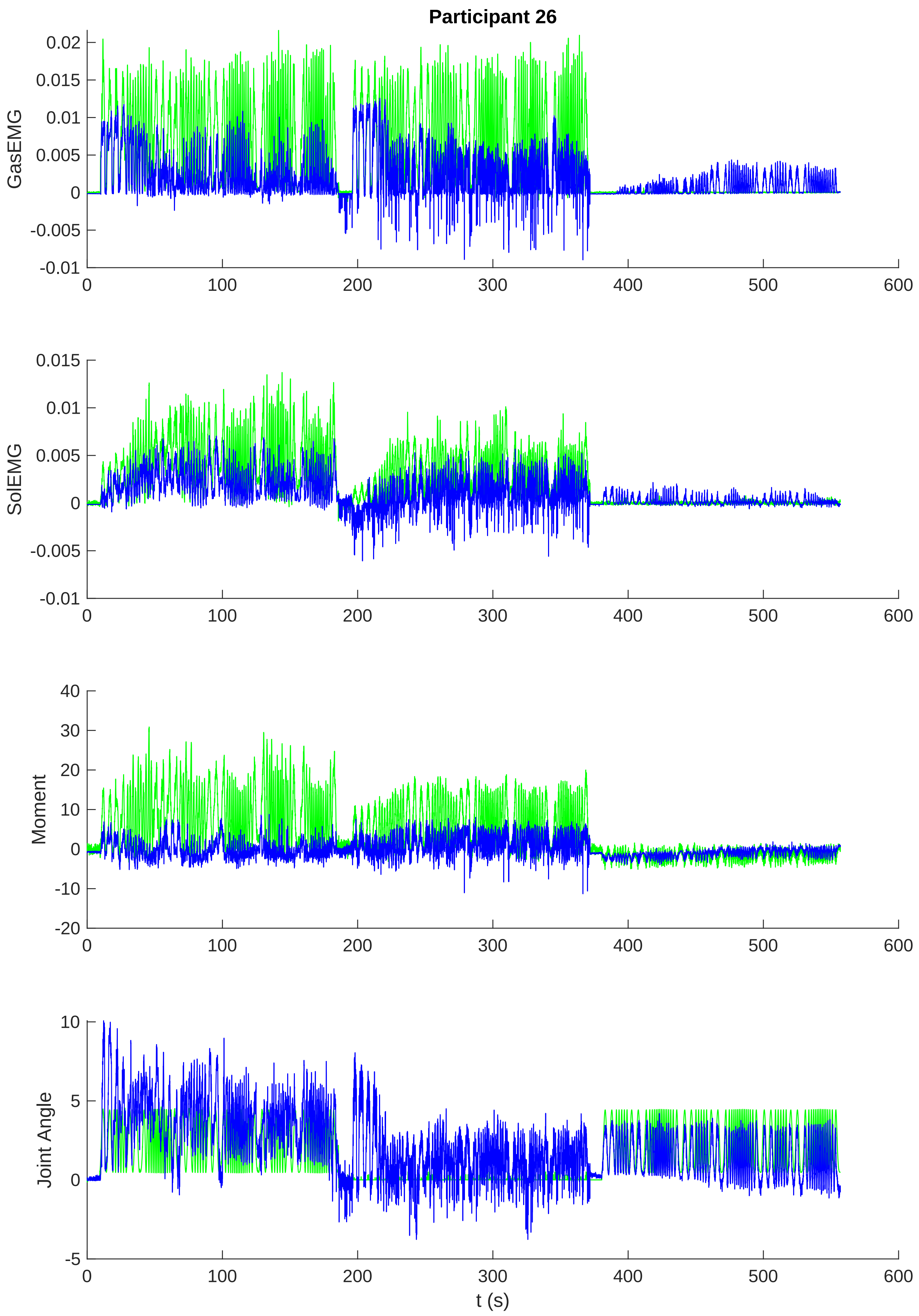
Participant 26
GasEMG
0.02
0.015
0.01
0.005
0
-0.005
-0.01
SolEMG
0.015
0.01
0.005
0
-0.005
-0.01
Moment
40
30
20
10
0
-10
-20
Joint Angle
10
5
0
-5
0
100
200
300
400
500
600
t (s)

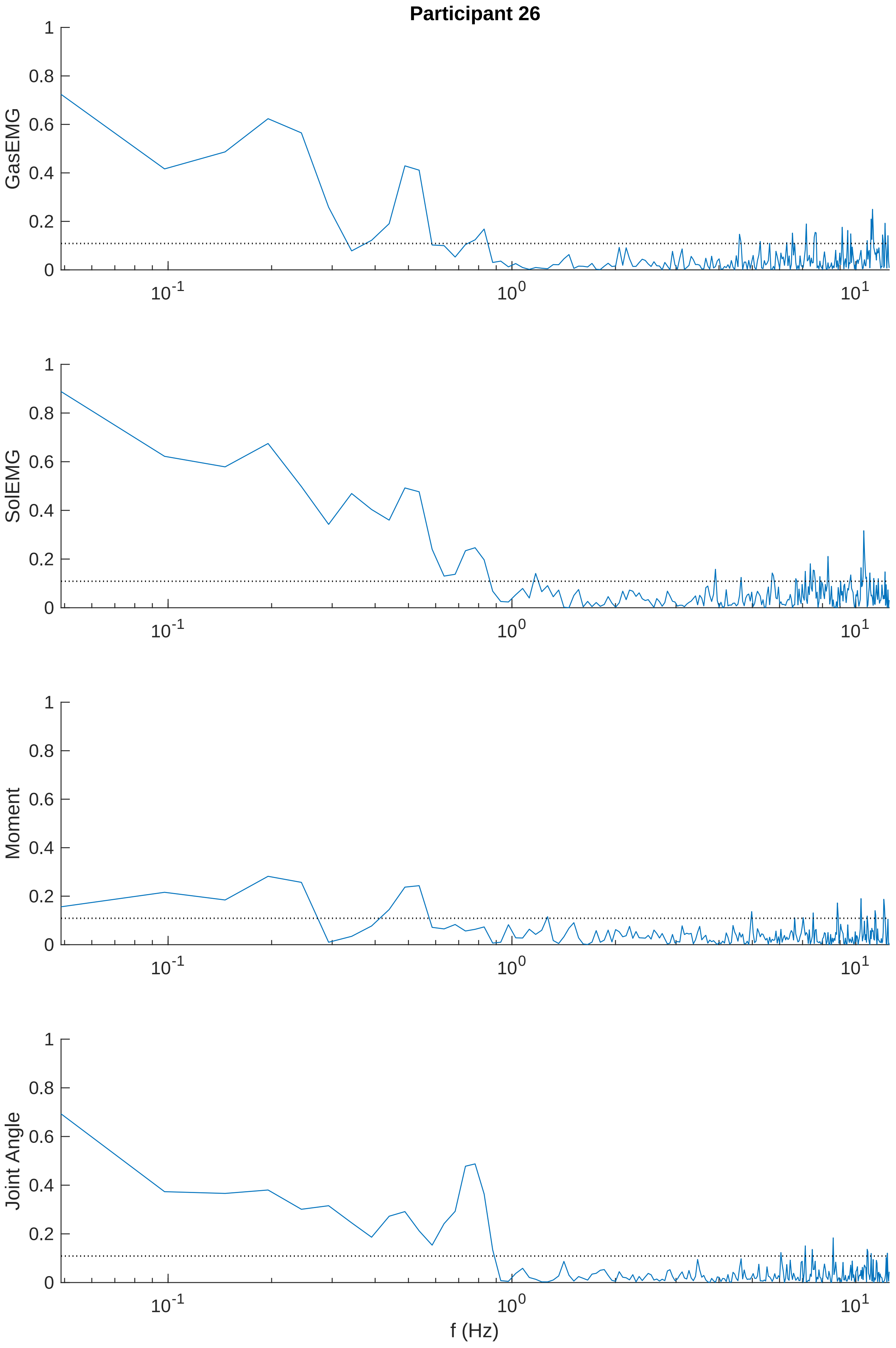

Participant 26
GasEMG
SolEMG
Moment
Joint Angle
f (Hz)

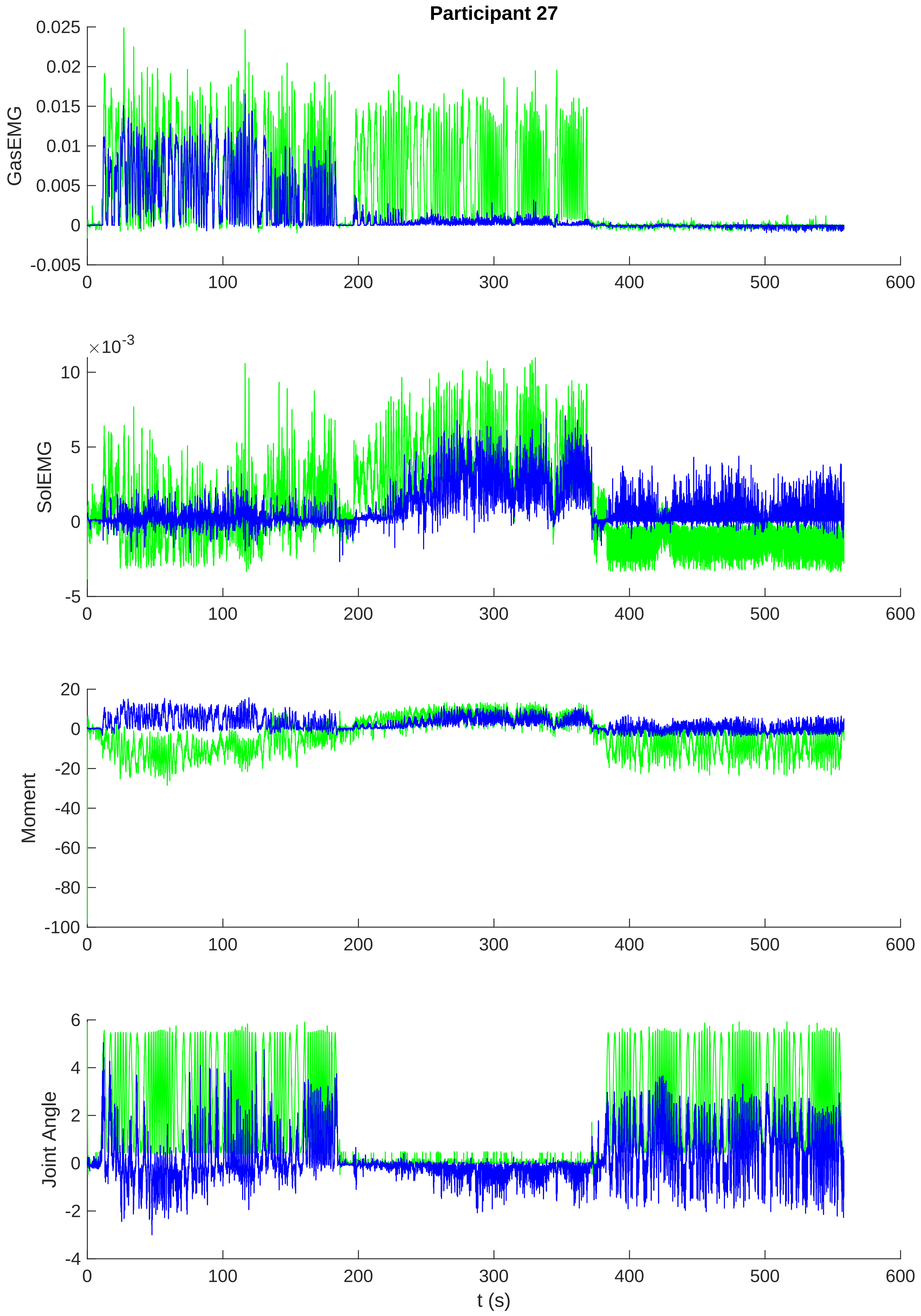

Participant 27
GasEMG
0.025
0.02
0.015
0.01
0.005
0
-0.005
0
100
200
300
400
500
600
×10^-3
SolEMG
10
5
0
-5
Moment
20
0
-20
-40
-60
-80
-100
Joint Angle
6
4
2
0
-2
-4
t (s)

Participant 27

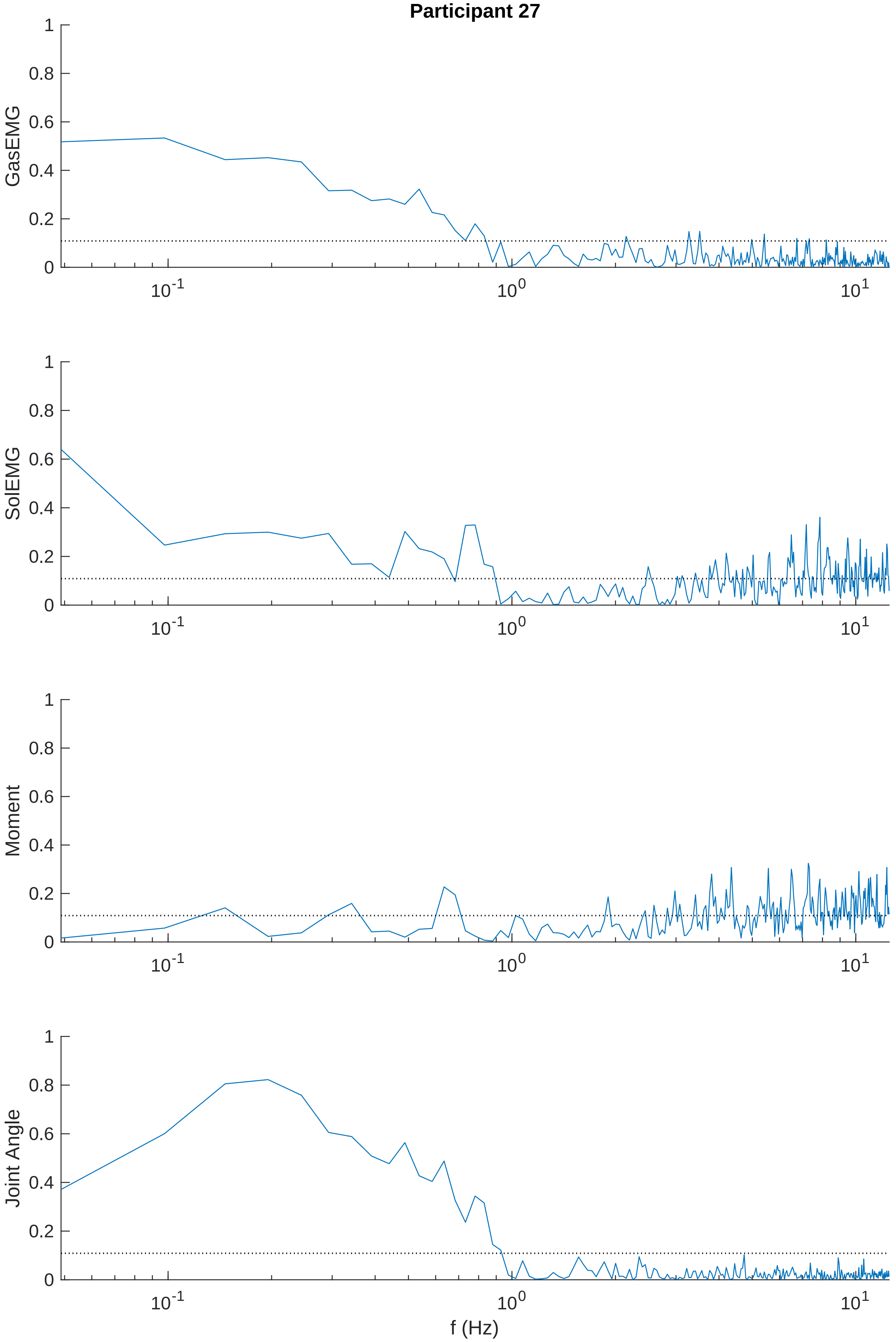

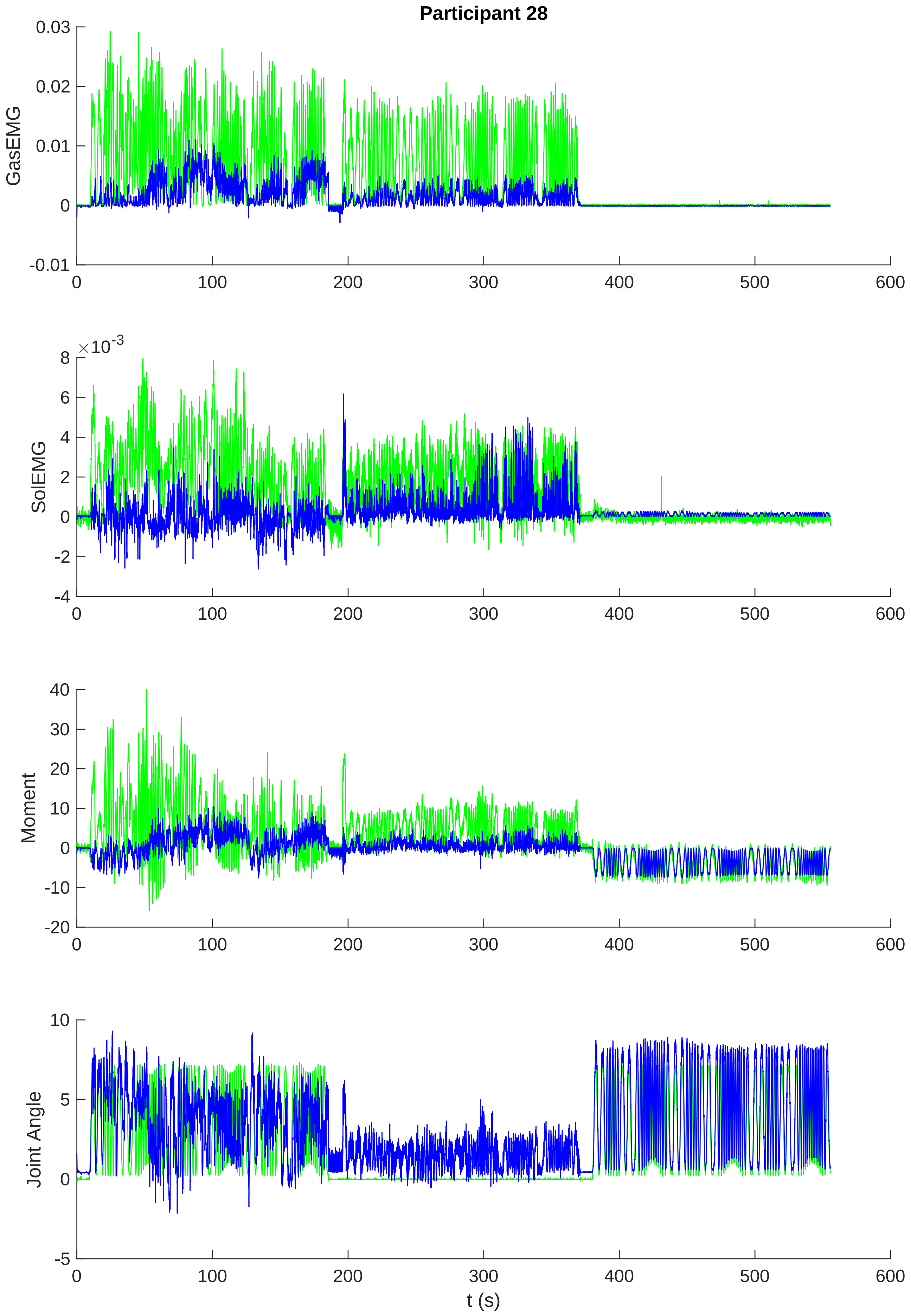

Participant 28
GasEMG
SolEMG
×10^-3
Moment
Joint Angle
t (s)

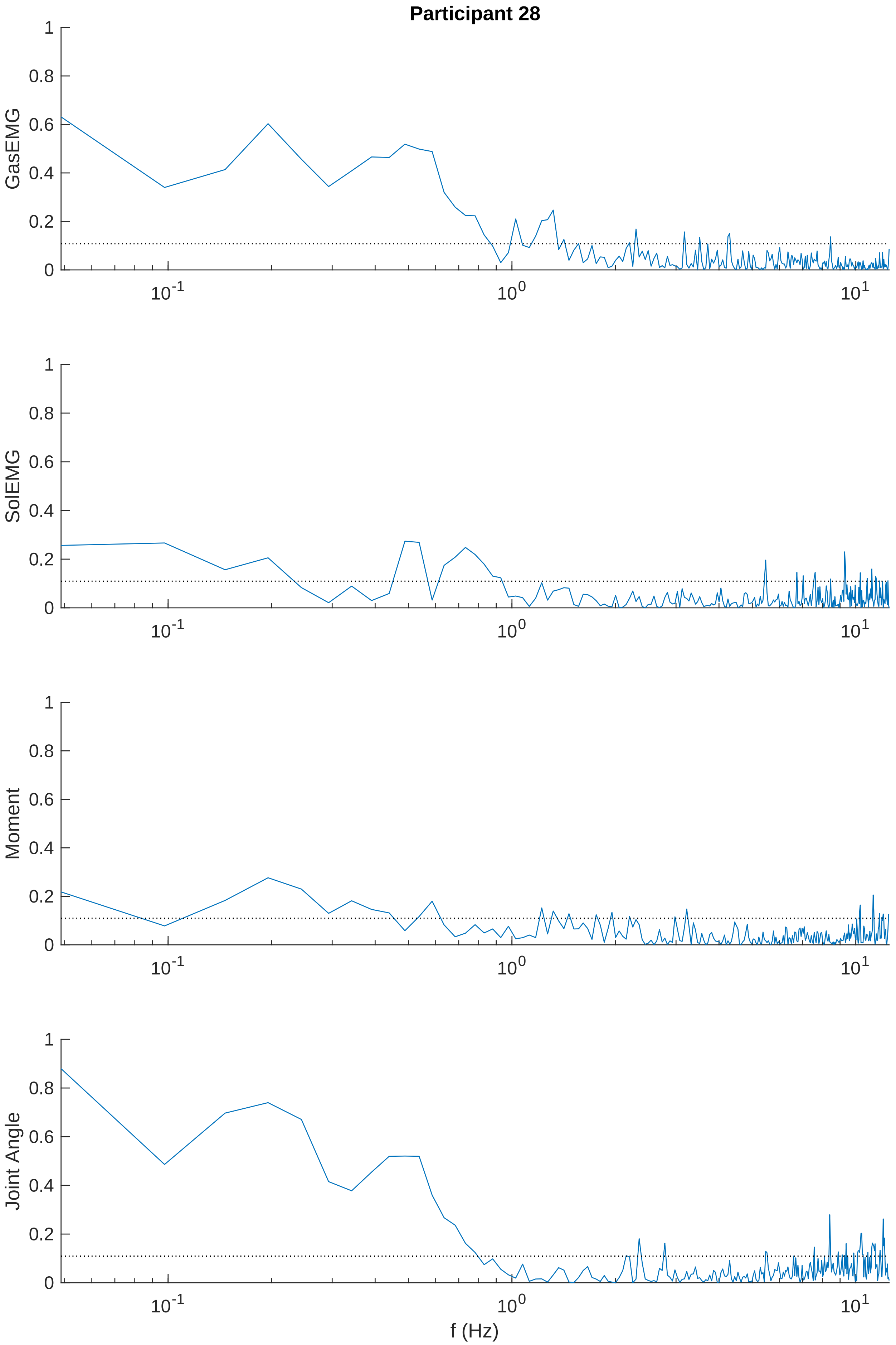
Participant 28
GasEMG
SolEMG
Moment
Joint Angle
f (Hz)
0
0.2
0.4
0.6
0.8
1
10^{-1}
10^{0}
10^{1}

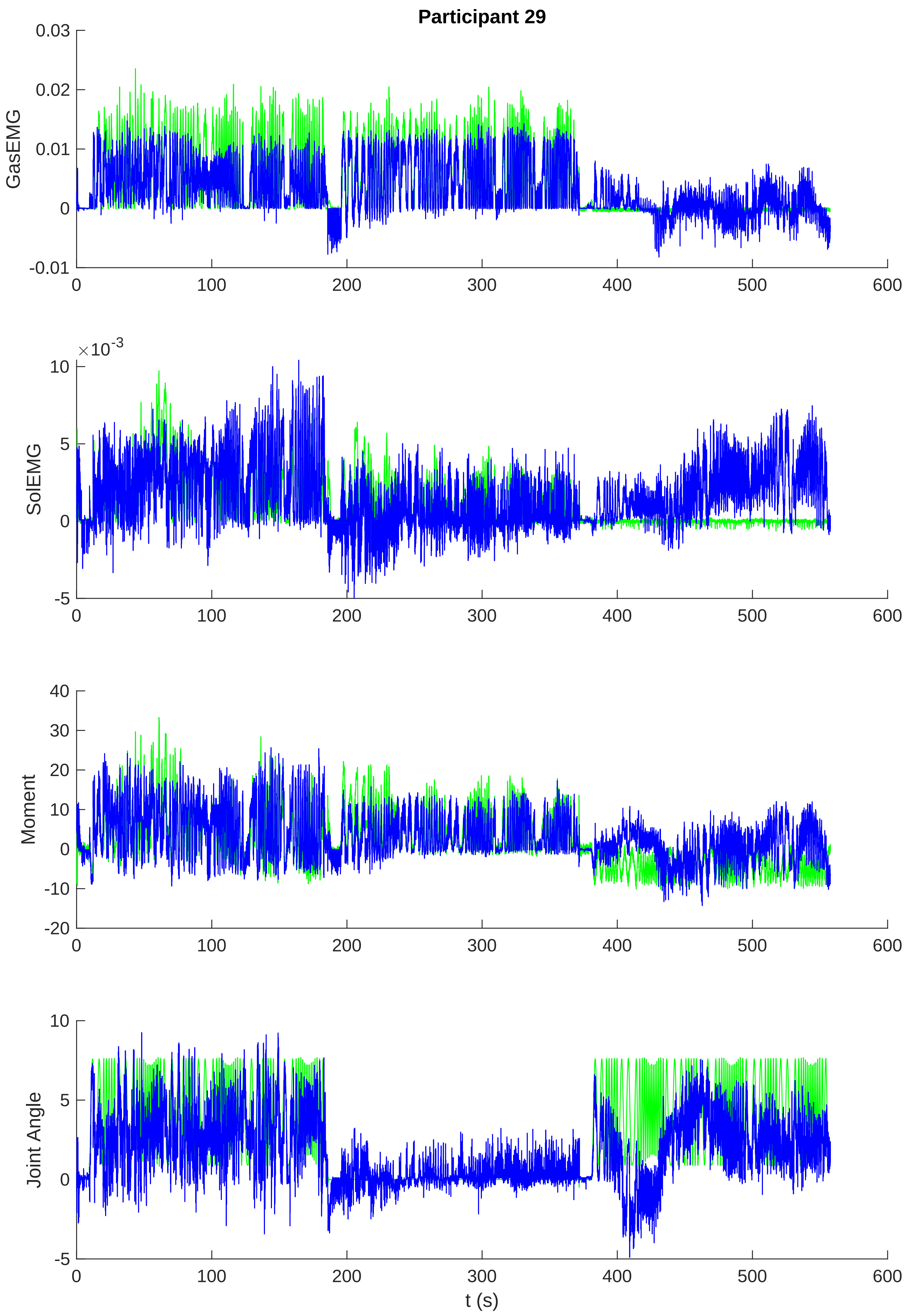

Participant 29
GasEMG
0.03
0.02
0.01
0
-0.01
0
100
200
300
400
500
600
SolEMG
×10^-3
10
5
0
-5
Moment
40
30
20
10
0
-10
-20
Joint Angle
10
5
0
-5
t (s)

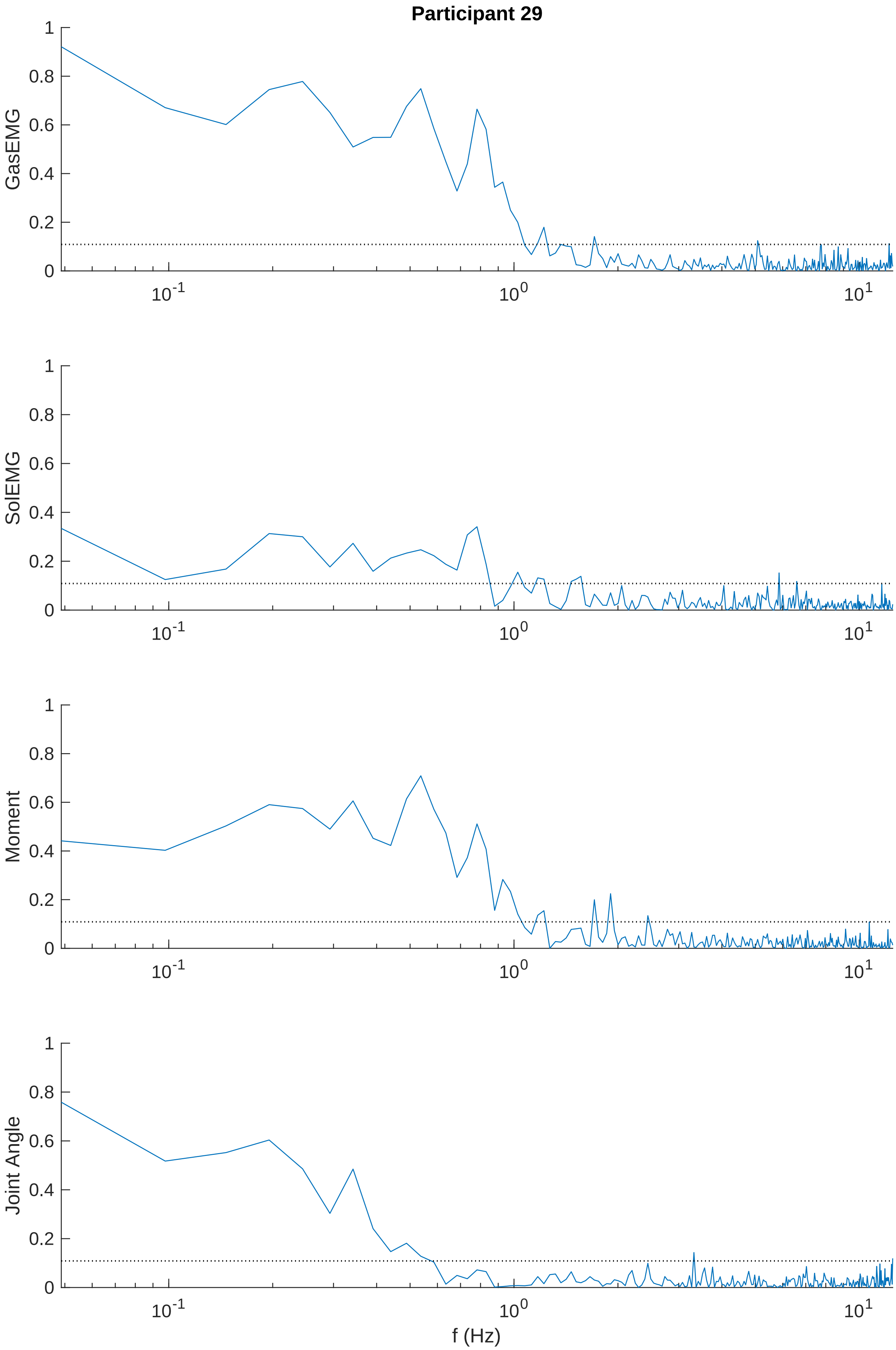
Participant 29
GasEMG
SolEMG
Moment
Joint Angle
f (Hz)
0
0.2
0.4
0.6
0.8
1
10^{-1}
10^{0}
10^{1}

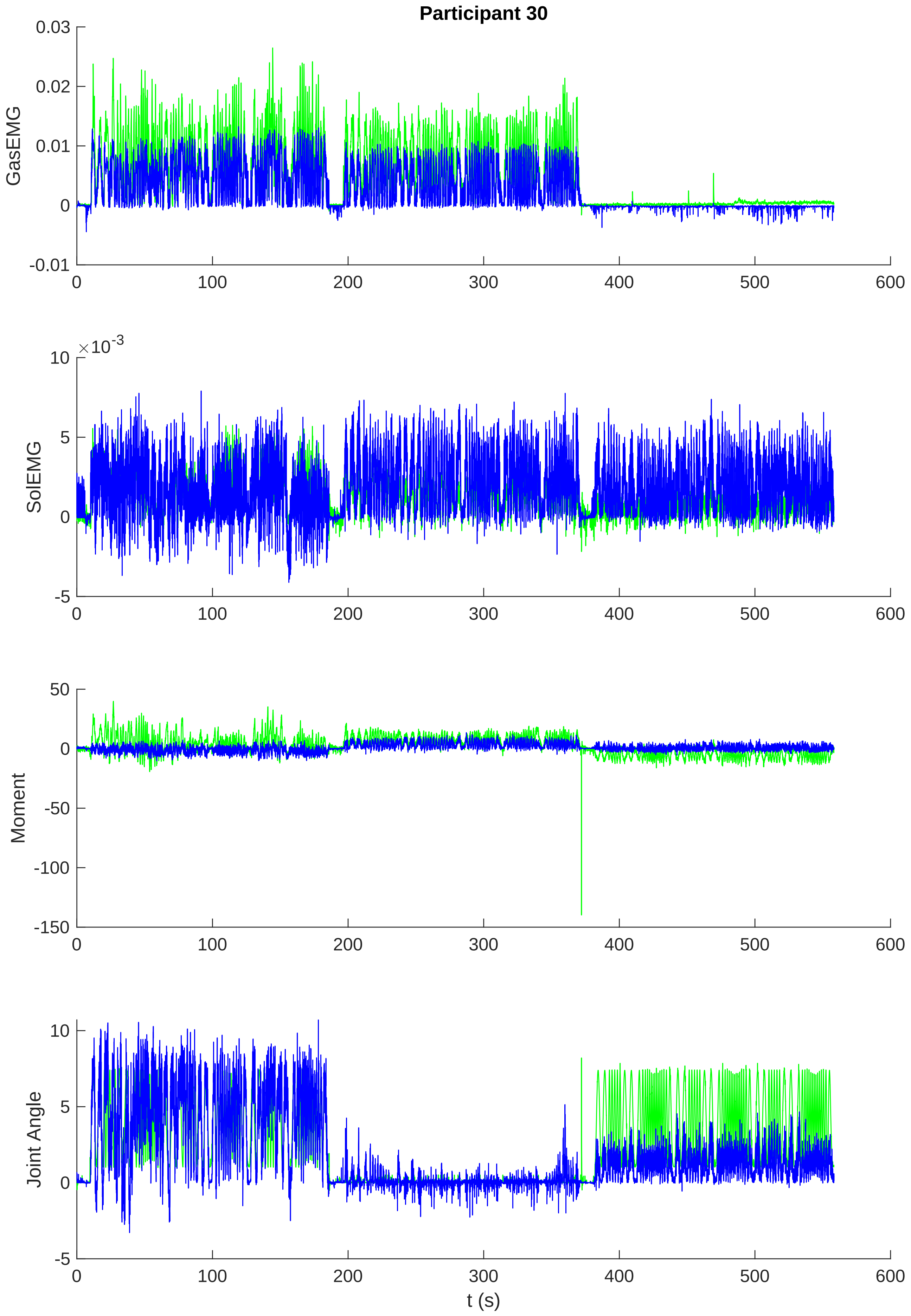
Participant 30
GasEMG
SolEMG
×10^-3
Moment
Joint Angle
t (s)
0.03
0.02
0.01
0
-0.01
10
5
-5
50
-50
-100
-150
100
200
300
400
500
600

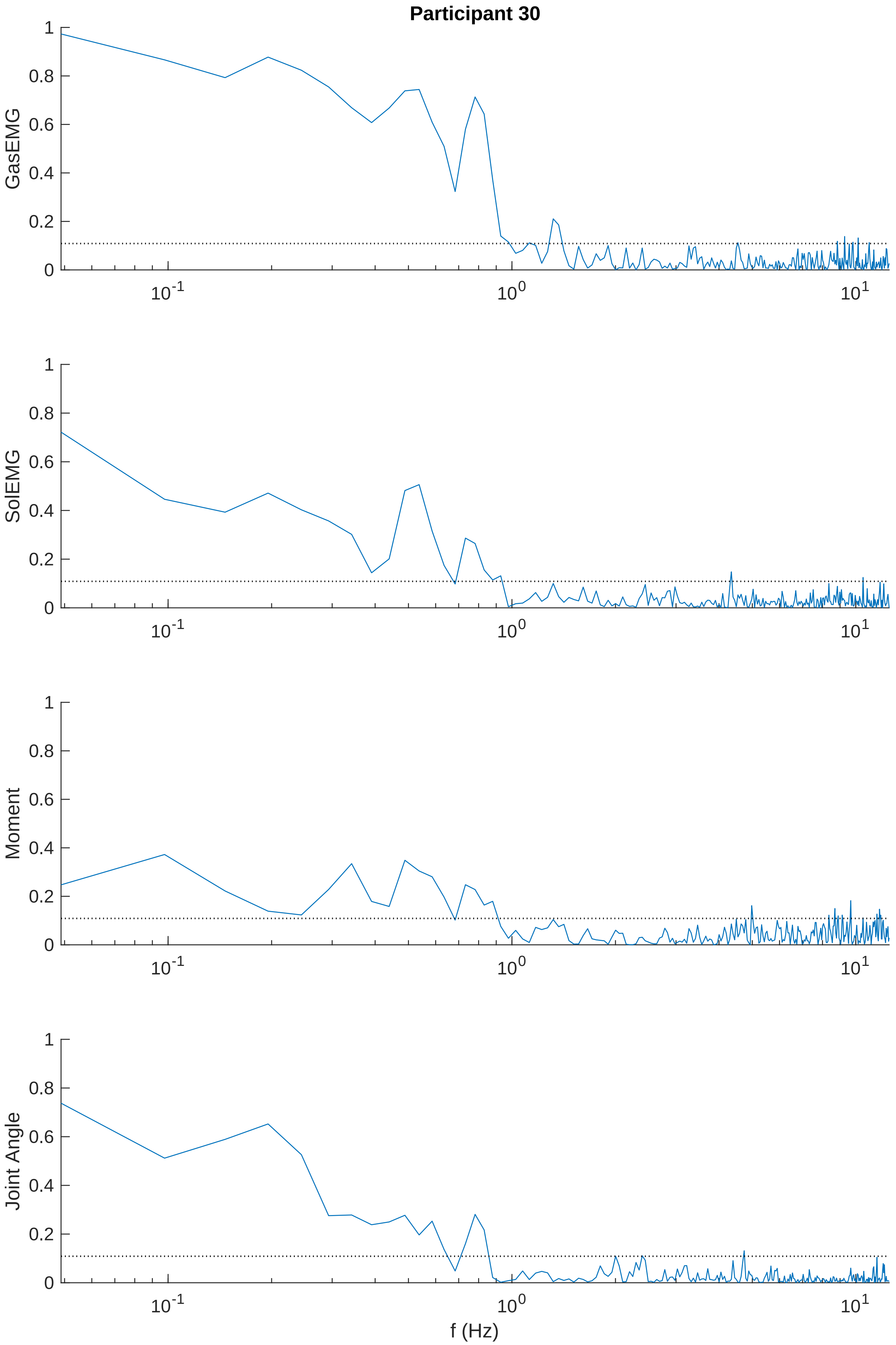

Participant 30
GasEMG
SolEMG
Moment
Joint Angle
f (Hz)

Participant 31

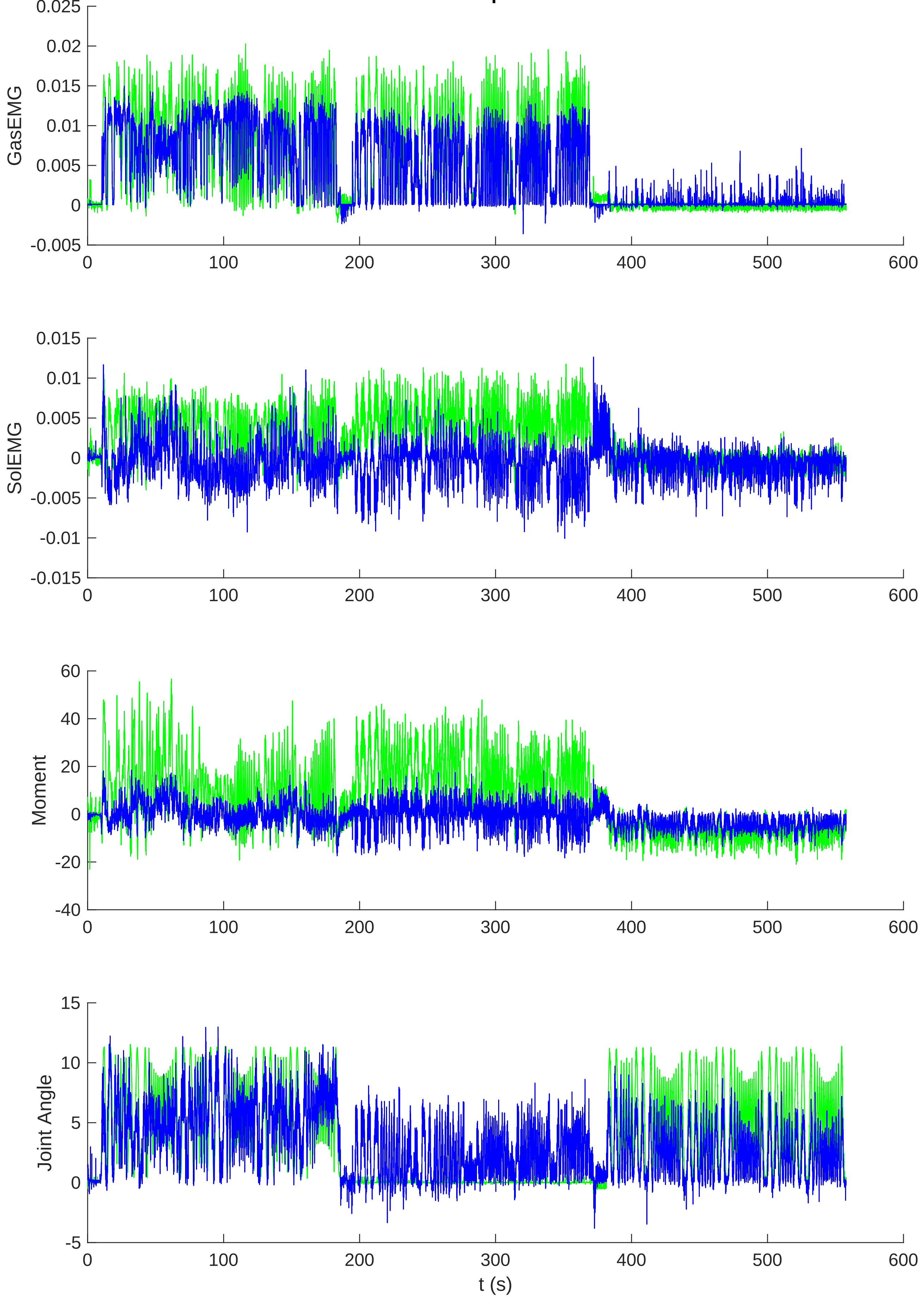

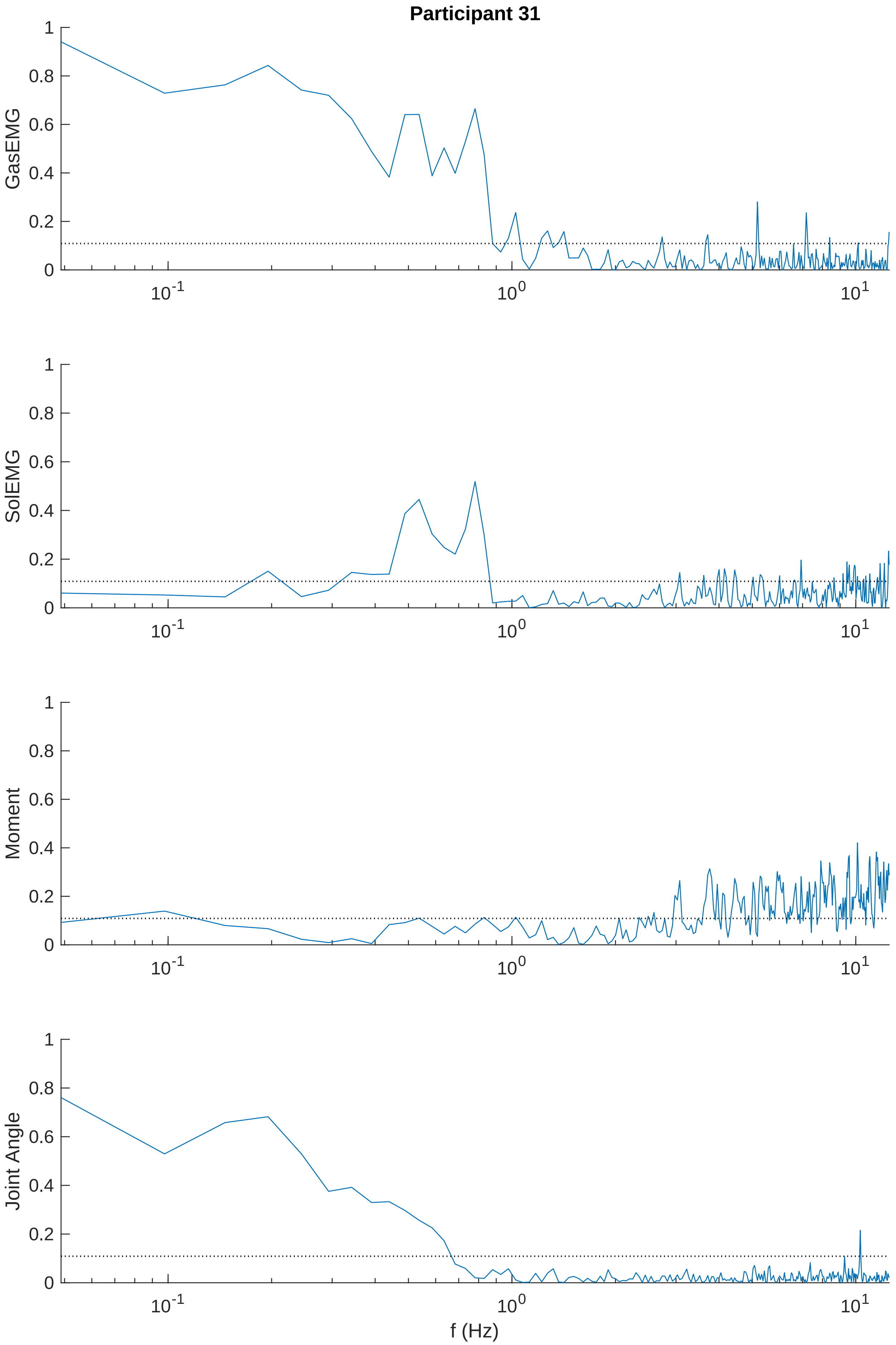

Participant 31
GasEMG
SolEMG
Moment
Joint Angle
f (Hz)
0
0.2
0.4
0.6
0.8
1
10^{-1}
10^{0}
10^{1}

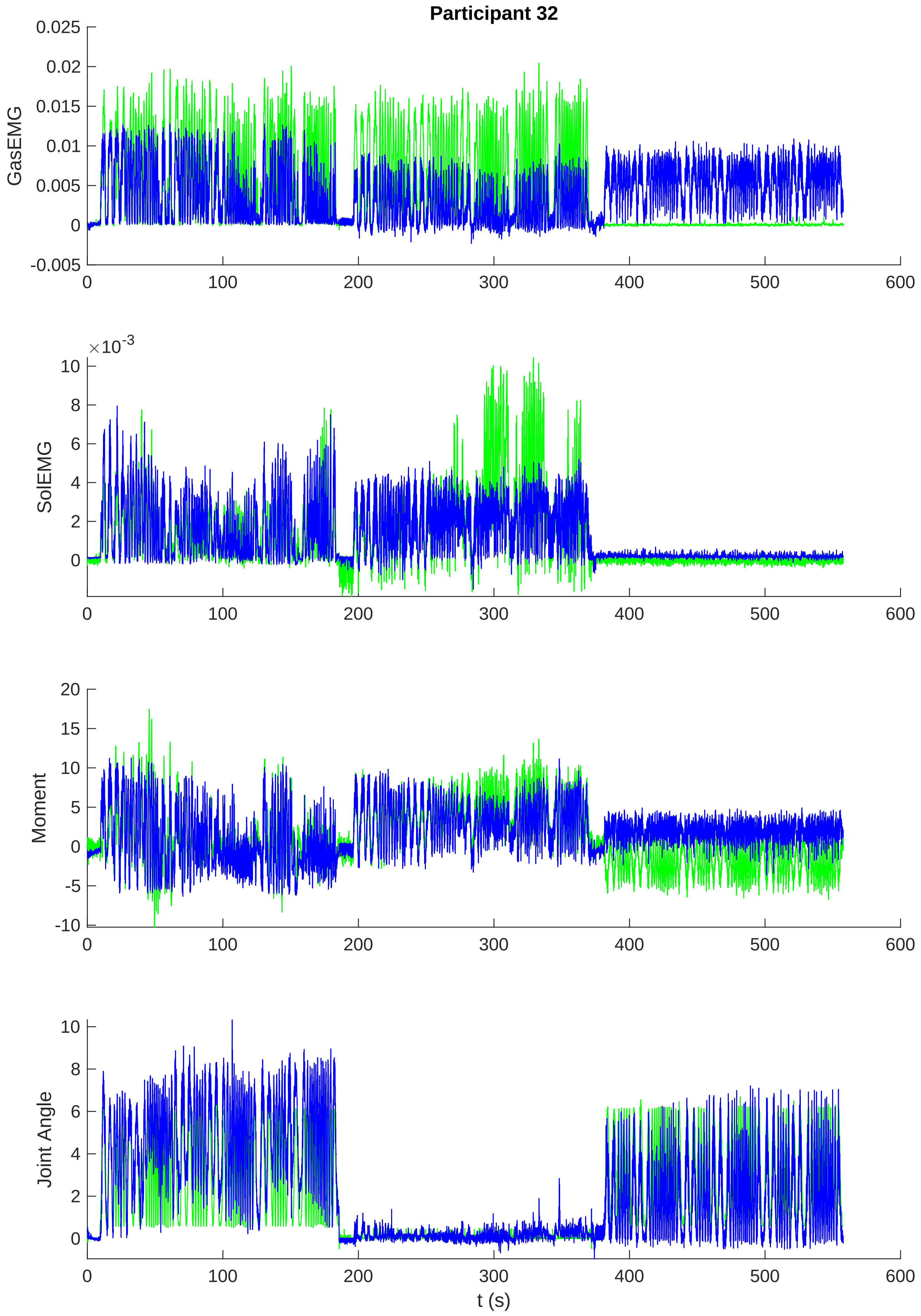

Participant 32
GasEMG
0.025
0.02
0.015
0.01
0.005
0
-0.005
0
100
200
300
400
500
600
SolEMG
×10^-3
10
8
6
4
2
0
Moment
20
15
10
5
0
-5
-10
Joint Angle
t (s)

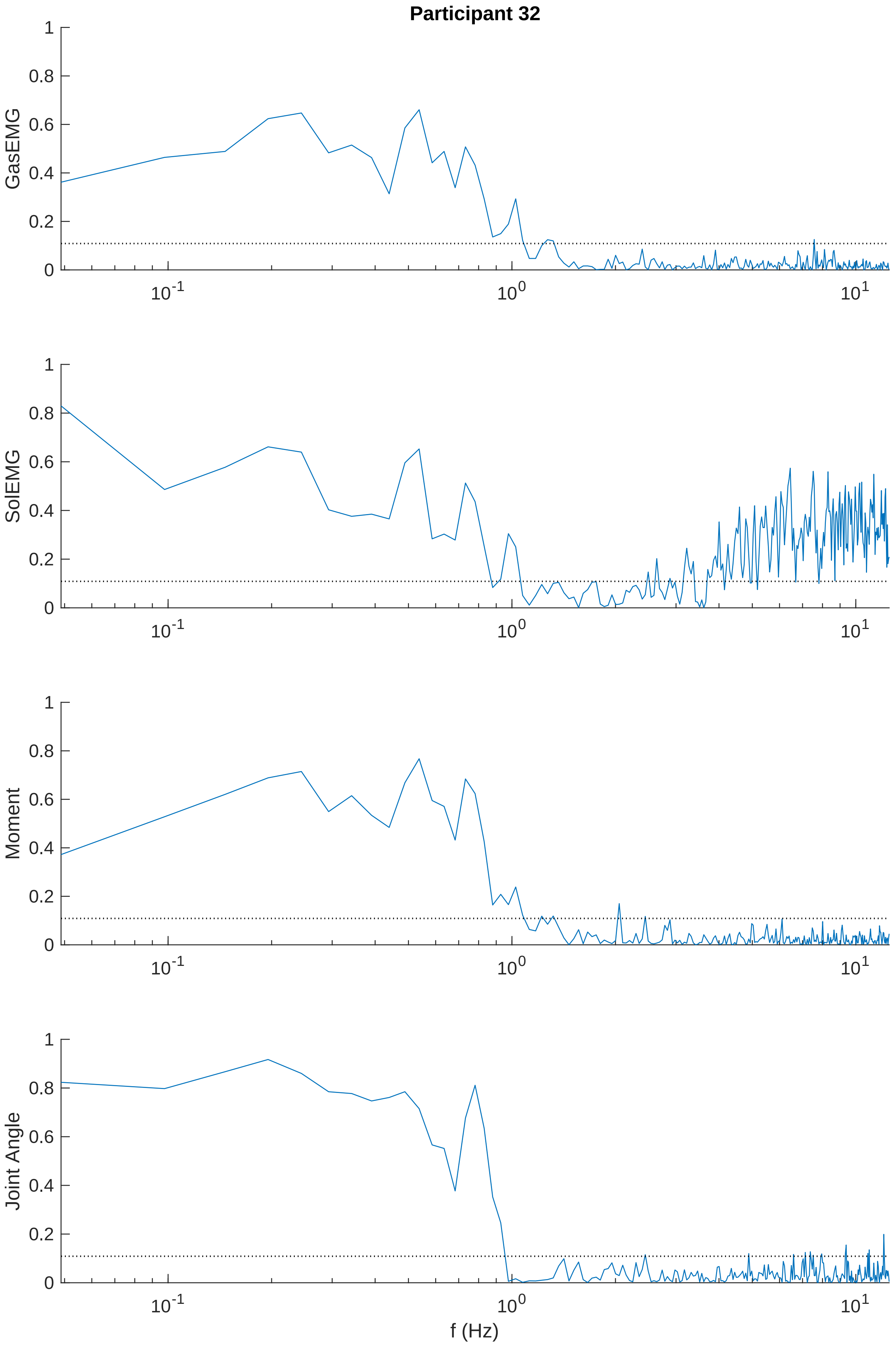
Participant 32
GasEMG
SolEMG
Moment
Joint Angle
1
0.8
0.6
0.4
0.2
0
10^{-1}
10^{0}
10^{1}
f (Hz)

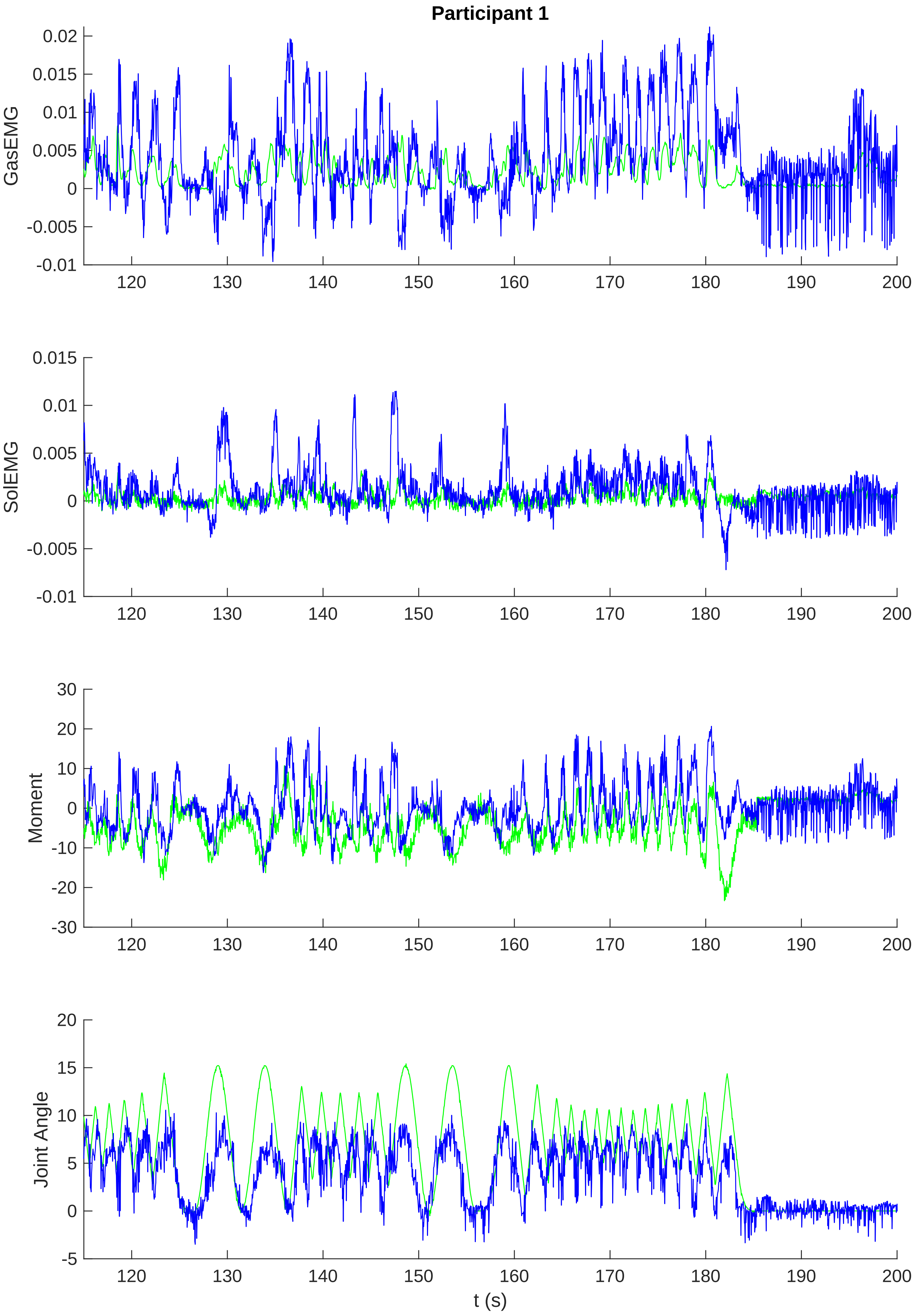

Participant 1
GasEMG
0.02
0.015
0.01
0.005
0
-0.005
-0.01
120
130
140
150
160
170
180
190
200
SolEMG
0.015
0.01
0.005
0
-0.005
-0.01
Moment
30
20
10
0
-10
-20
-30
Joint Angle
20
15
10
5
0
-5
t (s)

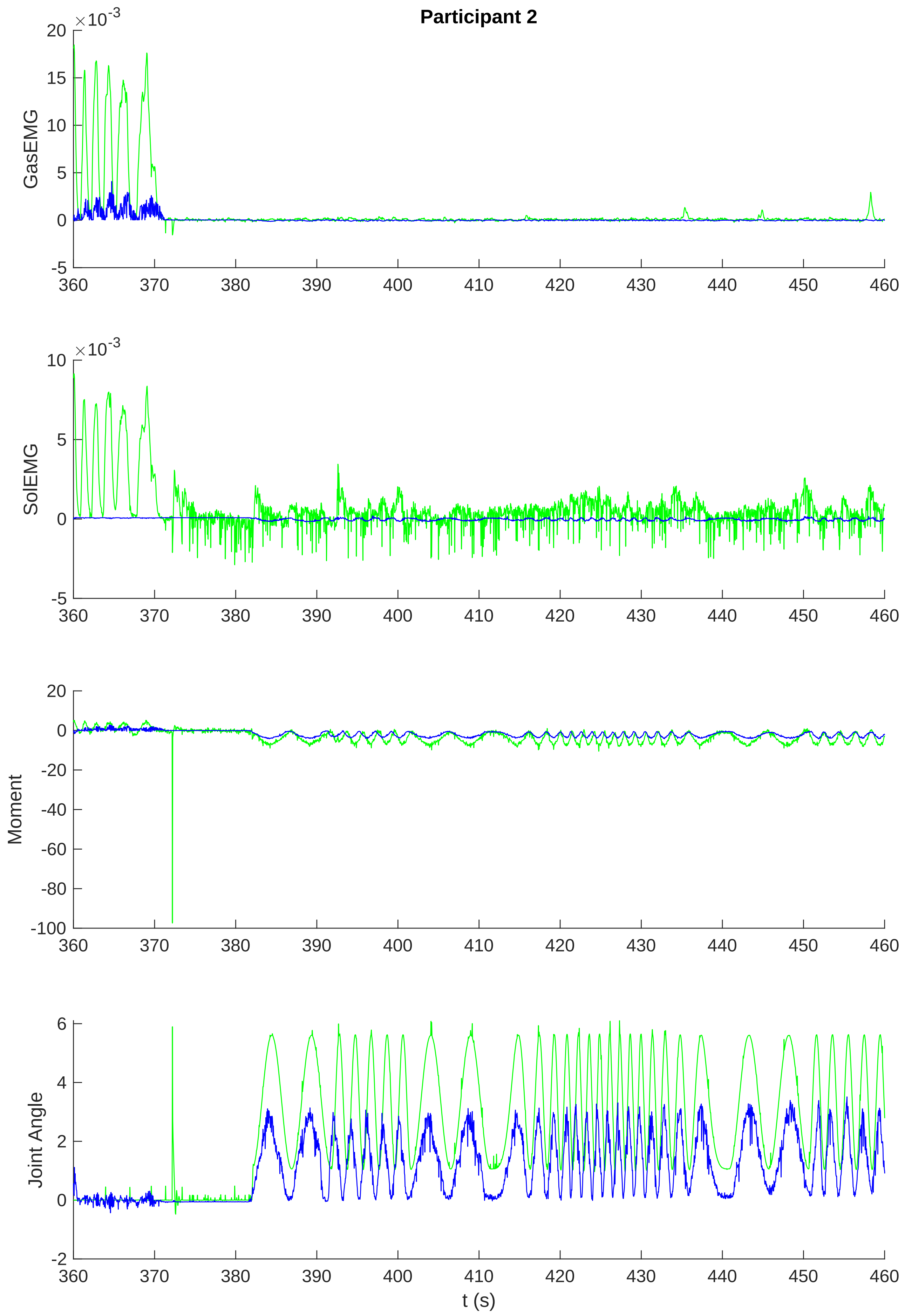

Participant 2
GasEMG
×10^-3
SolEMG
Moment
Joint Angle
t (s)

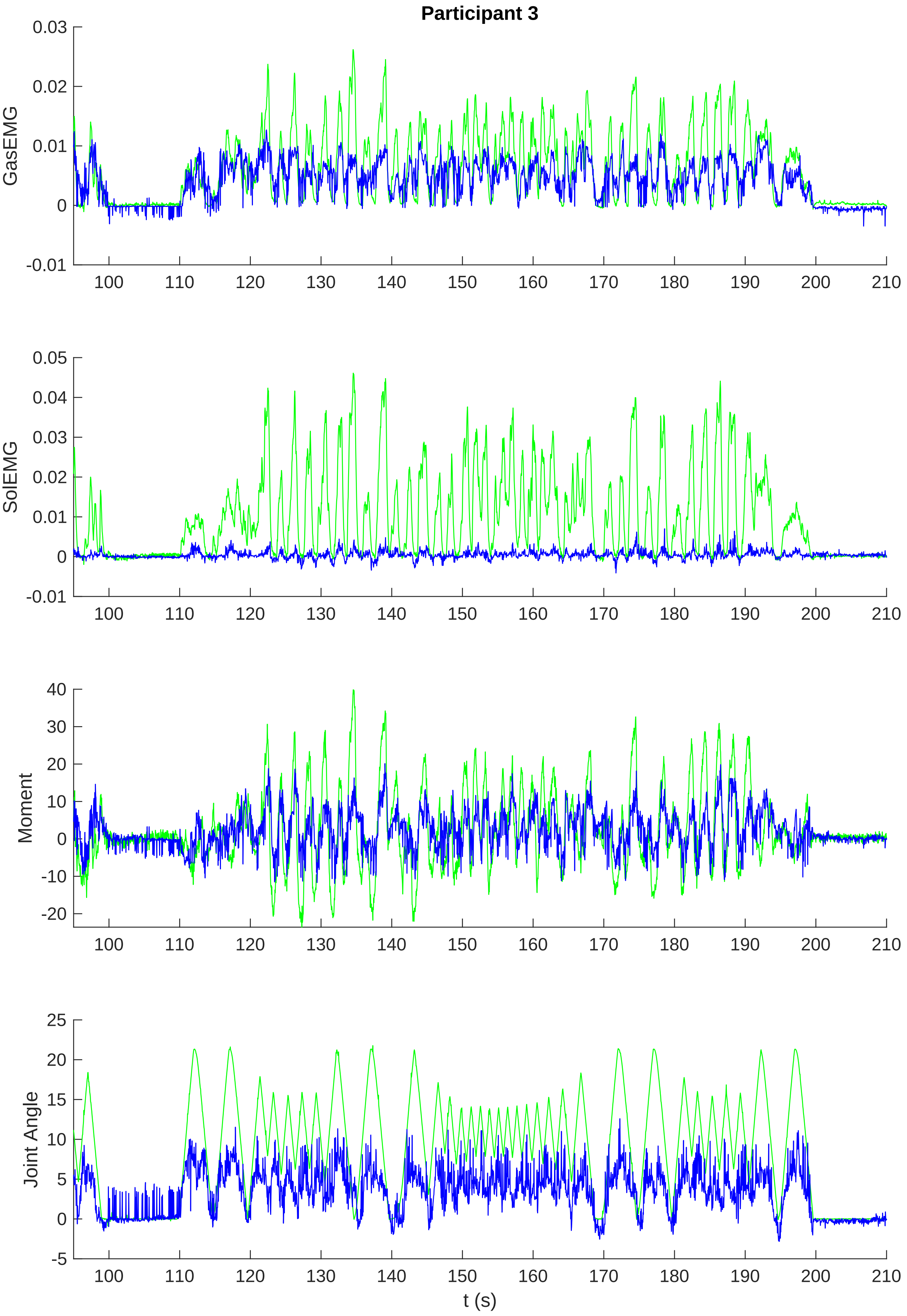

Participant 3
GasEMG
0.03
0.02
0.01
0
-0.01
SolEMG
0.05
0.04
0.03
0.02
0.01
0
-0.01
Moment
40
30
20
10
0
-10
-20
Joint Angle
25
20
15
10
5
0
-5
100
110
120
130
140
150
160
170
180
190
200
210
t (s)

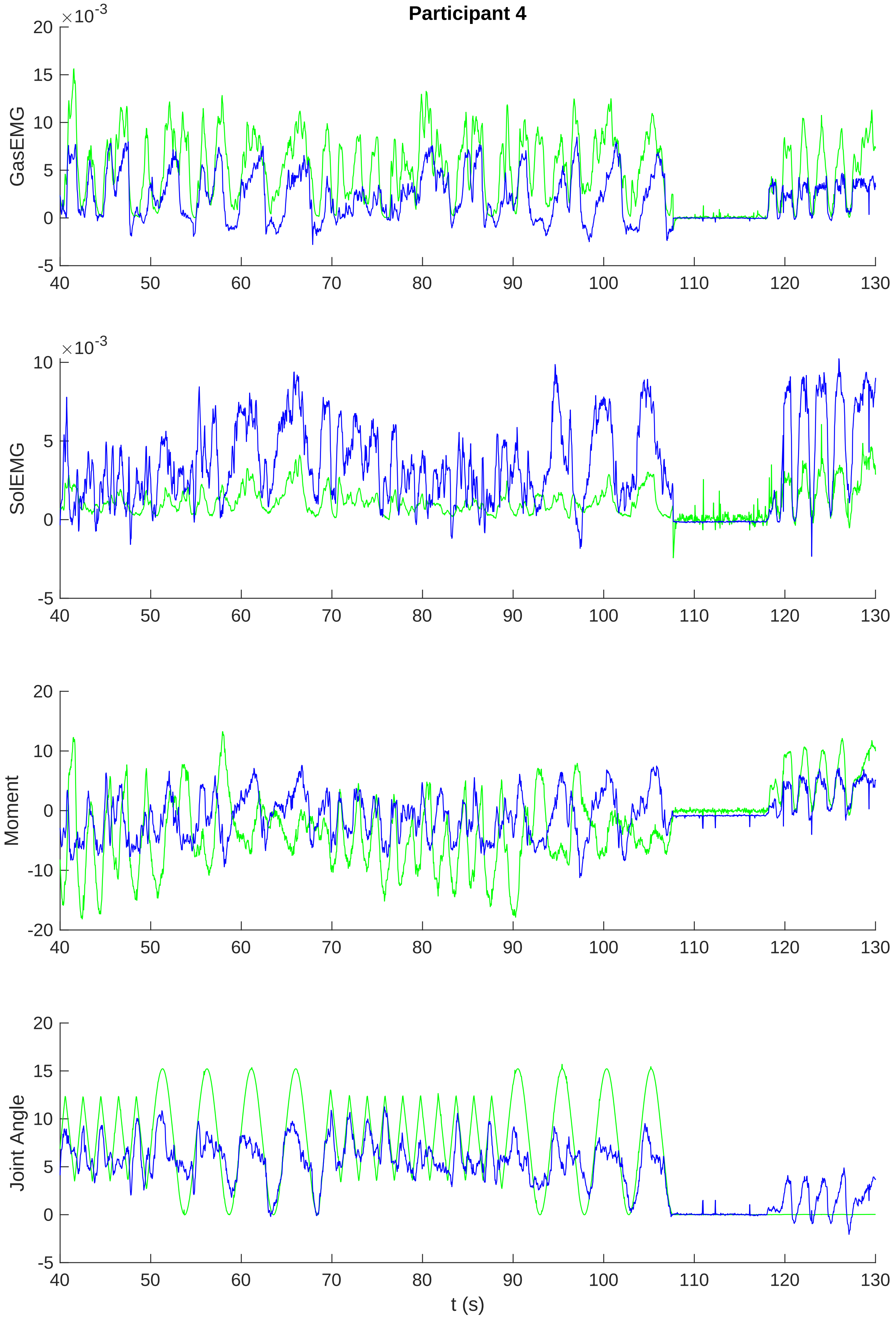
Participant 4
GasEMG
×10^-3
SolEMG
×10^-3
Moment
Joint Angle
t (s)
40
50
60
70
80
90
100
110
120
130

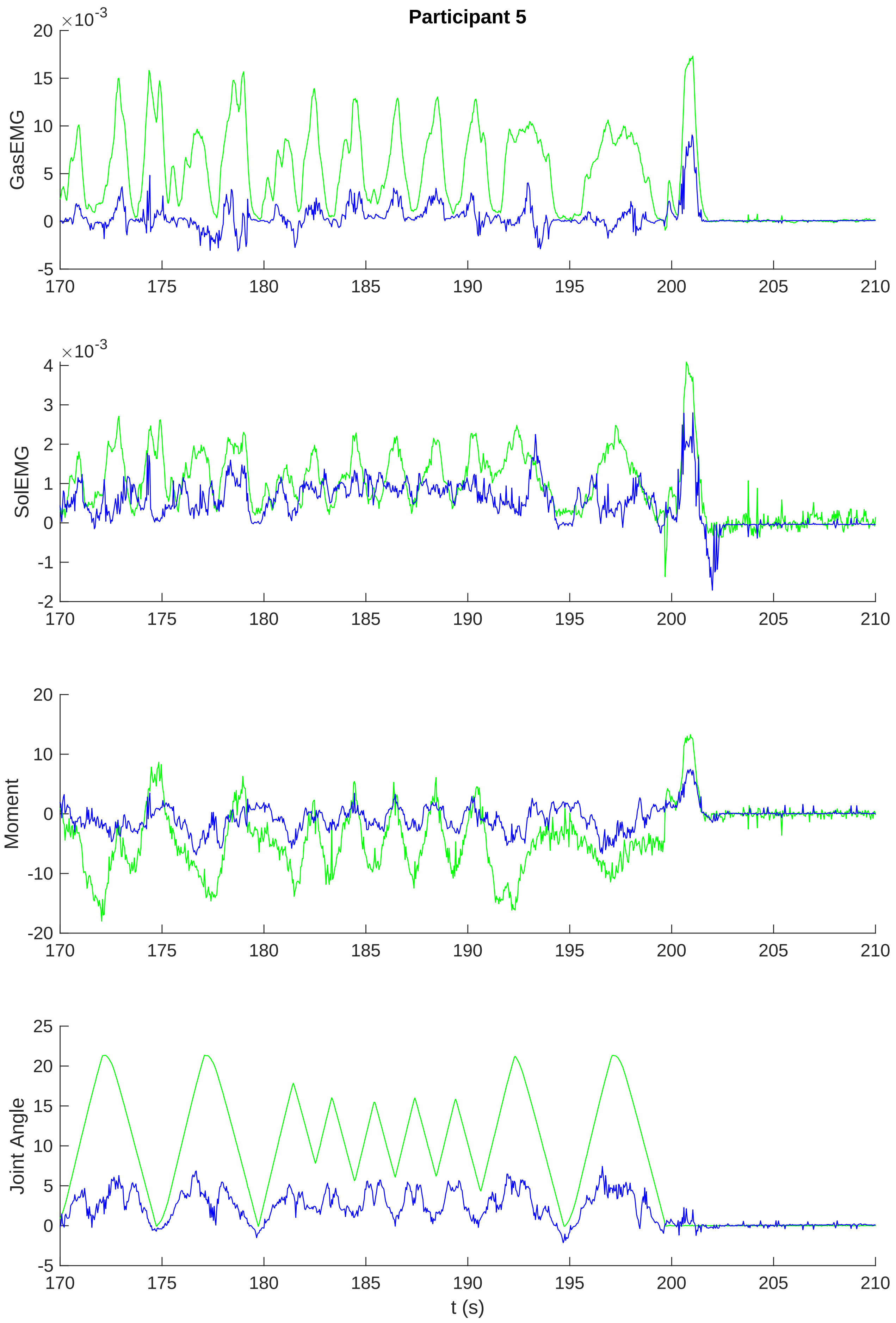

Participant 5
×10^-3
GasEMG
20
15
10
5
0
-5
×10^-3
SolEMG
4
3
2
1
0
-1
-2
Moment
20
10
0
-10
-20
Joint Angle
25
20
15
10
5
0
-5
170
175
180
185
190
195
200
205
210
t (s)

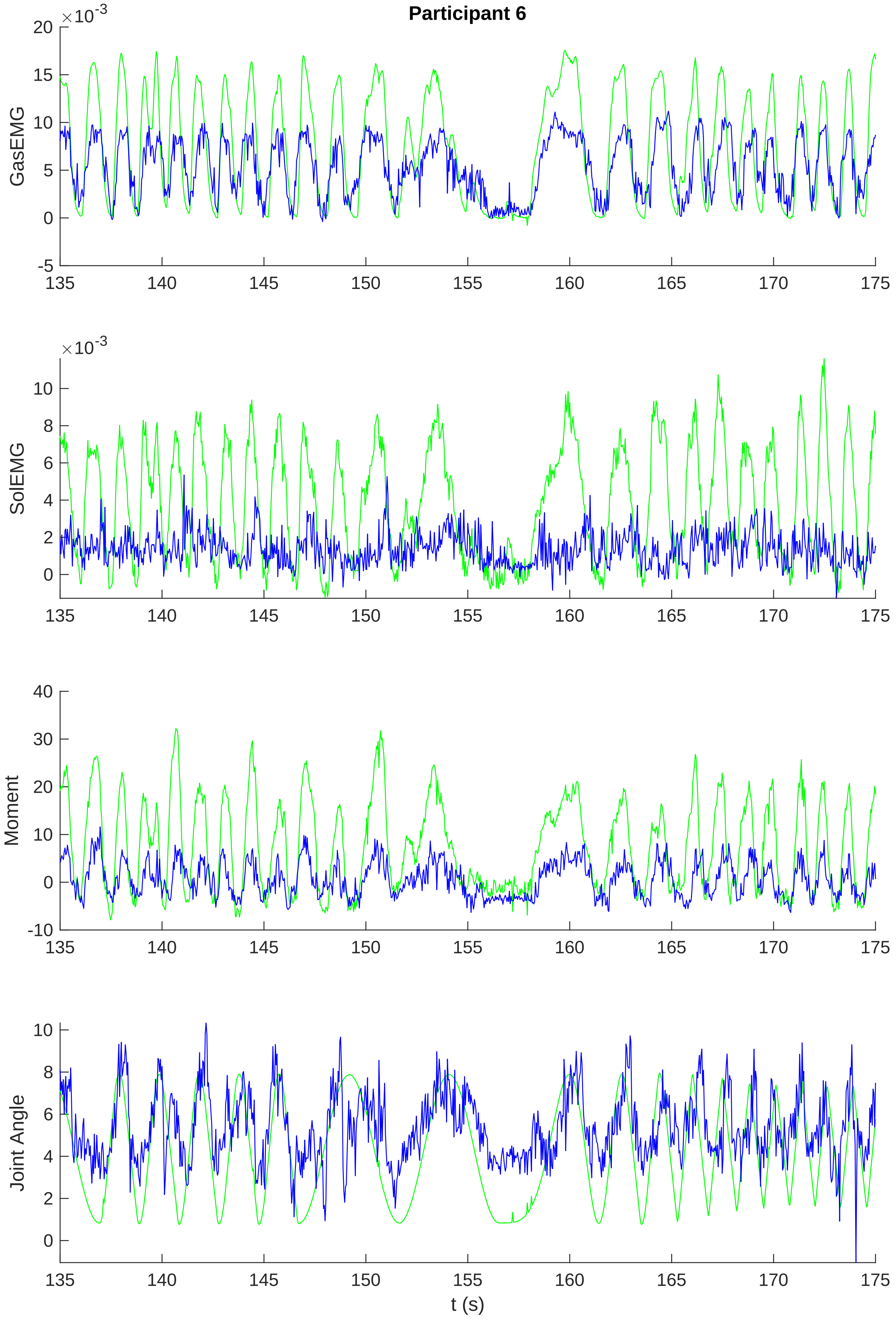
Participant 6
GasEMG
×10^-3
SolEMG
×10^-3
Moment
Joint Angle
t (s)

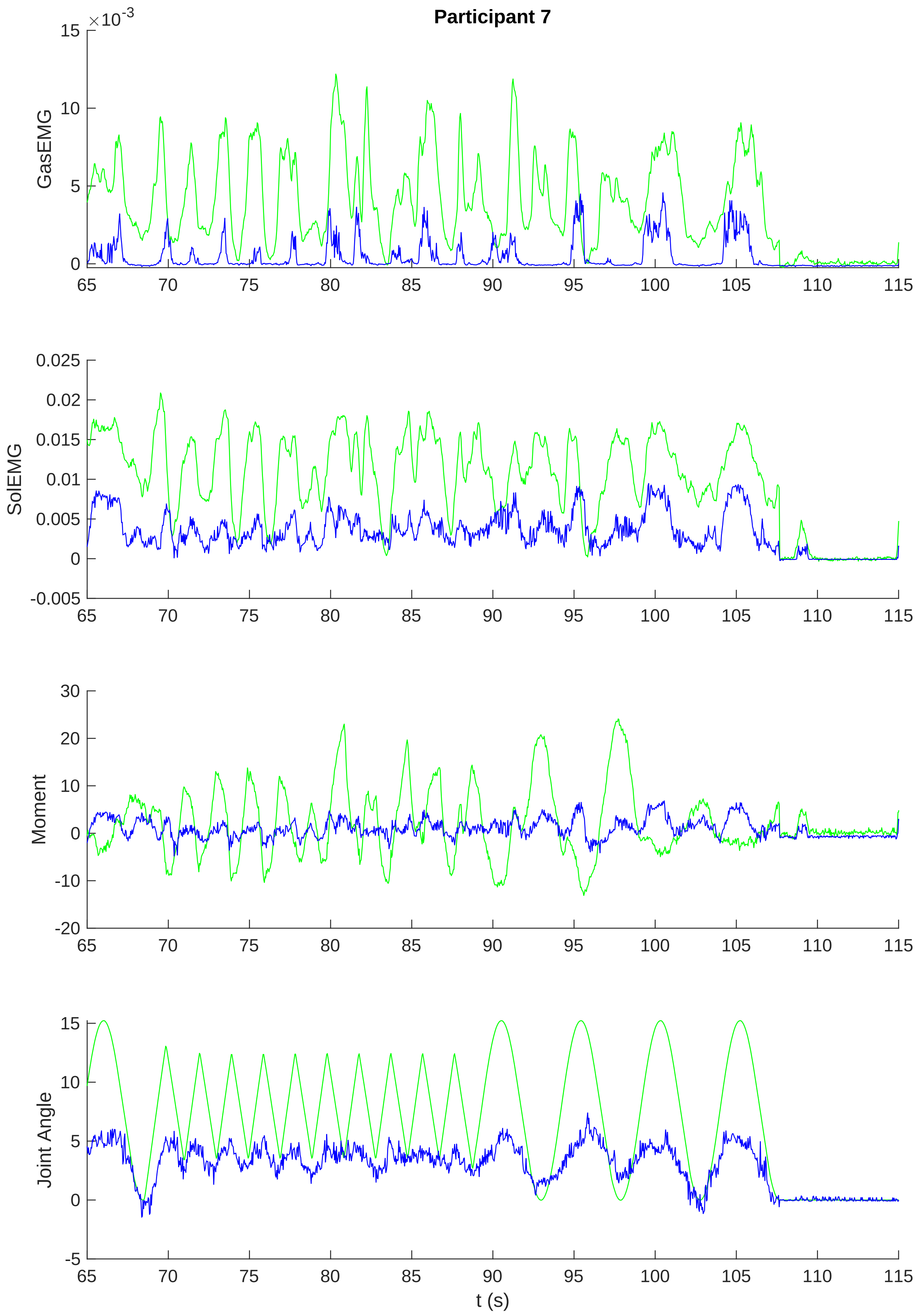

Participant 7
GasEMG
×10^-3
15
10
5
0
SolEMG
0.025
0.02
0.015
0.01
0.005
0
-0.005
Moment
30
20
10
0
-10
-20
Joint Angle
15
10
5
0
-5
65
70
75
80
85
90
95
100
105
110
115
t (s)

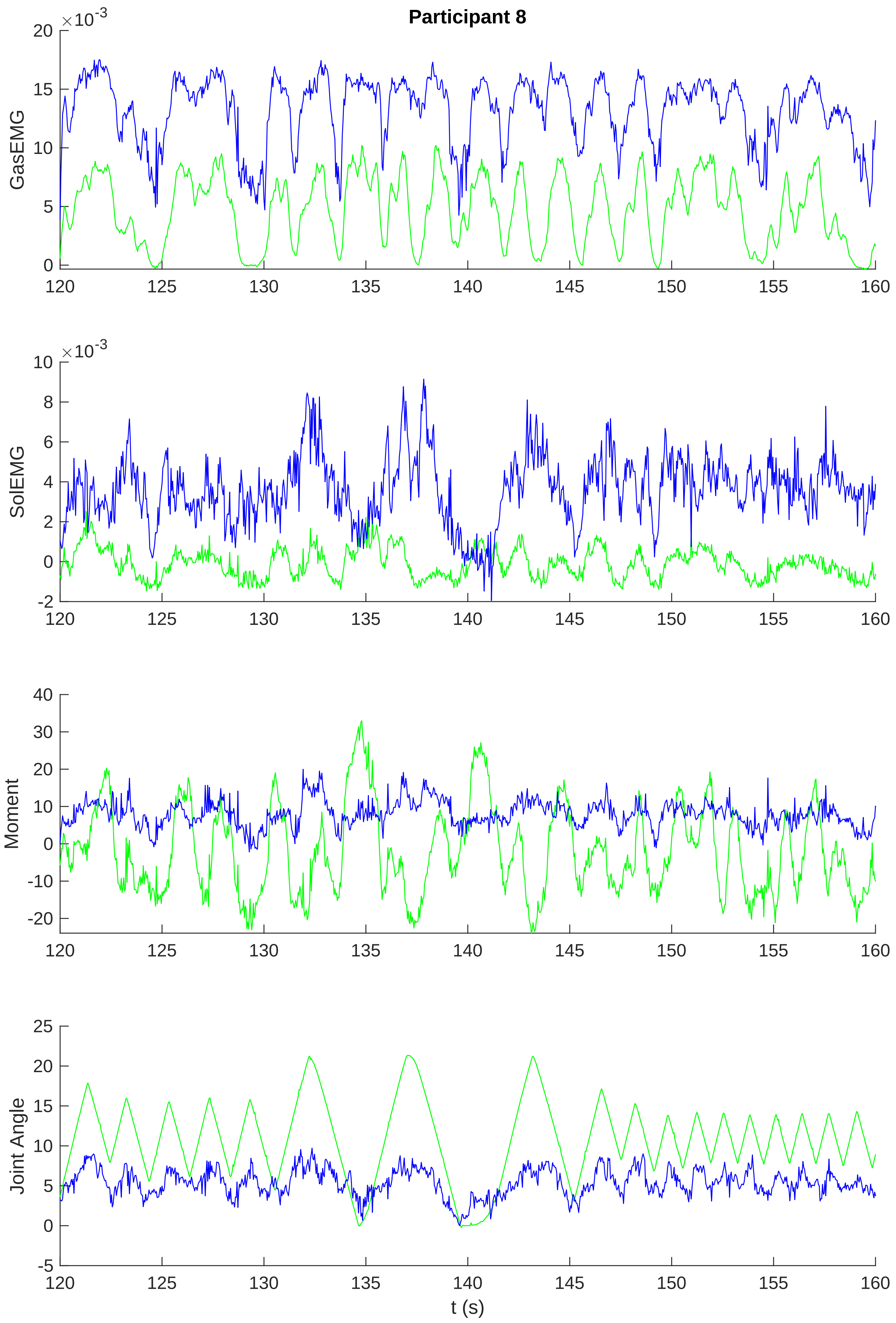

Participant 8
×10^-3
GasEMG
20
15
10
5
0
120
125
130
135
140
145
150
155
160
×10^-3
SolEMG
10
8
6
4
2
0
-2
Moment
40
30
20
10
0
-10
-20
Joint Angle
25
20
15
10
5
0
-5
t (s)

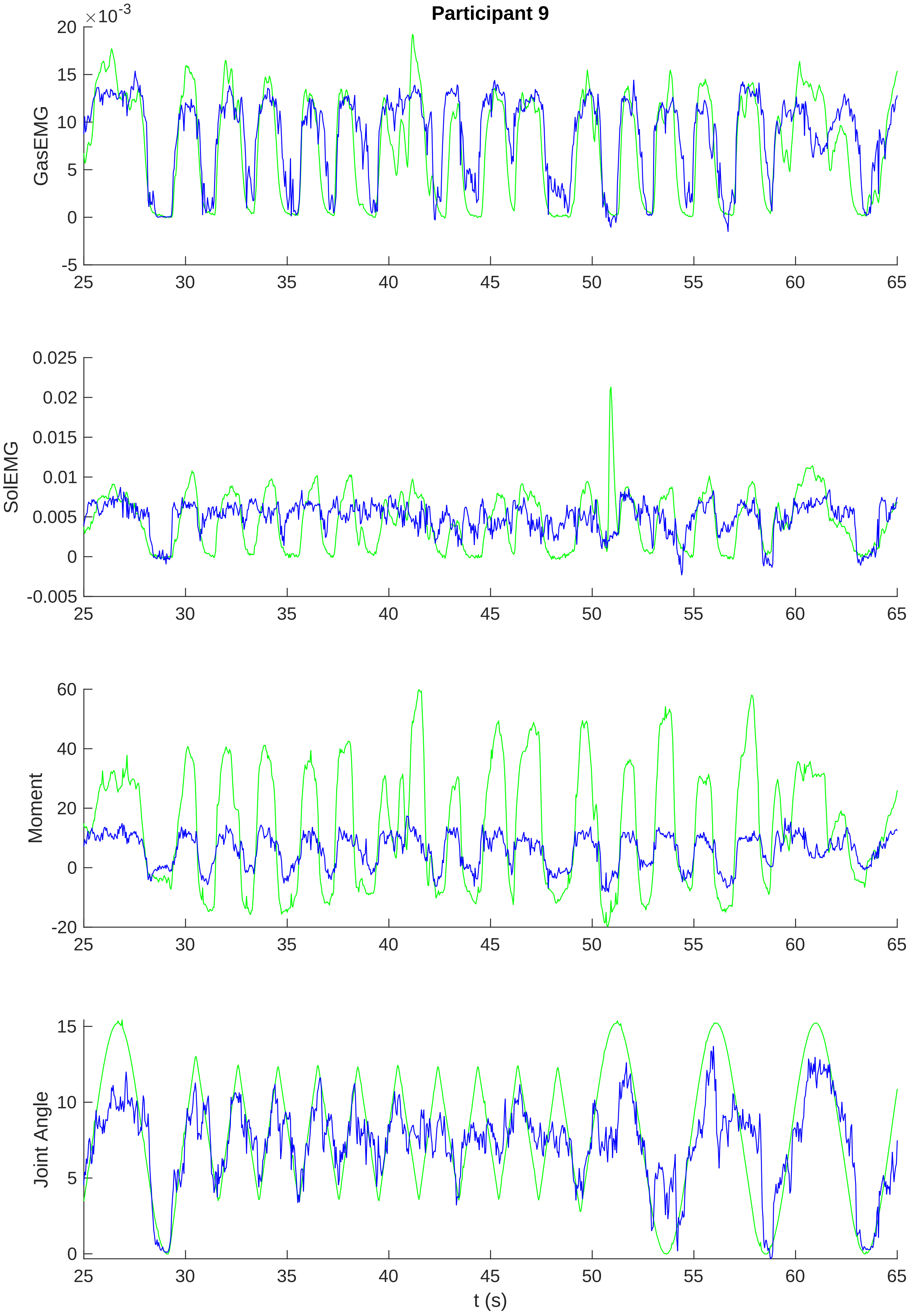

Participant 9
×10^-3
GasEMG
SolEMG
Moment
Joint Angle
t (s)

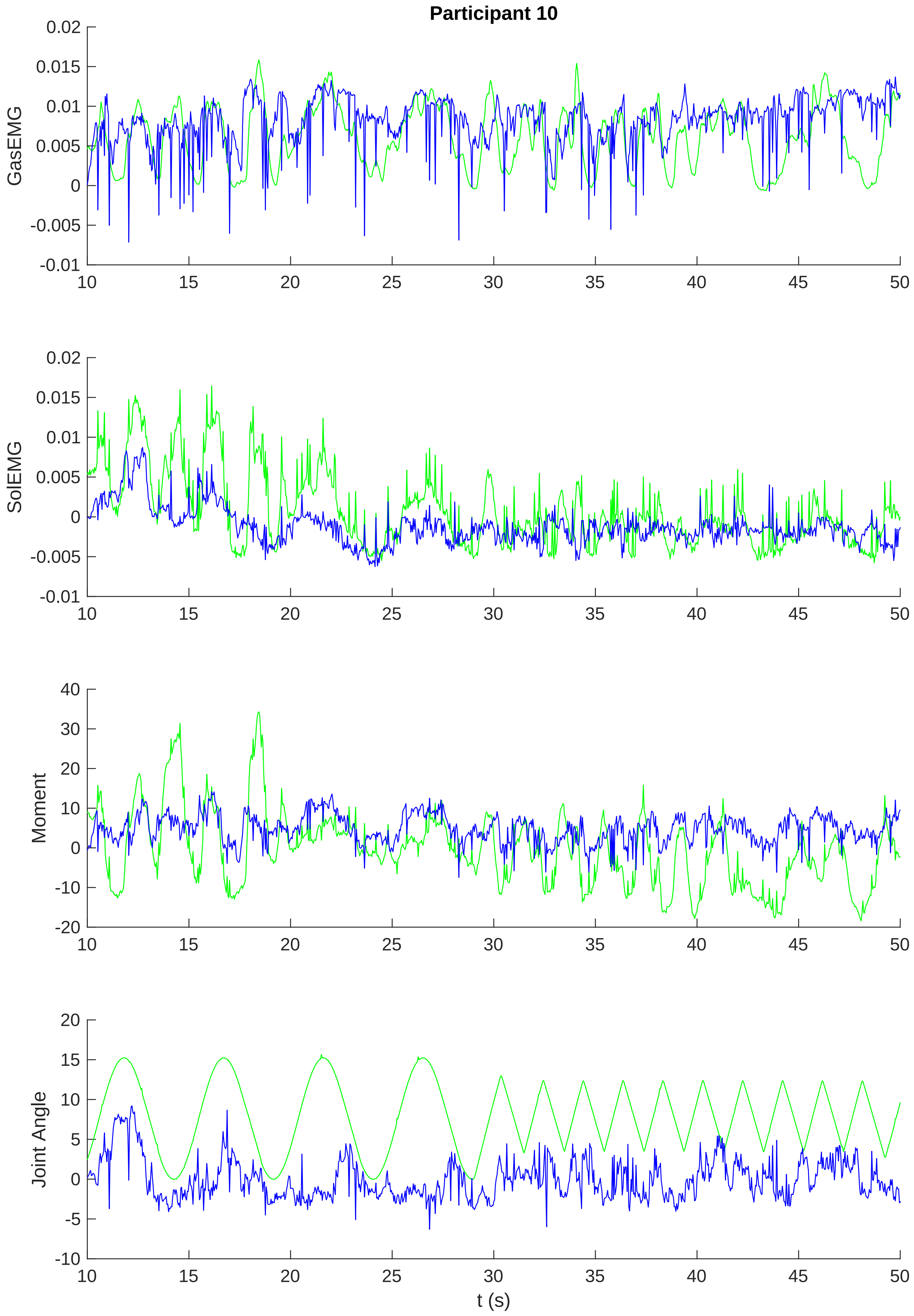
Participant 10
GasEMG
SolEMG
Moment
Joint Angle
t (s)

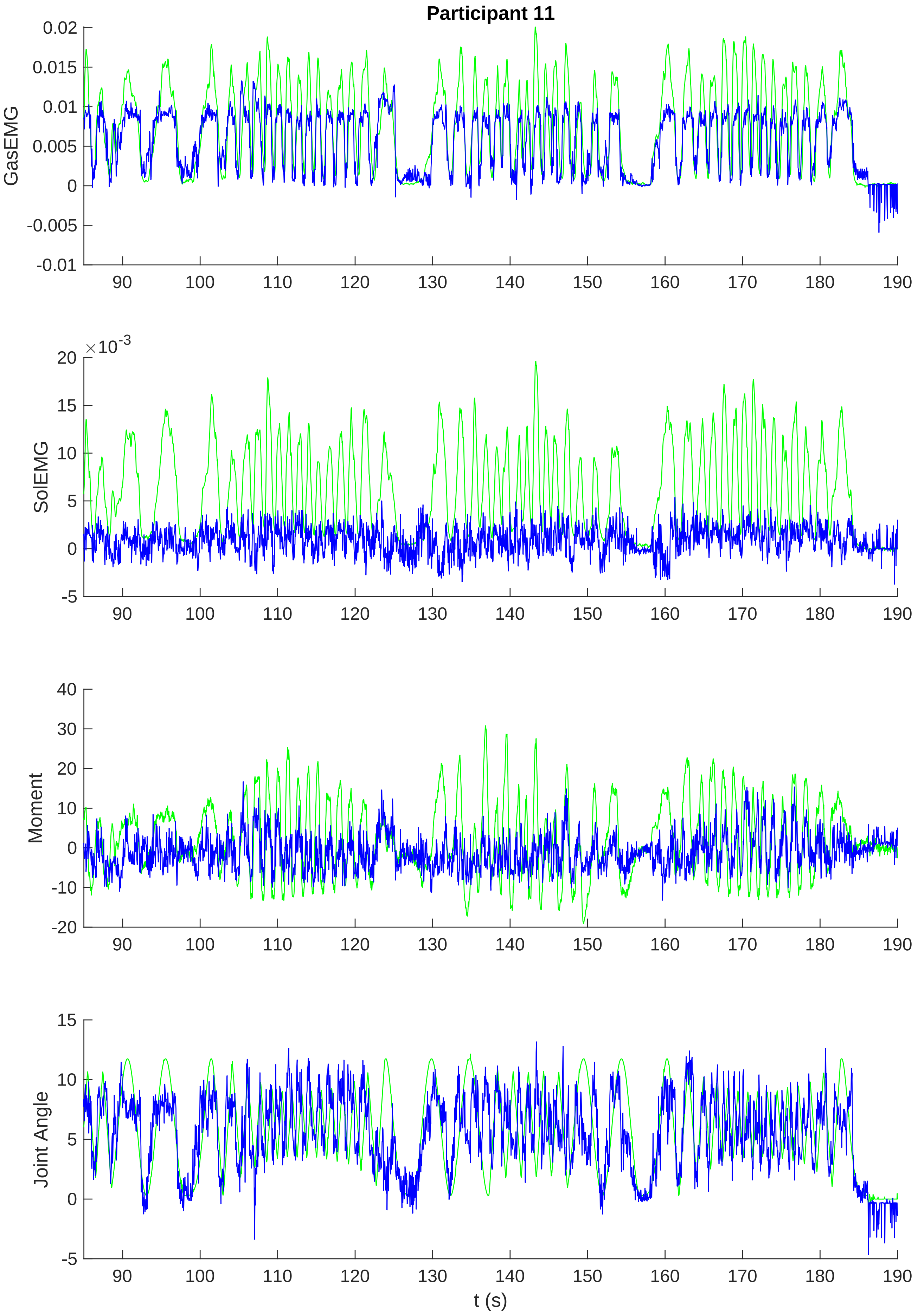

Participant 11
GasEMG
0.02
0.015
0.01
0.005
0
-0.005
-0.01
90
100
110
120
130
140
150
160
170
180
190
SolEMG
×10^-3
20
15
10
5
0
-5
Moment
40
30
20
10
0
-10
-20
Joint Angle
15
10
5
0
-5
t (s)

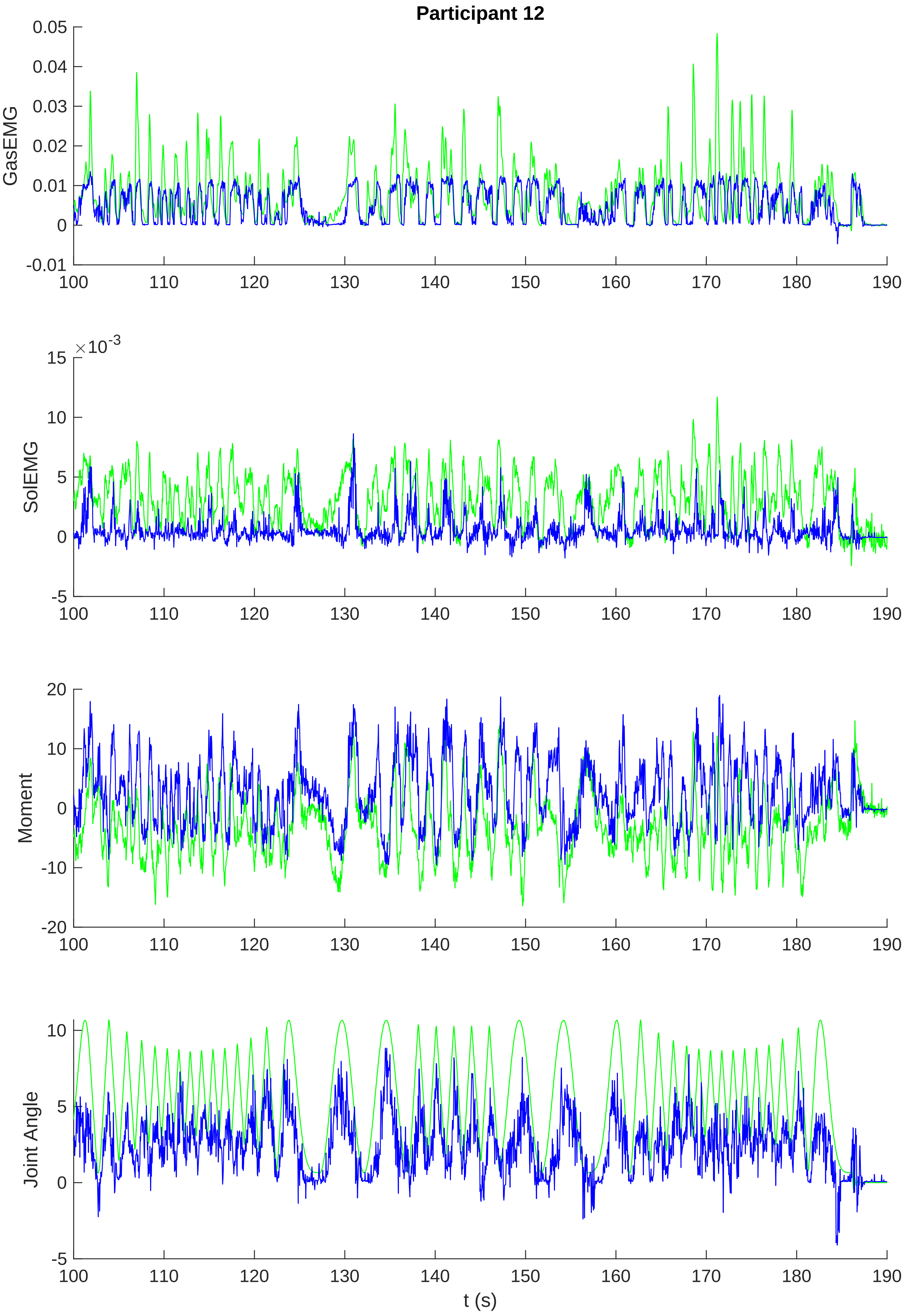

Participant 12
GasEMG
0.05
0.04
0.03
0.02
0.01
0
-0.01
100
110
120
130
140
150
160
170
180
190
SolEMG
×10-3
15
10
5
0
-5
Moment
20
10
0
-10
-20
Joint Angle
10
5
0
-5
t (s)

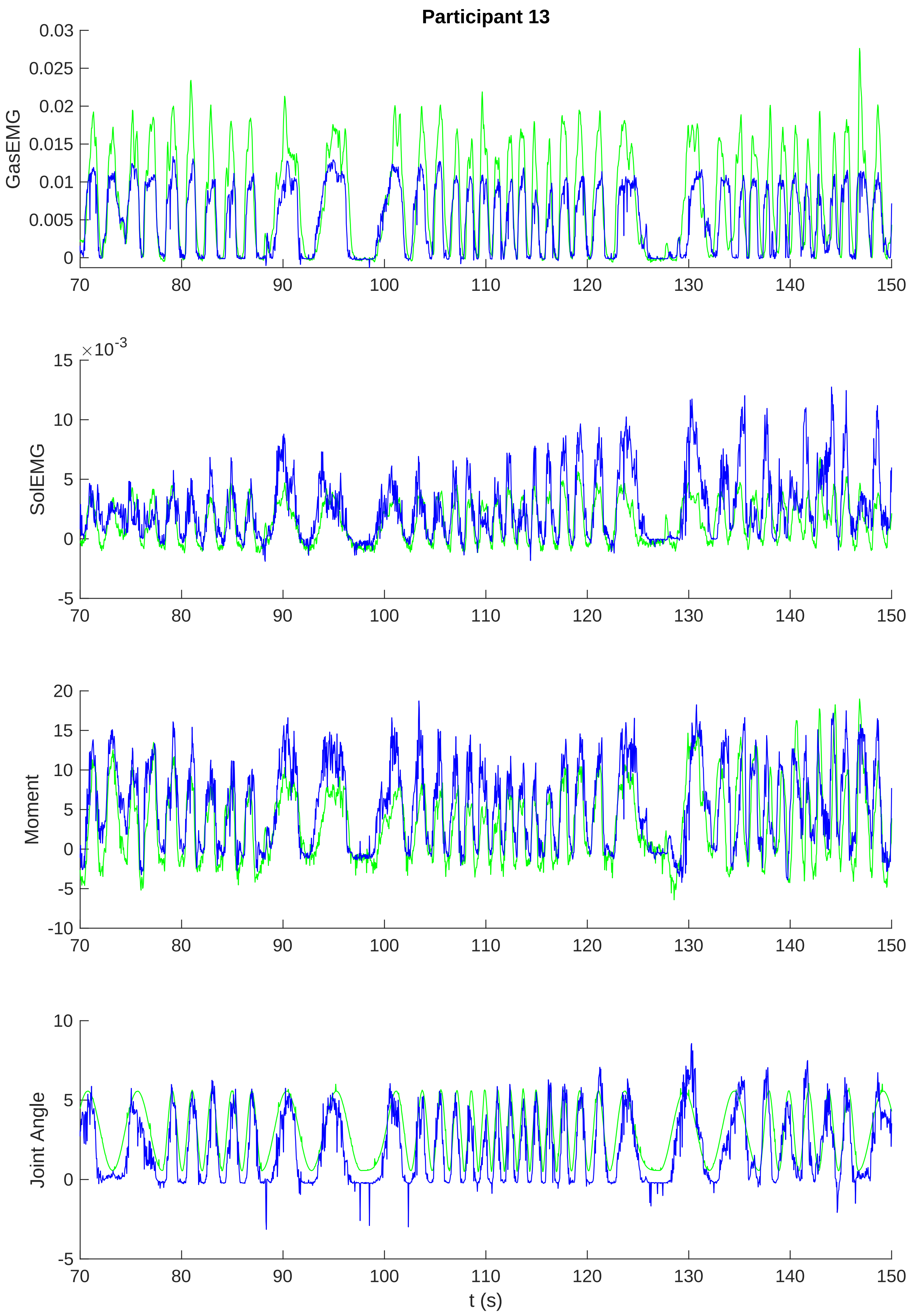

Participant 13
GasEMG
0.03
0.025
0.02
0.015
0.01
0.005
0
70
80
90
100
110
120
130
140
150
SolEMG
×10-3
15
10
5
0
-5
Moment
20
15
10
5
0
-5
-10
Joint Angle
10
5
0
-5
t (s)

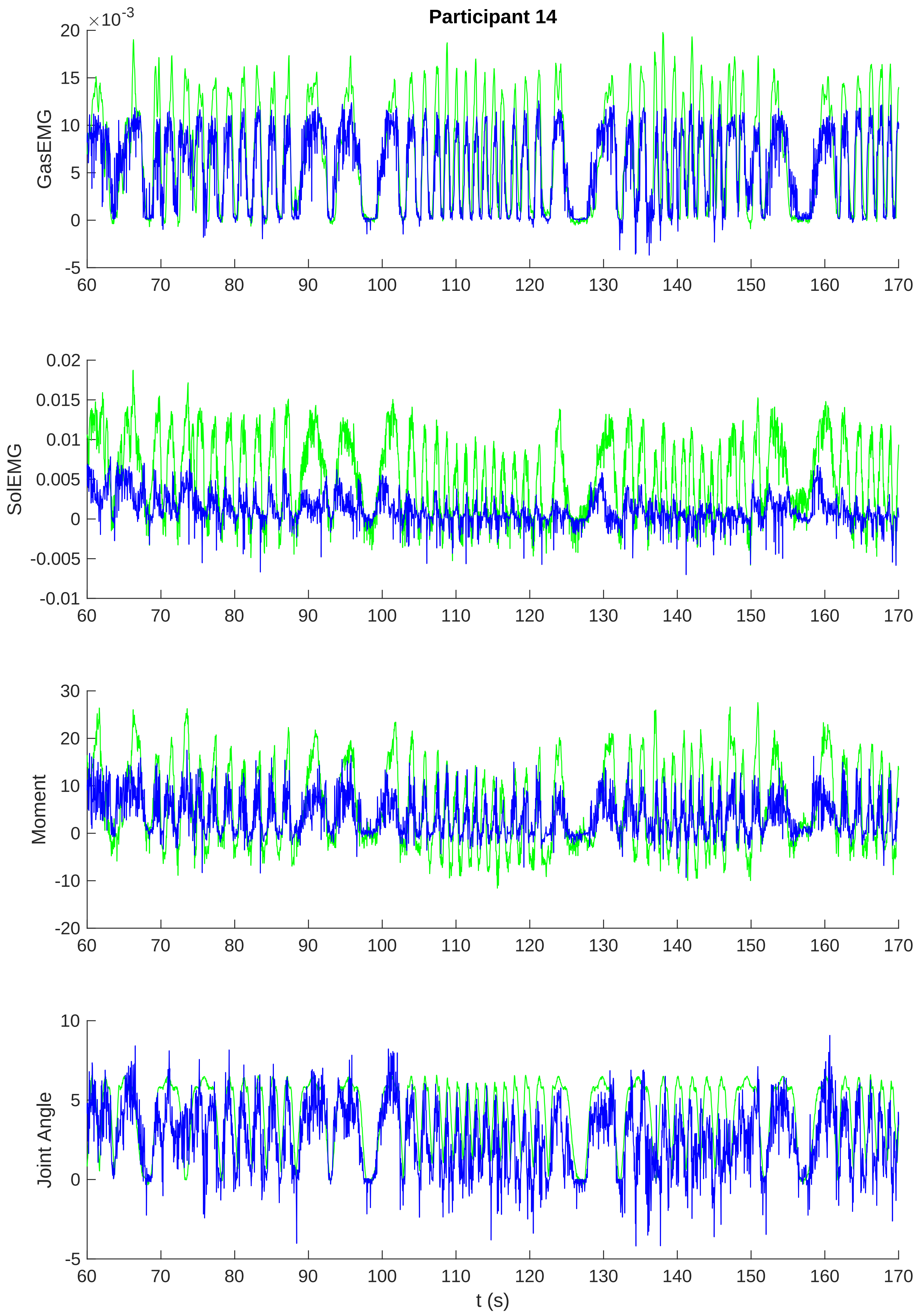
Participant 14
×10^-3
GasEMG
SolEMG
Moment
Joint Angle
t (s)

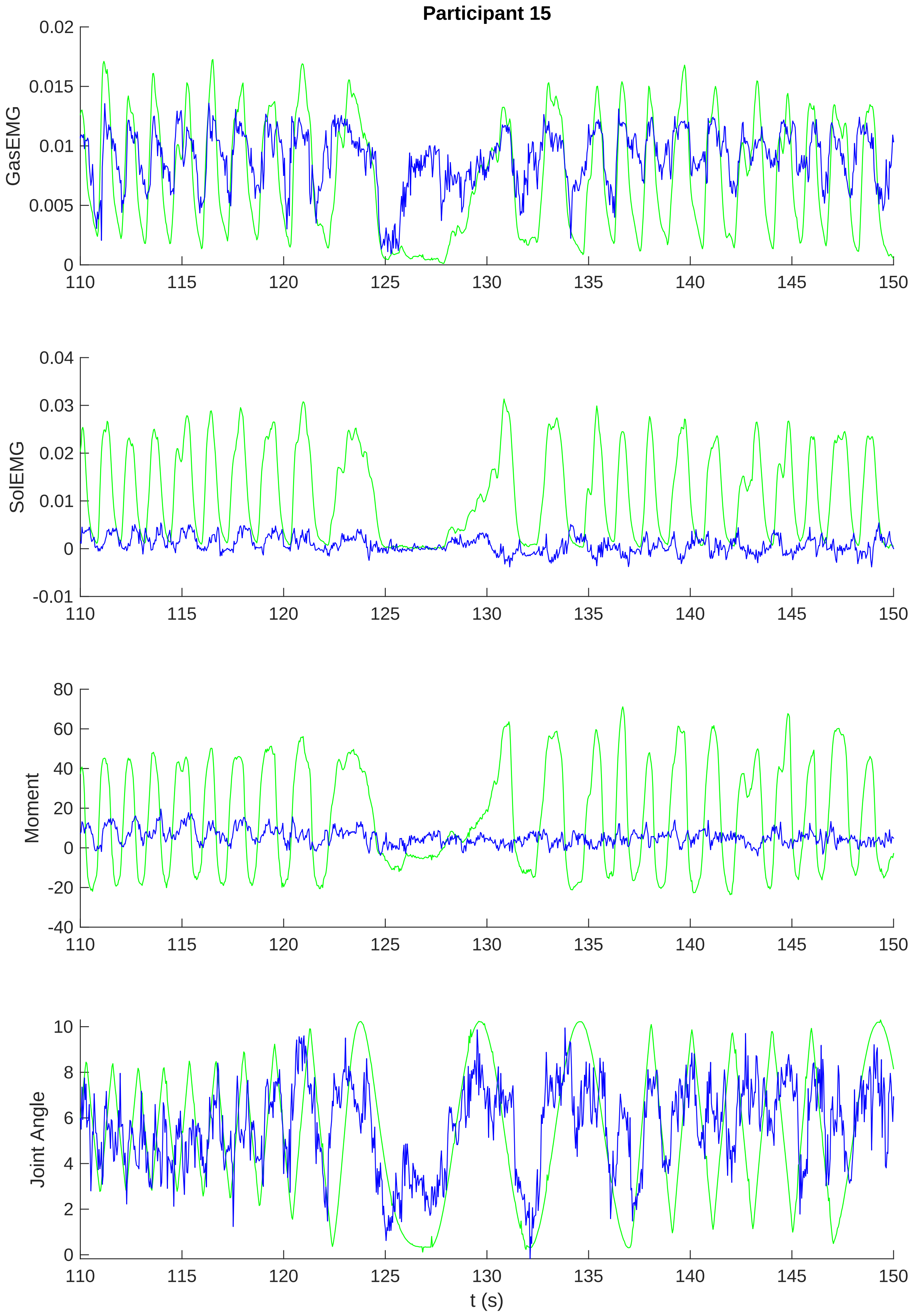

Participant 15
GasEMG
SolEMG
Moment
Joint Angle
t (s)

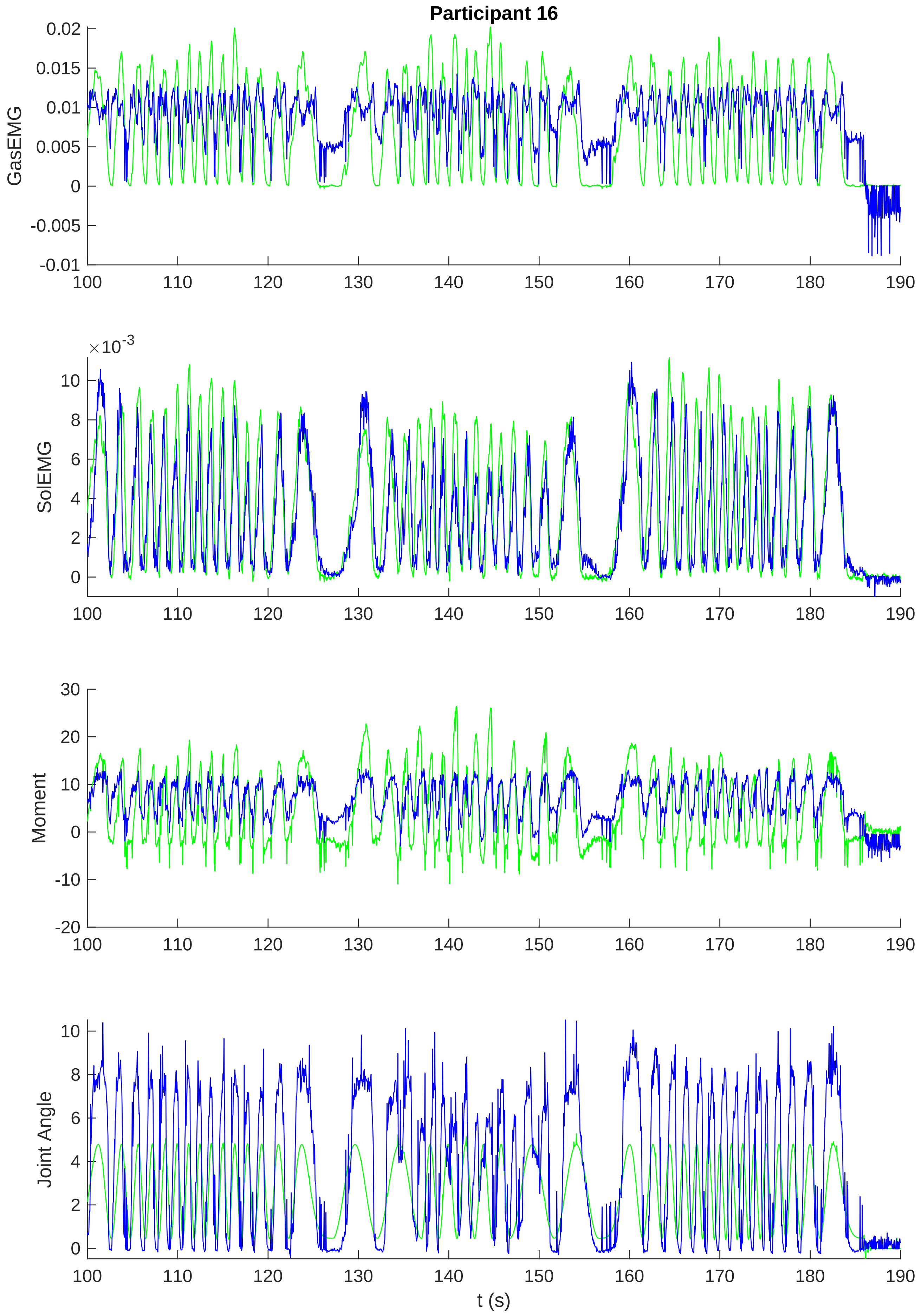
Participant 16
GasEMG
SolEMG
×10^-3
Moment
Joint Angle
t (s)
0.02
0.015
0.01
0.005
0
-0.005
-0.01
100
110
120
130
140
150
160
170
180
190
10
8
6
4
2
0
30
20
-10
-20

Participant 17

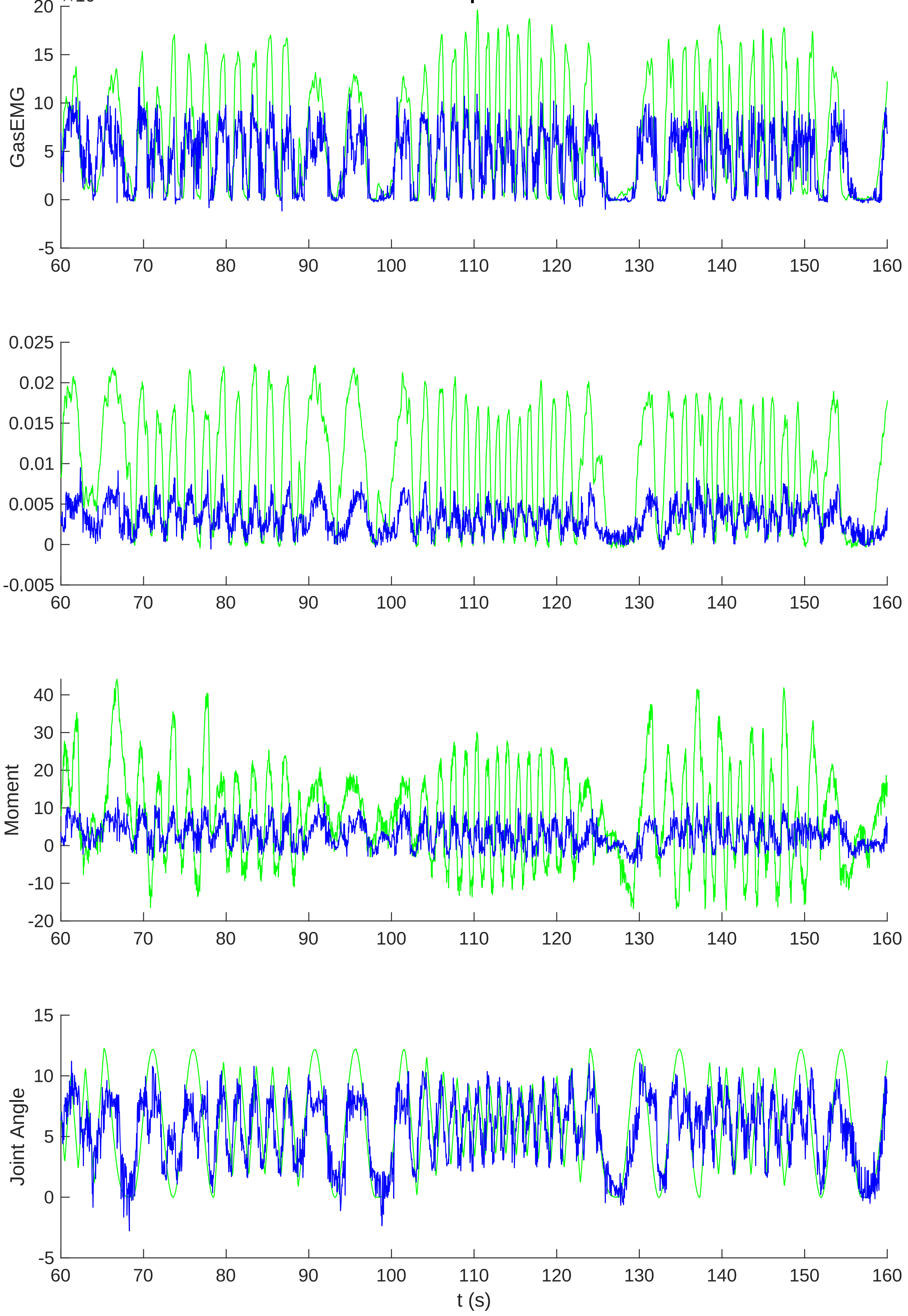

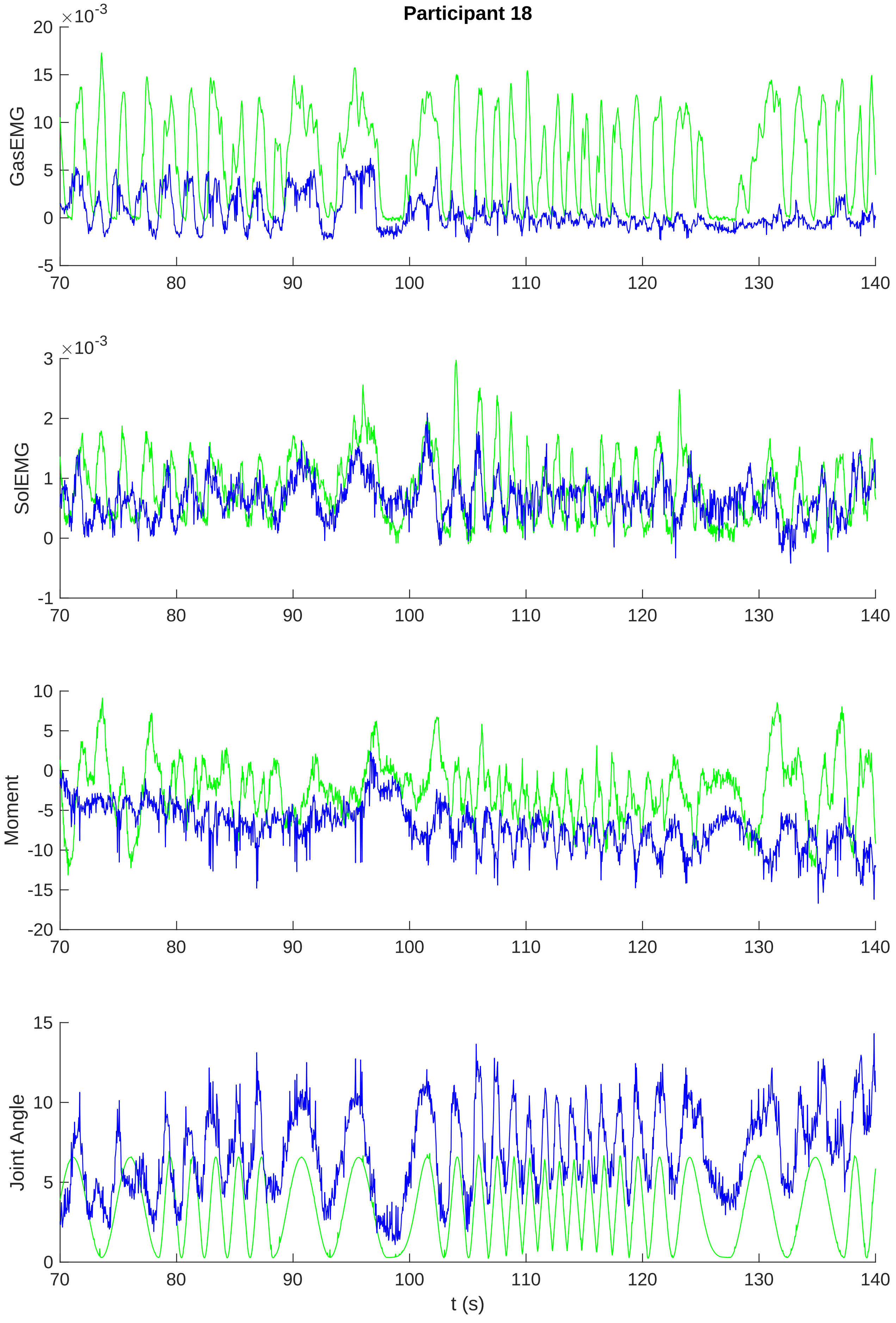

Participant 18
GasEMG
×10^-3
20
15
10
5
0
-5
70
80
90
100
110
120
130
140
SolEMG
×10^-3
3
2
1
0
-1
Moment
10
5
0
-5
-10
-15
-20
Joint Angle
15
10
5
0
t (s)

Participant 19

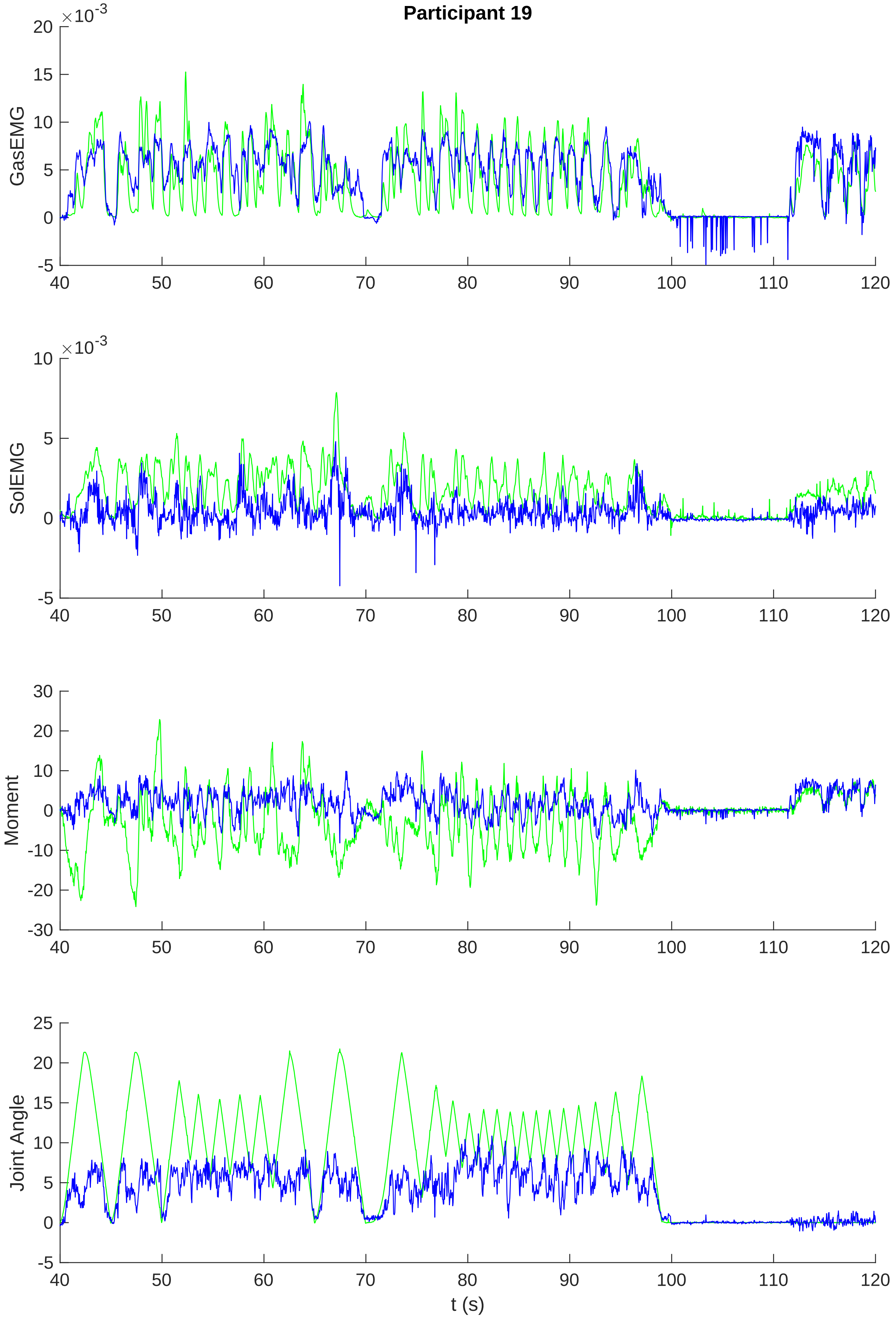

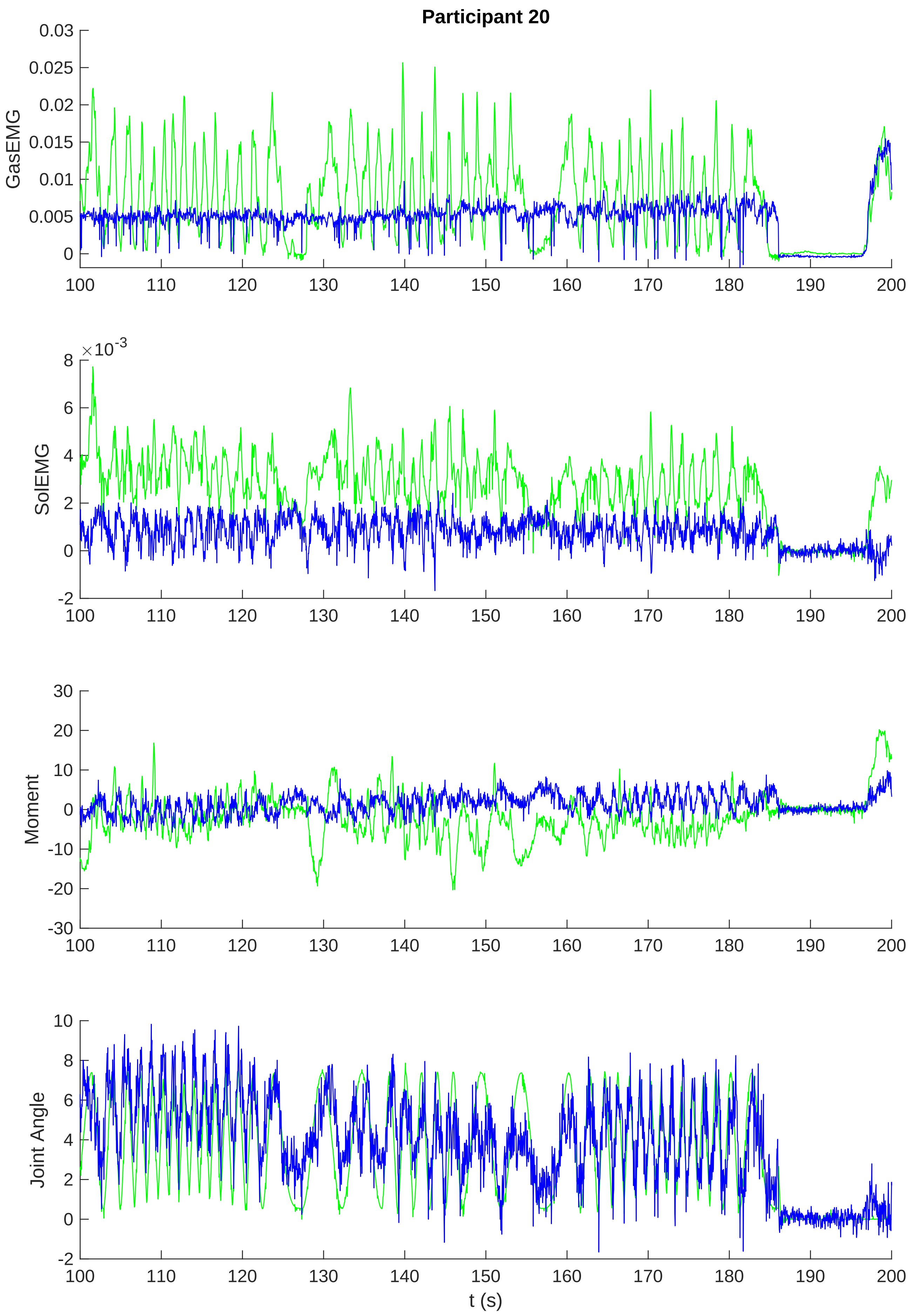
Participant 20
GasEMG
0.03
0.025
0.02
0.015
0.01
0.005
0
SolEMG
×10-3
8
6
4
2
0
-2
Moment
30
20
10
0
-10
-20
-30
Joint Angle
10
8
6
4
2
0
-2
100
110
120
130
140
150
160
170
180
190
200
t (s)

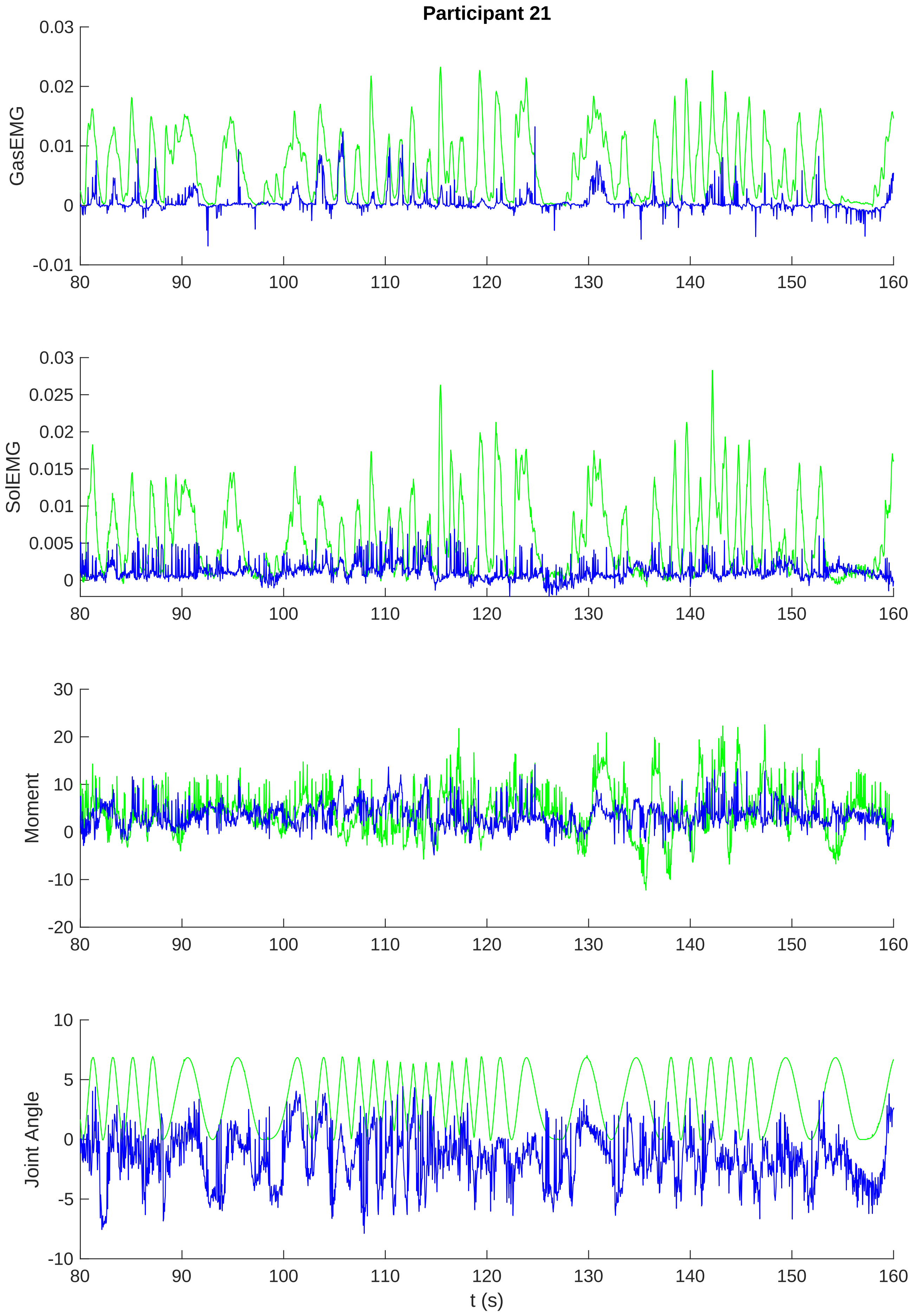

Participant 21
GasEMG
0.03
0.02
0.01
0
-0.01
80
90
100
110
120
130
140
150
160
SolEMG
0.03
0.025
0.02
0.015
0.01
0.005
0
Moment
30
20
10
0
-10
-20
Joint Angle
10
5
0
-5
-10
t (s)

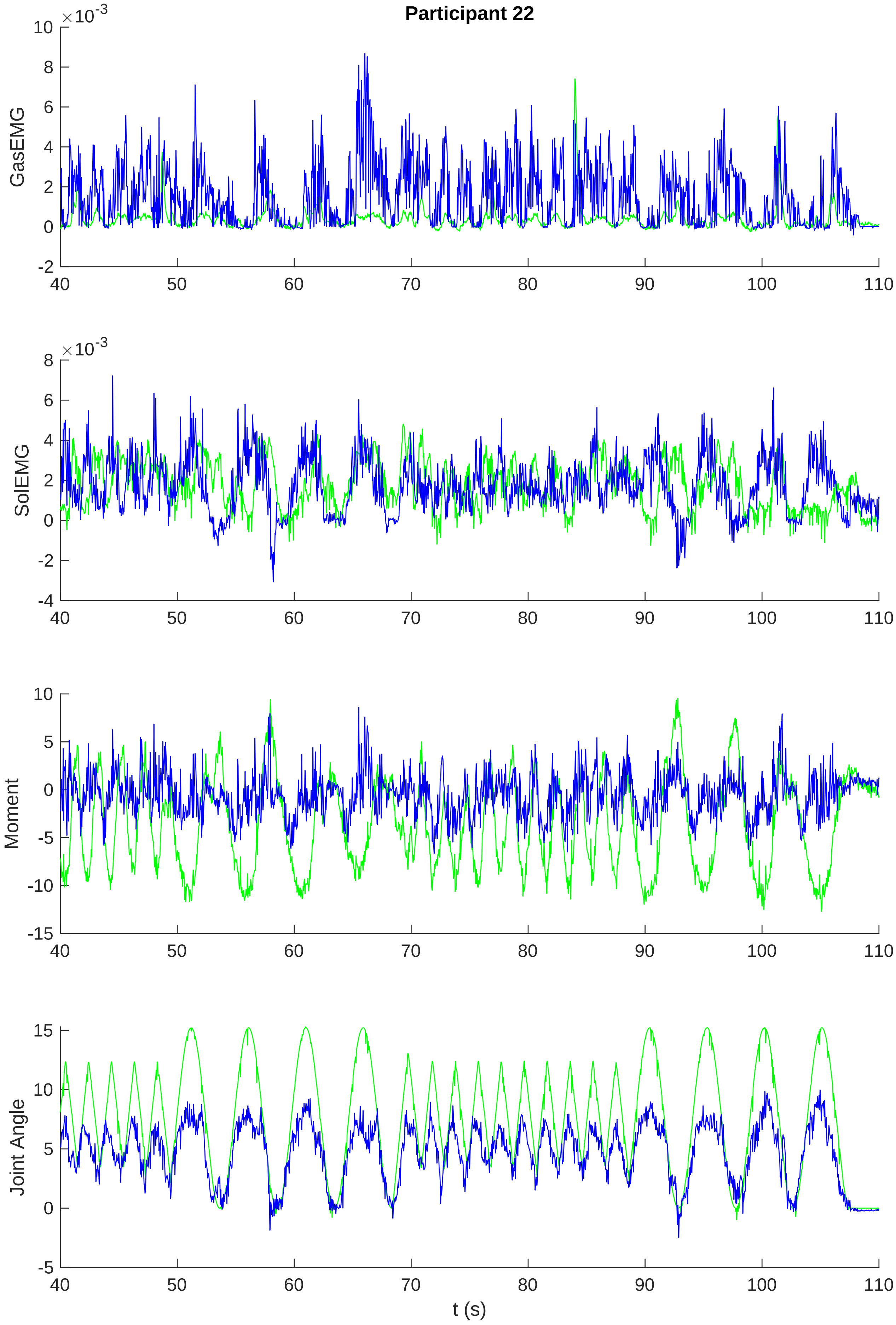
Participant 22
GasEMG
×10^-3
10
8
6
4
2
0
-2
SolEMG
×10^-3
8
6
4
2
0
-2
-4
Moment
10
5
0
-5
-10
-15
Joint Angle
15
10
5
0
-5
40
50
60
70
80
90
100
110
t (s)

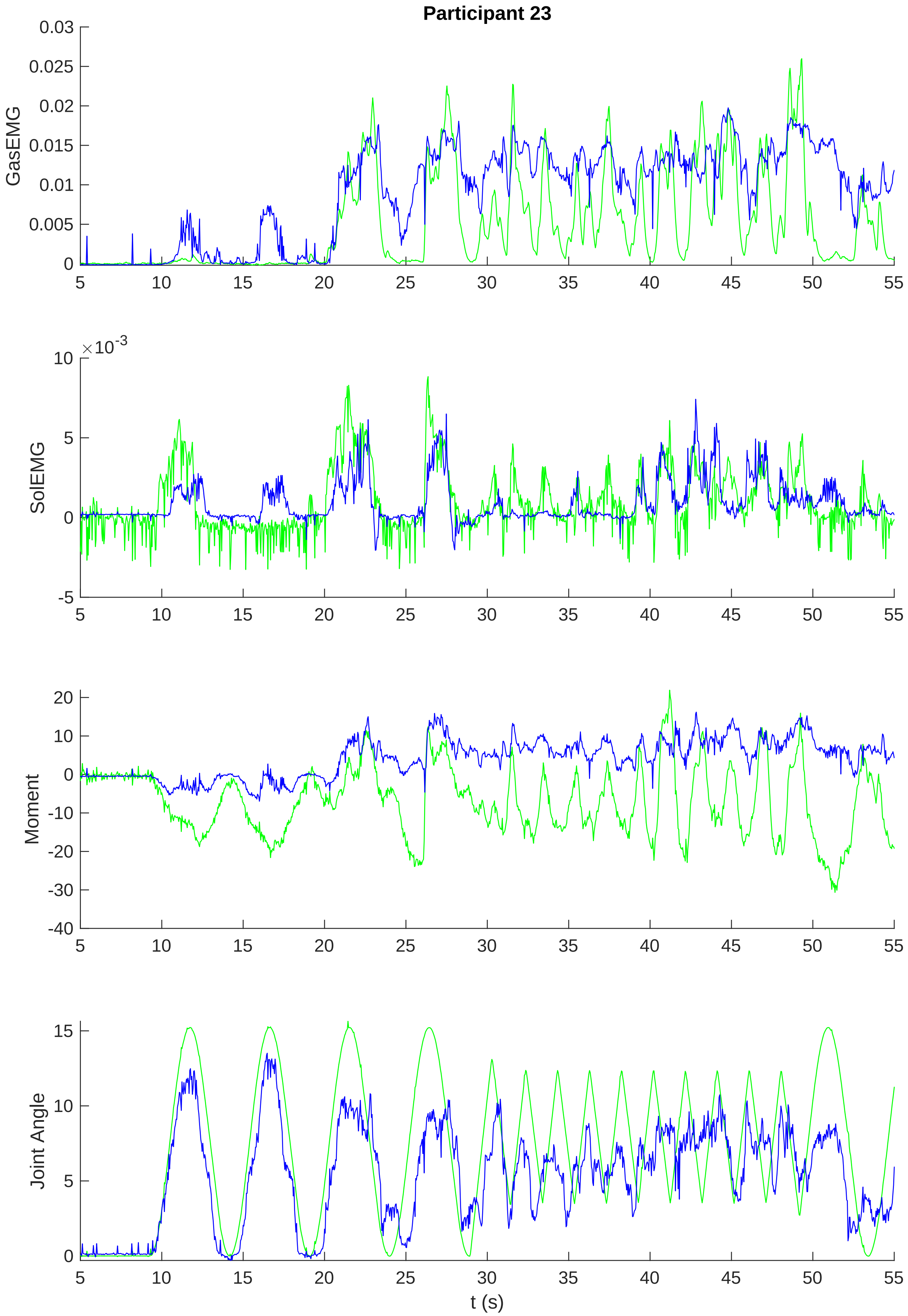
Participant 23
GasEMG
0.03
0.025
0.02
0.015
0.01
0.005
0
5
10
15
20
25
30
35
40
45
50
55
SolEMG
×10^-3
10
5
0
-5
Moment
20
10
0
-10
-20
-30
-40
Joint Angle
15
10
5
0
t (s)

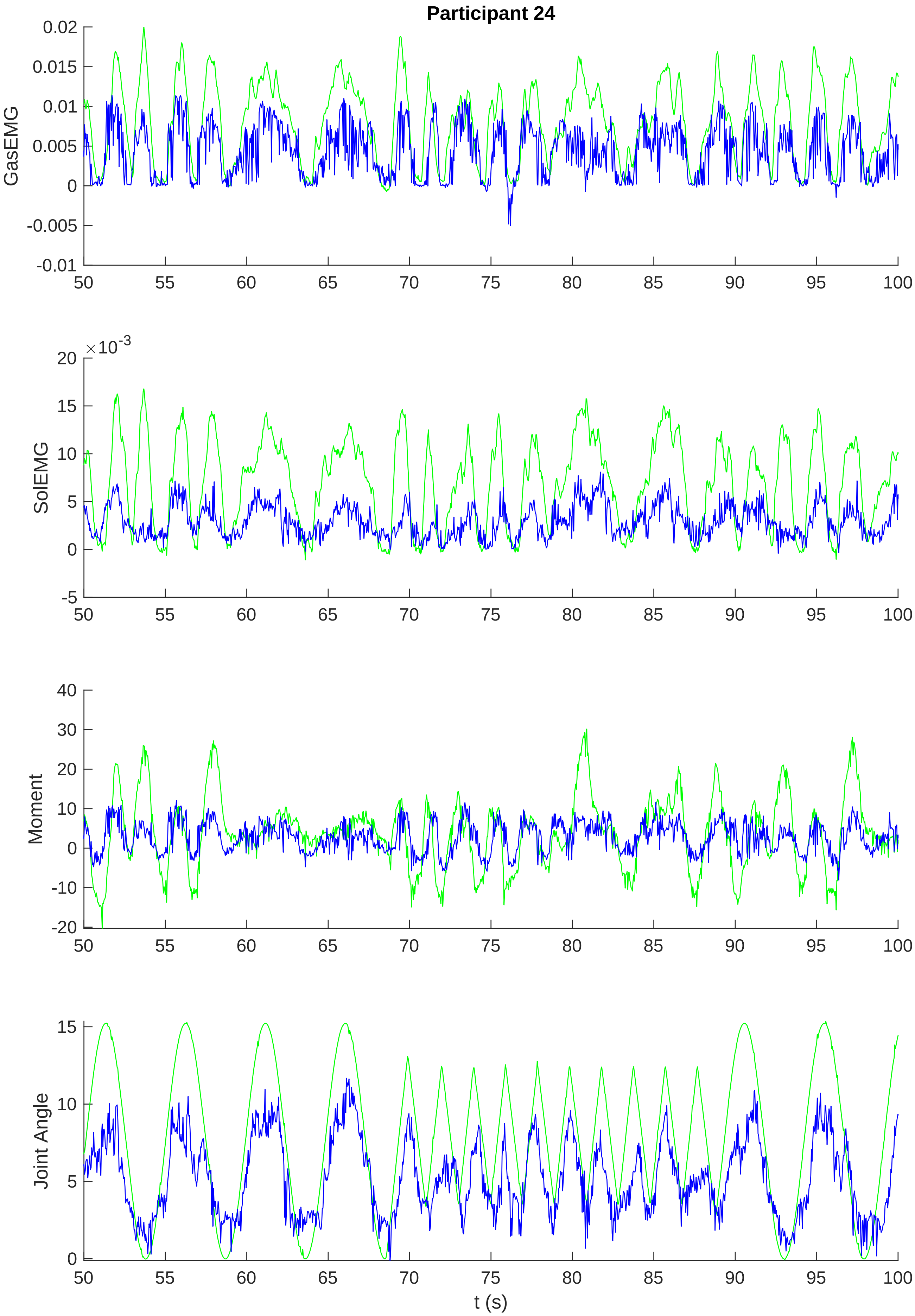
Participant 24
GasEMG
0.02
0.015
0.01
0.005
0
-0.005
-0.01
50
55
60
65
70
75
80
85
90
95
100
SolEMG
×10^-3
20
15
10
5
0
-5
Moment
40
30
20
10
0
-10
-20
Joint Angle
15
10
5
0
t (s)

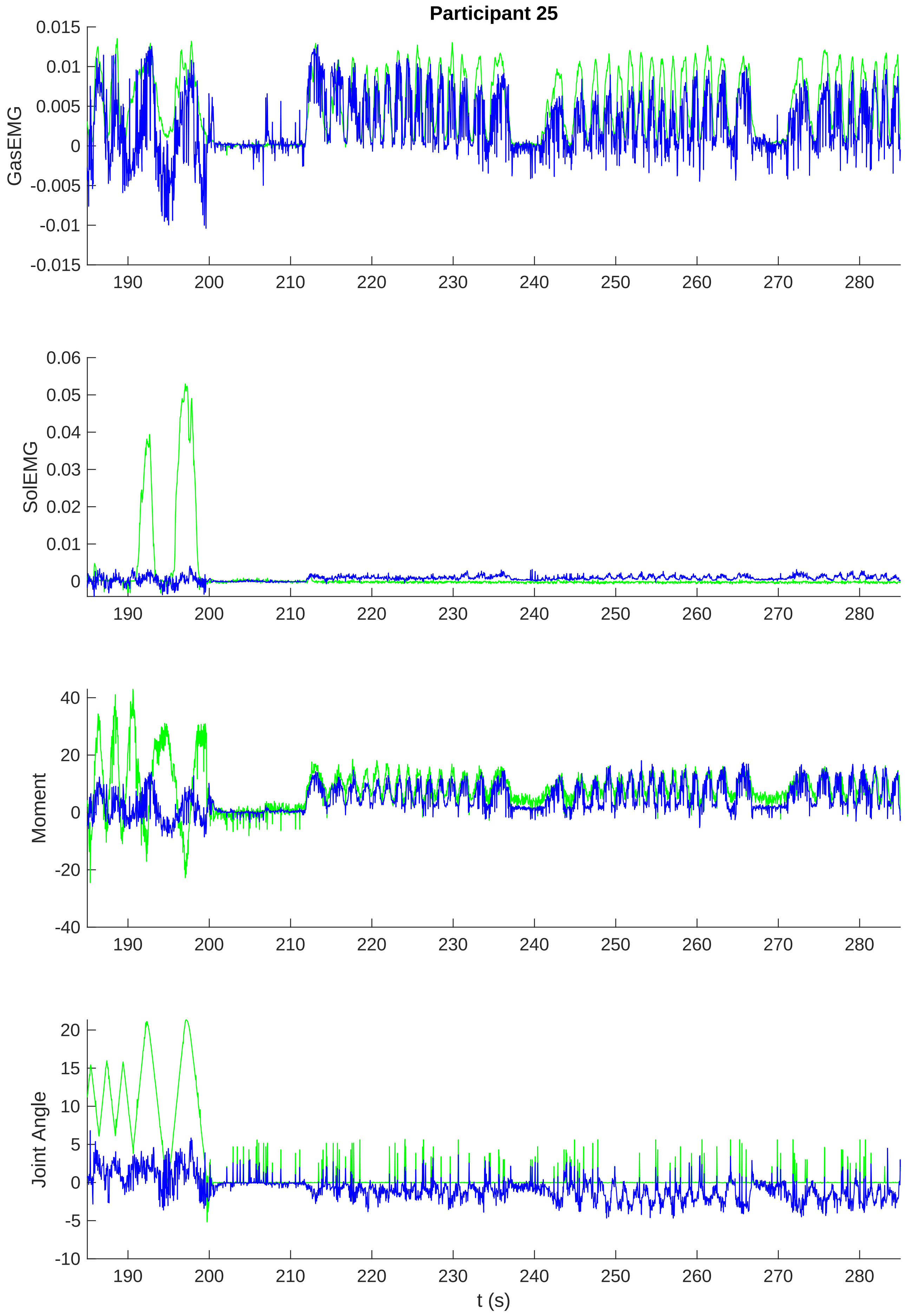
Participant 25
GasEMG
0.015
0.01
0.005
0
-0.005
-0.01
-0.015
SolEMG
0.06
0.05
0.04
0.03
0.02
0.01
Moment
40
20
-20
-40
Joint Angle
15
10
5
-5
-10
190
200
210
220
230
240
250
260
270
280
t (s)

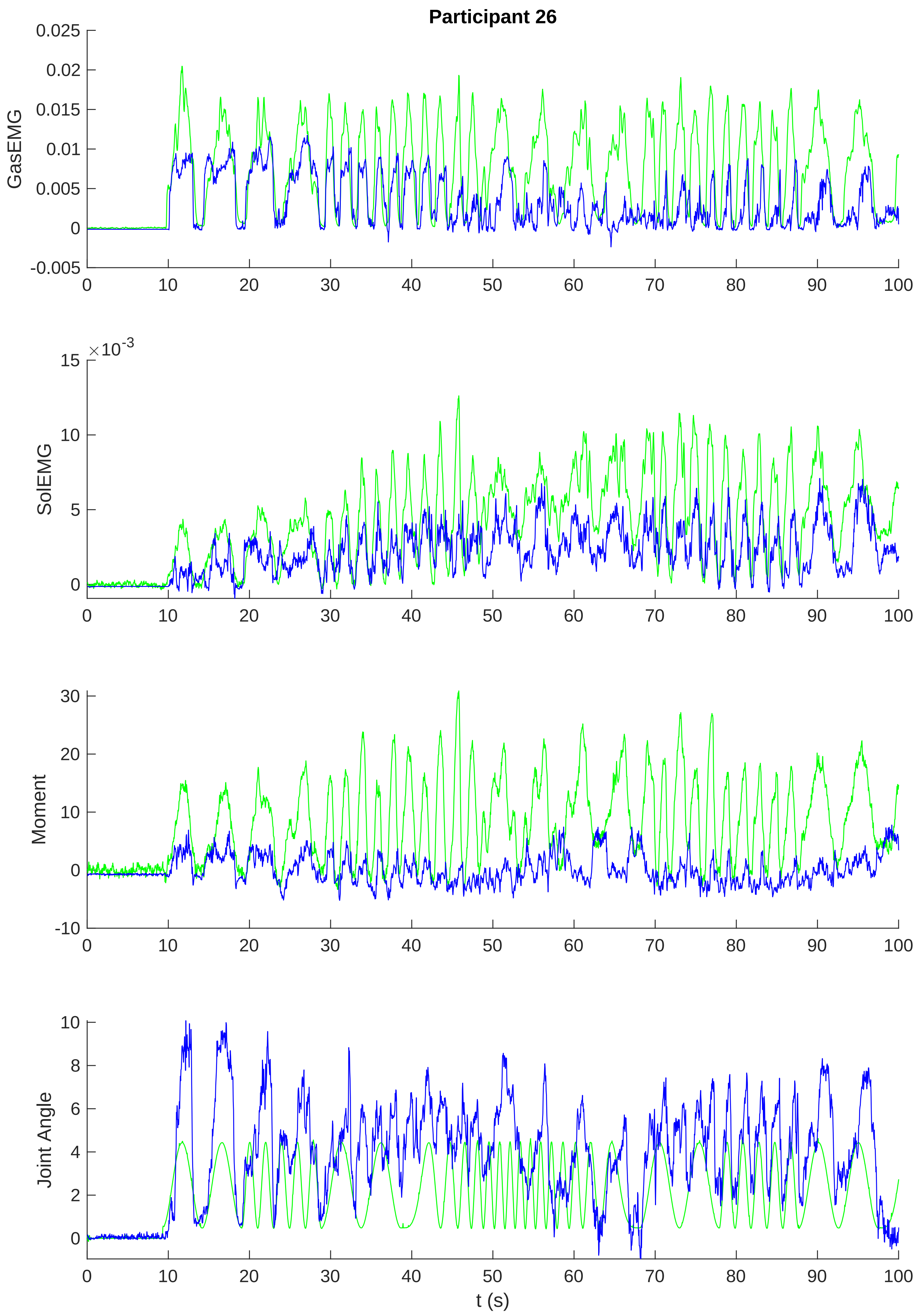
Participant 26
GasEMG
0.025
0.02
0.015
0.01
0.005
0
-0.005
SolEMG
×10^-3
15
10
5
0
Moment
30
20
10
0
-10
Joint Angle
10
8
6
4
2
0
0 10 20 30 40 50 60 70 80 90 100
t (s)

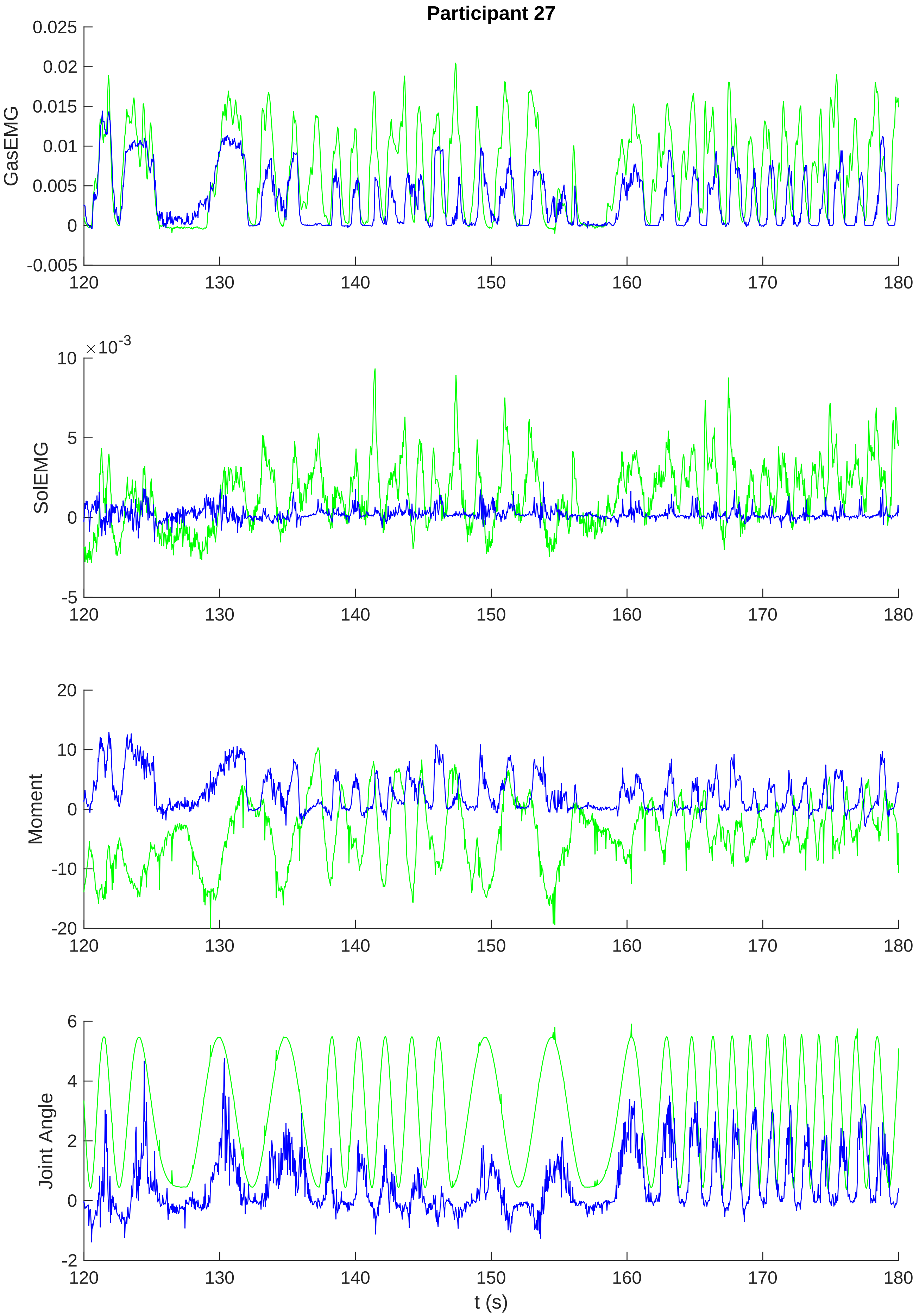
Participant 27
GasEMG
0.025
0.02
0.015
0.01
0.005
0
-0.005
SolEMG
×10-3
10
5
0
-5
Moment
20
10
0
-10
-20
Joint Angle
6
4
2
0
-2
120
130
140
150
160
170
180
t (s)

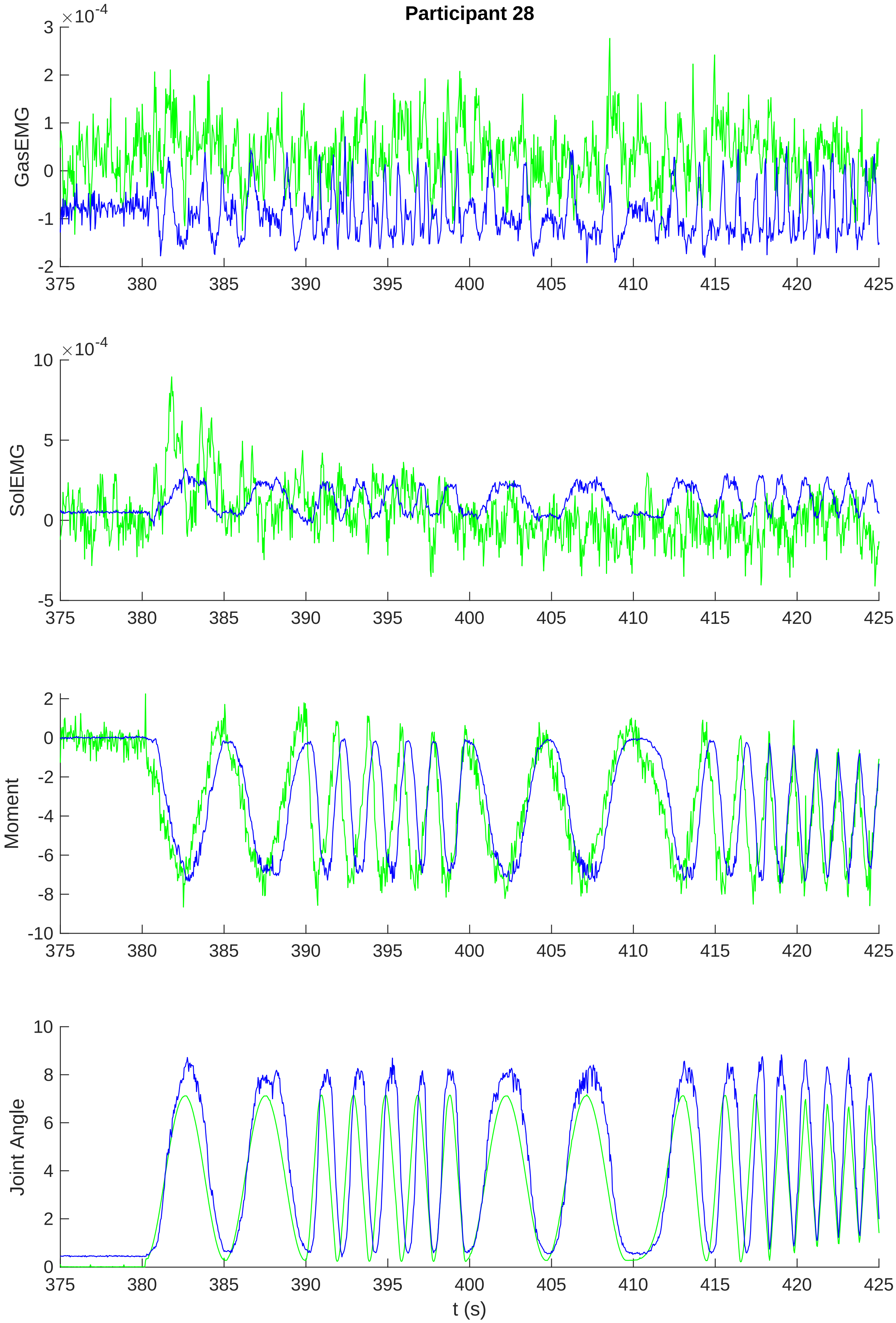

Participant 28
×10^-4
GasEMG
3
2
1
0
-1
-2
375 380 385 390 395 400 405 410 415 420 425
×10^-4
SolEMG
10
5
0
-5
375 380 385 390 395 400 405 410 415 420 425
Moment
2
0
-2
-4
-6
-8
-10
375 380 385 390 395 400 405 410 415 420 425
Joint Angle
10
8
6
4
2
0
375 380 385 390 395 400 405 410 415 420 425
t (s)

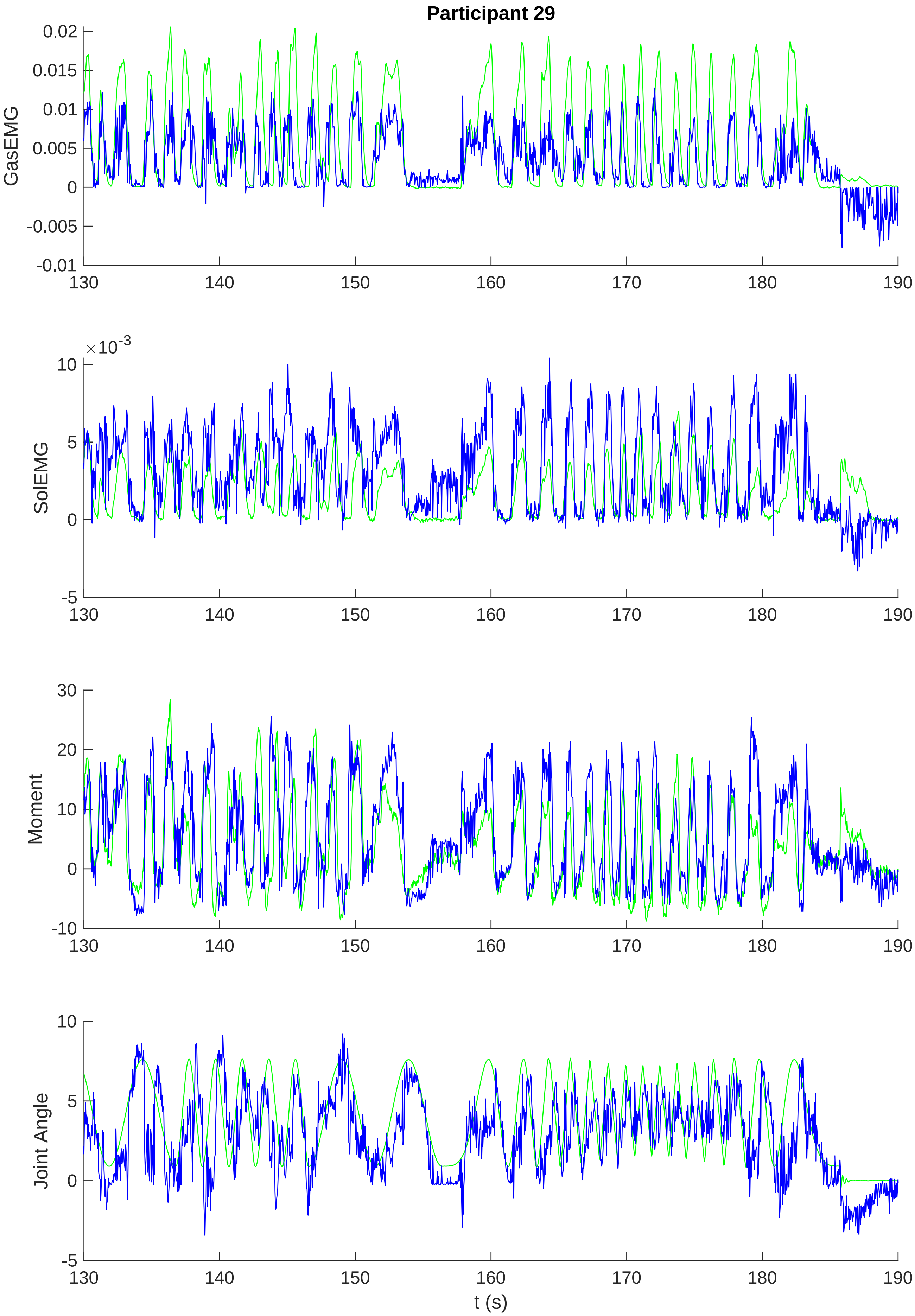
Participant 29
GasEMG
0.02
0.015
0.01
0.005
0
-0.005
-0.01
130
140
150
160
170
180
190
SolEMG
×10-3
10
5
0
-5
Moment
30
20
10
0
-10
Joint Angle
10
5
0
-5
t (s)

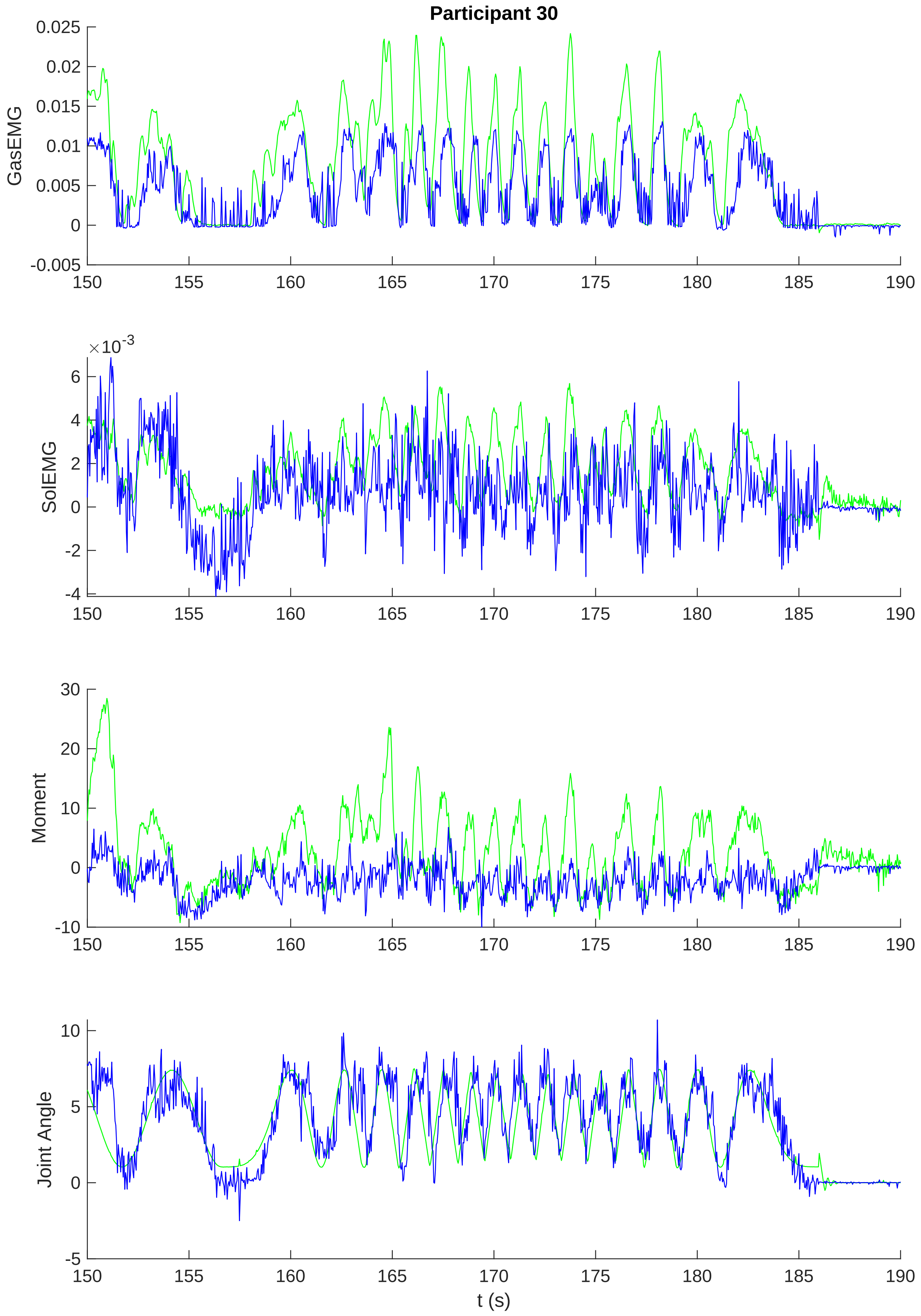

Participant 30
GasEMG
0.025
0.02
0.015
0.01
0.005
0
-0.005
150
155
160
165
170
175
180
185
190
SolEMG
×10^-3
6
4
2
0
-2
-4
Moment
30
20
10
0
-10
Joint Angle
10
5
0
-5
t (s)

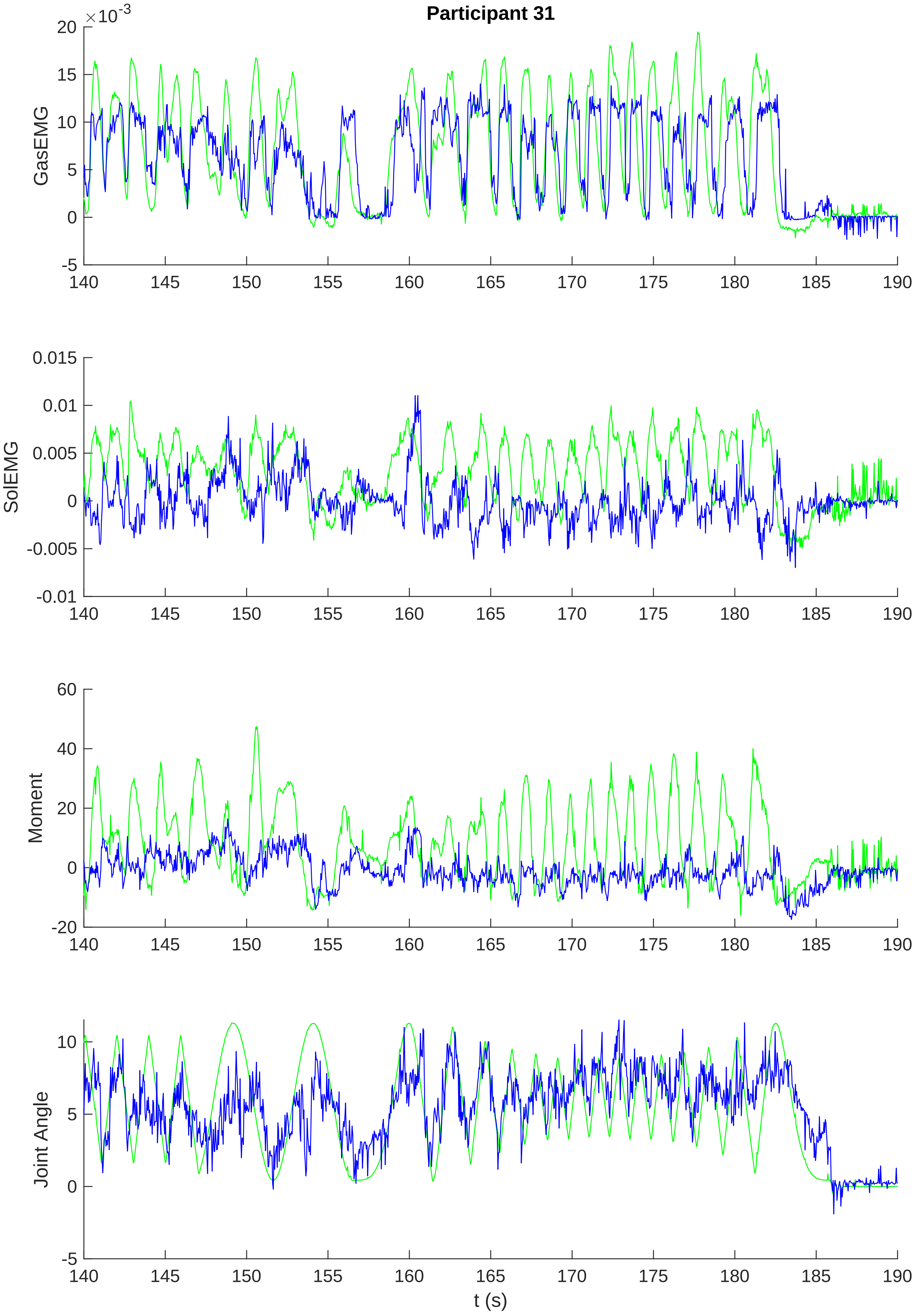

Participant 31
×10^-3
GasEMG
SolEMG
Moment
Joint Angle
t (s)

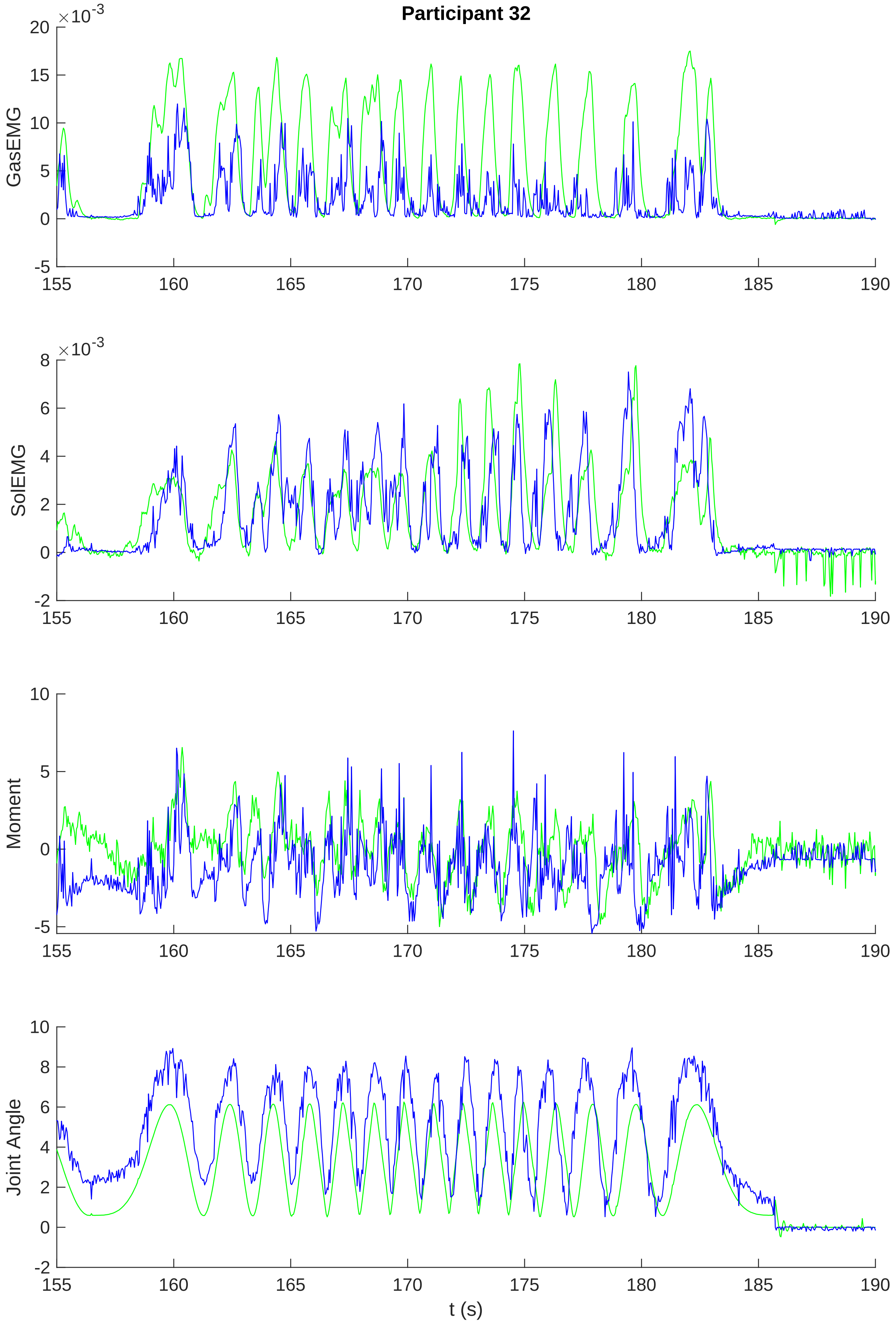
Participant 32
GasEMG
×10^-3
SolEMG
×10^-3
Moment
Joint Angle
t (s)
